# Azidophenyl as a click-transformable redox label of DNA suitable for electrochemical detection of DNA–protein interactions†
†Electronic supplementary information (ESI) available: Additional figures of PAGE analyses of PEX experiments, additional electrochemistry figures, copies of MALDI spectra, copies of NMR spectra. See DOI: 10.1039/c4sc01906g



**DOI:** 10.1039/c4sc01906g

**Published:** 2014-09-16

**Authors:** Jana Balintová, Jan Špaček, Radek Pohl, Marie Brázdová, Luděk Havran, Miroslav Fojta, Michal Hocek

**Affiliations:** a Institute of Organic Chemistry and Biochemistry , Academy of Sciences of the Czech Republic , Gilead & IOCB Research Center , Flemingovo nam. 2 , CZ-16610 Prague 6 , Czech Republic . Email: hocek@uochb.cas.cz; b Institute of Biophysics , v.v.i. Academy of Sciences of the Czech Republic , Kralovopolska 135 , 61265 Brno , Czech Republic . Email: fojta@ibp.cz; c Central European Institute of Technology , Masaryk University , Kamenice 753/5 , CZ-625 00 Brno , Czech Republic; d Department of Organic Chemistry , Faculty of Science , Charles University in Prague , Hlavova 8 , CZ-12843 Prague 2 , Czech Republic

## Abstract

New redox labelling of DNA by an azido group which can be chemically transformed to nitrophenyltriazole or silenced to phenyltriazole was developed and applied to the electrochemical detection of DNA–protein interactions. 5-(4-Azidophenyl)-2′-deoxycytidine and 7-(4-azidophenyl)-7-deaza-2′-deoxyadenosine nucleosides were prepared by aqueous-phase Suzuki cross-coupling and converted to nucleoside triphosphates (dNTPs) which served as substrates for incorporation into DNA by DNA polymerase. The azidophenyl-modified nucleotides and azidophenyl-modified DNA gave a strong signal in voltammetric studies, at –0.9 V, due to reduction of the azido function. The Cu-catalyzed click reaction of azidophenyl-modified nucleosides or azidophenyl-modified DNA with 4-nitrophenylacetylene gave nitrophenyl-substituted triazoles, exerting a reduction peak at –0.4 V under voltammetry, whereas the click reaction with phenylacetylene gave electrochemically silent phenyltriazoles. The transformation of the azidophenyl label to nitrophenyltriazole was used for electrochemical detection of DNA–protein interactions (p53 protein) since only those azidophenyl groups in the parts of the DNA not shielded by the bound p53 protein were transformed to nitrophenyltriazoles, whereas those covered by the protein were not.

## Abstract 2

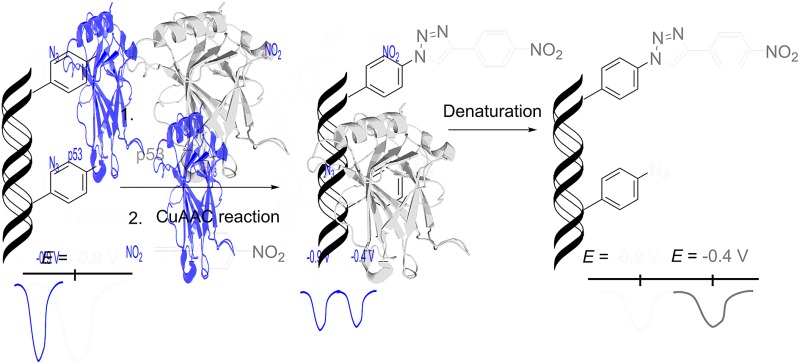
A new azido-based DNA redox label which can be transformed into nitrophenyltriazole by a CuAAC click reaction was developed. It was used for the mapping of DNA–protein interactions with electrochemical detection.

## Introduction

Electrochemical detection of redox-labelled DNA^1^ is an alternative to fluorescence techniques for DNA sequencing and diagnostics. However, despite the extensive research and number of available oxidizable or reducible labels,^2^ the redox labelling of DNA often suffers from problems with sensitivity, stability and cross-reactivity of the labels. On the other hand, the use of several labels offers access to direct redox coding of DNA.^3^ To the best of our knowledge, applications of redox labelling and electrochemistry for studying DNA–protein interactions are still relatively scarce, limited to techniques based on changes in DNA-mediated charge transfer upon the protein binding (developed by J. K. Barton’s group^4^) and our recent studies utilizing immunoprecipitation at magnetic beads.^5^ Most known methods for detection and footprinting of those interactions^6^ are based on specific enzymatic or chemical cleavage of DNA.^7^

Copper(i)-catalyzed azide–alkyne cycloaddition (CuAAC or click reaction) is one of the most important bioorthogonal reactions^8^ and has been widely used for modifications of oligonucleotides (ONs) and DNA.^9^ Due to better compatibility with phosphoramidite synthesis, triphosphorylation and polymerase incorporations, base-modified nucleotides bearing an acetylene are typically incorporated into ON or DNA and are then clicked with an azido-derivative of the other component.^10^ Only recently, 5-azidomethyl-dUTP has been synthesized and used for metabolic labelling through polymerase incorporation and click reaction with a fluorescent acetylene.^11^ We have envisaged the azido group^12^ as a new redox label suitable for electrochemical detection but also transformable to another redox label through the click reaction.

## Results and discussion

### Synthesis of modified nucleosides and triphosphates

**Scheme 1:**
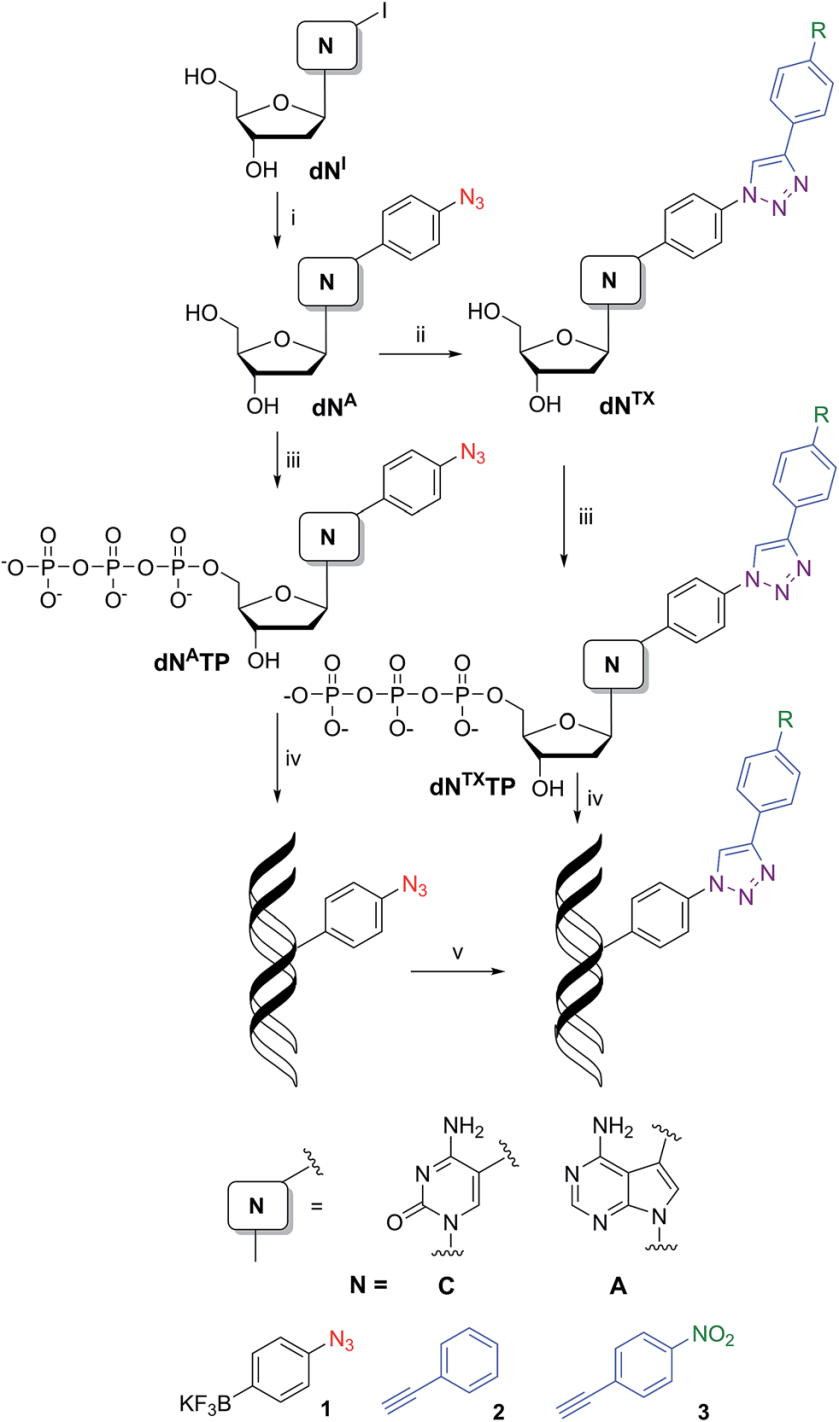
Synthesis of modified nucleosides: (i) Suzuki–Miyaura cross-coupling: **1**, PdCl_2_(dppf), Cs_2_CO_3_, MeOH, 2 h, 80 °C; (ii) CuAAC: **2** (or **3**), sodium ascorbate, CuSO_4_·5H_2_O, *t*BuOH–H_2_O (1 : 1), 12 h, rt; (iii) triphosphorylation of modified nucleosides: (1) PO(OMe)_3_, POCl_3_, 0 °C; (2) (NHBu_3_)_2_H_2_P_2_O_7_, Bu_3_N, DMF, 0 °C; (3) TEAB; (iv) PEX experiment; (v) azide–alkyne Huisgen cycloaddition: **2** (or **3**), sodium ascorbate, CuBr, TBTA ligand, *t*BuOH–DMSO (1 : 3), 2 h, 37 °C.

Our strategy for the synthesis of labelled ONs and DNA relied on polymerase-catalyzed incorporations^13^ of base-modified nucleotides. The modified dNTPs, required as substrates, are available through triphosphorylation of modified nucleosides. The synthesis of the azidophenyl-modified nucleosides was based on a Suzuki–Miyaura cross-coupling reaction of the unprotected halogenated nucleosides 5-iodocytidine (**dC^I^**) and 7-deaza-7-iodoadenosine (**dA^I^**) with 4-azidophenyltrifluoroborate (**1**).^14^ The reactions were performed in the presence of a PdCl_2_(dppf) catalyst and Cs_2_CO_3_ in MeOH and gave the desired modified nucleosides (**dC^A^** and **dA^A^**) in good yields of 58 and 63% (Scheme 1, Table 1, entries 1 and 2). A Huisgen–Sharpless CuAAC reaction^15^ between the azidophenyl-modified nucleosides (**dC^A^** and **dA^A^**) and an alkyne (phenylacetylene **2** or 1-ethynyl-4-nitrobenzene **3**) in the presence of copper(ii) sulfate pentahydrate and sodium ascorbate as a reducing agent in *t*BuOH–H_2_O (1 : 1) was used for the synthesis of 1,4-disubstituted 1,2,3-triazoles (**dN^TP^** and **dN^TNO2^**) in good yields of 40–94% (Scheme 1, Table 1, entries 5–8). The phenyltriazole (in **dN^TP^**) was chosen as an electrochemically silent group, whereas the nitrophenyltriazole (in **dN^TNO2^**) should be reducible at an electrode due to the nitro group.

**Table 1:** Synthesis of the modified nucleosides and nucleotides

Entry	Starting compound	Reagent	Additives	Solvent	Product	Reaction time	Yield (%)
1	**dA^I^**	**1**	PdCl_2_(dppf), Cs_2_CO_3_	MeOH	**dA^A^**	2 h, 80 °C	58
2	**dC^I^**	**1**	PdCl_2_(dppf), Cs_2_CO_3_	MeOH	**dC^A^**	2 h, 80 °C	63
3	**dA^A^**	1. PO(OMe)_3_, POCl_3_, 0 °C; 2. (NHBu_3_)_2_H_2_P_2_O_7_, Bu_3_N, DMF, 0 °C; 3. TEAB			**dA^A^TP**	6 h, 0 °C	34
4	**dC^A^**	1. PO(OMe)_3_, POCl_3_, 0 °C; 2. (NHBu_3_)_2_H_2_P_2_O_7_, Bu_3_N, DMF, 0 °C; 3. TEAB			**dC^A^TP**	6 h, 0 °C	21
5	**dA^A^**	**2**	Sodium ascorbate, CuSO_4_·5H_2_O	*t*BuOH–H_2_O (1 : 1)	**dA^TP^**	12 h, rt	72
6	**dC^A^**	**2**	Sodium ascorbate, CuSO_4_·5H_2_O	*t*BuOH–H_2_O (1 : 1)	**dC^TP^**	12 h, rt	40
7	**dA^A^**	**3**	Sodium ascorbate, CuSO_4_·5H_2_O	*t*BuOH–H_2_O (1 : 1)	**dA^TNO2^**	12 h, rt	94
8	**dC^A^**	**3**	Sodium ascorbate, CuSO_4_·5H_2_O	*t*BuOH–H_2_O (1 : 1)	**dC^TNO2^**	12 h, rt	62
9	**dA^TP^**	1. PO(OMe)_3_, POCl_3_, 0 °C; 2. (NHBu_3_)_2_H_2_P_2_O_7_, Bu_3_N, DMF, 0 °C; 3. TEAB			**dA^TP^TP**	18 h, 0 °C	13
10	**dC^TP^**	1. PO(OMe)_3_, POCl_3_, 0 °C; 2. (NHBu_3_)_2_H_2_P_2_O_7_, Bu_3_N, DMF, 0 °C; 3. TEAB			**dC^TP^TP**	18 h, 0 °C	52
11	**dA^TNO2^**	1. PO(OMe)_3_, POCl_3_, 0 °C; 2. (NHBu_3_)_2_H_2_P_2_O_7_, Bu_3_N, DMF, 0 °C; 3. TEAB			**dA^TNO2^TP**	18 h, 0 °C	18
12	**dC^TNO2^**	1. PO(OMe)_3_, POCl_3_, 0 °C; 2. (NHBu_3_)_2_H_2_P_2_O_7_, Bu_3_N, DMF, 0 °C; 3. TEAB			**dC^TNO2^TP**	18 h, 0 °C	18

For the preparation of **dN^A^TPs**, we have applied a triphosphorylation^16^ of the corresponding nucleosides (**dN^A^**). Treatment of **dC^A^** or **dA^A^** with POCl_3_ in PO(OMe)_3_ followed by the addition of (NHBu_3_)_2_H_2_P_2_O_7_ and Bu_3_N, and then treatment with TEAB (Scheme 1) gave the desired **dN^A^TPs** (Table 1, entries 3 and 4) in 21 and 34% yield after isolation by RP HPLC. Triazole-modified triphosphates **dN^TP^TP** and **dN^TNO2^TP** were prepared by analogous triphosphorylation of modified nucleosides **dN^TP^** and **dN^TNO2^** (Scheme 1, Table 1, entries 9–12) in 13–52% yield.

### Electrochemistry of modified dNTPs

**Fig. 1:**
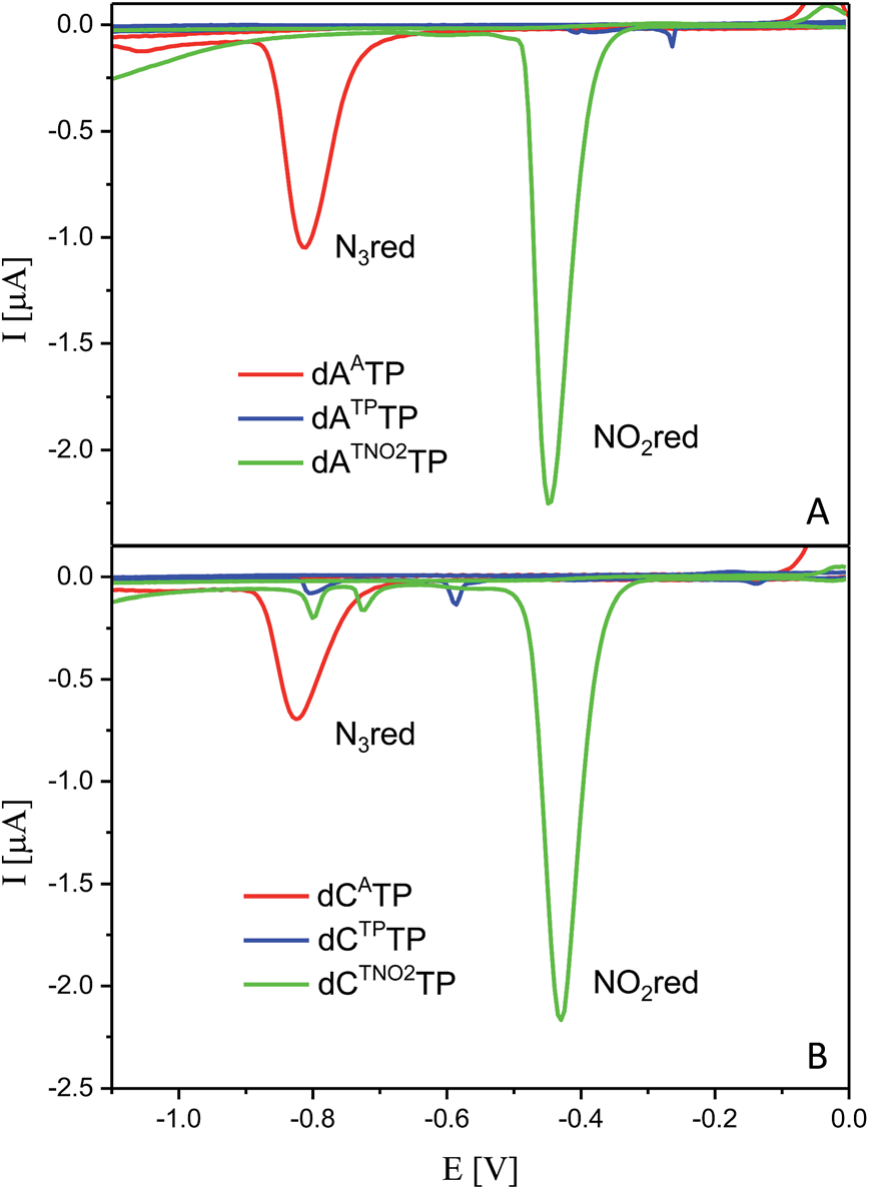
CV responses of **dN^A^TPs**, **dN^TP^TPs** and **dN^TNO^TPs** at HMDE.

All six modified dNTPs **dA^A^TP**, **dC^A^TP**, **dA^TP^TP**, **dC^TP^TP**, **dA^TNO2^TP** and **dC^TNO2^TP** were subjected to an electrochemical study using cyclic voltammetry at a hanging mercury drop electrode (HMDE; Fig. 1). The azidophenyl modified nucleotides **dA^A^TP** and **dC^A^TP** exerted a strong reduction peak at –0.9 V (peak Nred3), whereas the phenyltriazole derivatives **dA^TP^TP** and **dC^TP^TP** did not produce any redox signals from the label. The nitrophenyltriazole derivatives **dA^TNO2^TP** and **dC^TNO2^TP** gave a strong reduction peak at –0.4 V, due to the reduction of the nitro group (peak NOred2). Since the azidophenyl derivatives are easily transformed to both types of triazole by CuAAC reactions with alkynes, the click reaction with phenylacetylene can be used for silencing of the redox signal of the azido group whereas the click reaction with nitrophenylacetylene can be used for transformation of one redox label (azido) into another (nitro), exerting a different redox potential (*vide infra* for analytical applications of this finding).

### Enzymatic synthesis of modified DNA

The next goal was the polymerase-catalyzed synthesis of DNA bearing azidophenyl labels and the study of their conversion to (nitro)phenyltriazole groups by CuAAC of the azidophenyl modified DNA with acetylenes **2** or **3**. For comparison, the direct incorporation of triazole-modified nucleotides using **dN^TP^TP**s and **dN^TNO2^TP**s as substrates, leading to the same triazole-modified DNA molecules, was also tried.

**Fig. 2:**
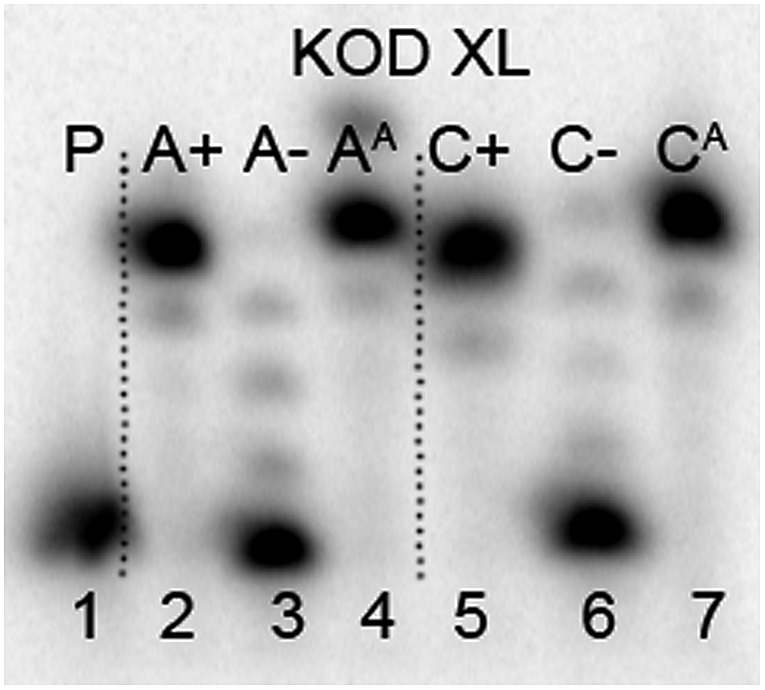
PAGE analysis of PEX single-incorporations into 19 nt DNA using **dN^A^TP**, temp^A^ and temp^C^ template and KOD XL polymerase.

The enzymatic incorporations of the azidophenyl modified nucleotides were studied using a primer extension (PEX) process, with **dN^A^TP**s as the substrates together with a 19 nt template, a radiolabeled 15 nt primer and a DNA polymerase, KOD XL (Fig. 2) or Pwo (Fig. S1 in the ESI†), and the products were analyzed by sequencing polyacrylamide gel electrophoresis (PAGE). In all cases fully extended products were obtained.

Then we performed a simple kinetics study to explore the efficiency of the PEX with the modified **dN^A^TP**s in comparison to natural dNTPs. The rates of the PEX using Pwo DNA polymerase with temp^C^ (for C, without natural dGTP), temp^Aterm^ (for A) and prim^rnd^ were compared (see Table 2). The reaction mixtures were incubated for the time intervals indicated, and then the reactions were stopped by the addition of PAGE loading buffer and immediate heating. The incorporation of the natural nucleotides was finished in 1–2 minutes whereas the PEX with **dN^A^TP**s took *ca.* 1–10 minutes to complete (Fig. S3 and S4 in the ESI†), but the synthesis was always completed within 10 minutes.

**Table 2:** Primers and templates used for PEX experiments^*a*^

Sequences	
Prim^rnd^	5′-CATGGGCGGCATGGG-3′
Temp^rnd16^	5′-CTAGCATGAGCTCAGT*CCCATGCCGCCCATG*-3′
Temp^A^	5′-CCCTCCCATGCCGCCCATG-3′
Temp^Aterm^	5′-TCCCATGCCGCCCATG-3′
Temp^C^	5′-CCCGCCCATGCCGCCCATG-3′
Prim^p53_15^	5′-GAATTCGATATCAAG-3′
Temp^p53_2CON_4^	5′-TACCTTATCCATAATAGACATGTCTAGACATGTCTCTTGATATCGAATTC-3′
Temp^p53_1a2G^	5′-TAGGTTATGGATAATAAACATGTCTAGGCATGTCTCTTGATATCGAATTC-3′
ON^p53_2CON_4^	5′-GAATTCGATATCAAG***A*** *G* ***A*** *C* ***A*** *TGTCT* ***A*** *G* ***A*** *C* ***A*** *TGTCT* **A**TT**A**TGG**A**T**AA**GGT**A**-3′
ON^p53_1a2G^	5′-GAATTCGATATCAAG***A*** *G* ***A*** *C* ***A*** *TGCCT* ***A*** *G* ***A*** *C* ***A*** *TGTTT* **A**TT**A**TCC**A**T**AA**CCT**A**-3′

^*a*^p53 recognition sequences are in italics and nucleotides bearing modification are in bold and underlined.

**Fig. 3:**
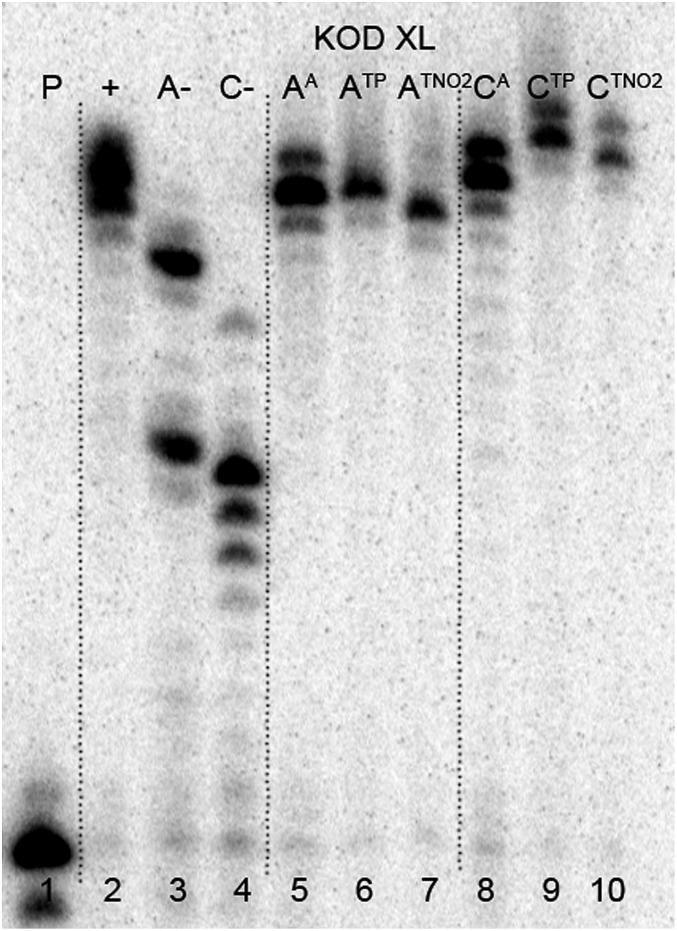
PAGE analysis of PEX incorporations into 31 nt DNA using **dN^A^TP**, template temp^rnd16^ and KOD XL polymerase, followed by click reaction with 1-ethynyl-4-nitrobenzene and phenylacetylene.

The multiple incorporations of **dN^A^** nucleotides into random sequences were tested using a 31 nt template in the presence of KOD XL (Fig. 3), Pwo (Fig. S5 in the ESI†) or Vent (*exo*-) (Fig. S6 in the ESI†). PEX reactions with both modified **dN^A^TP**s in the presence of any of these polymerases were successful, giving full-length products in PAGE analyses (Fig. 3, lane 5 and 8).

In order to study the CuAAC click modification, the azidophenyl-labelled dsDNA was synthesized on a larger scale (increased 10 times) and the PEX products were purified using a QIAquick Nucleotide Removal Kit to remove the dNTPs. Then the Cu(i)-catalyzed CuAAC reaction of the azidophenyl-labelled dsDNA with phenylacetylene or 1-ethynyl-4-nitrobenzene was performed under standard conditions in the presence of CuBr, TBTA (tris[(1-benzyl-1*H*-1,2,3-triazol-4-yl)methyl]amine) ligand and sodium ascorbate, in aqueous *t*BuOH–DMSO (1 : 3) at 37 °C for 2 hours. The products were purified once again and analyzed by PAGE (Fig. 3, lane 6, 7, 9, 10) to show no apparent degradation of DNA and MALDI-TOF (see ESI, Fig. S21–S24†) to confirm the conversion.

**Fig. 4:**
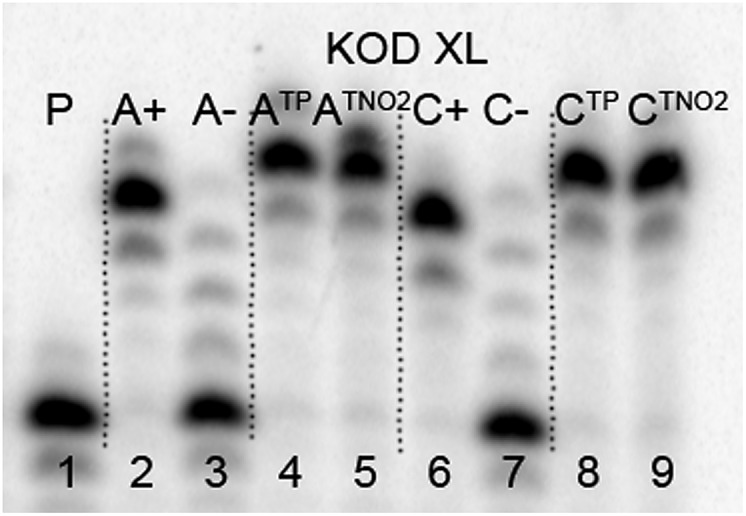
PAGE analysis of PEX single-incorporations into 19 nt DNA using **dN^TNO2^TP** or **dN^TP^TP**, temp^A^ or temp^C^ template and KOD XL polymerase.

**Fig. 5:**
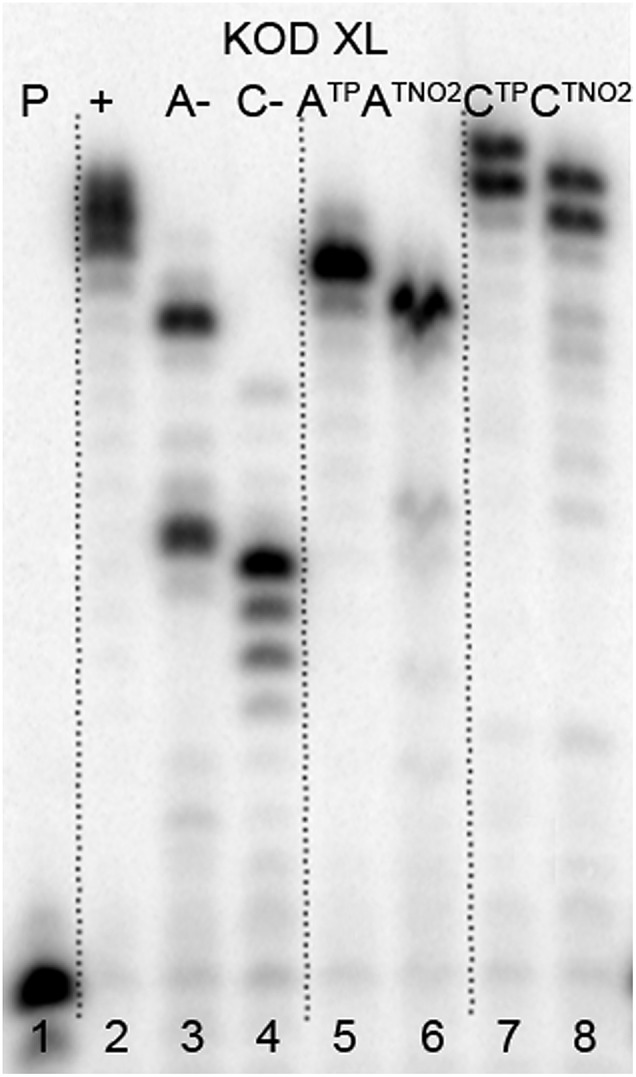
PAGE analysis of PEX reactions with **dN^TP^TP** or **dN^TNO2^TP** using template temp^rnd16^ and KOD XL polymerase leading to 31 nt DNA.

The direct PEX incorporation of **dN^TP^** and **dN^TNO2^** nucleotides into DNA was also studied using either a 19 nt or 31 nt template, KOD XL (Fig. 4 and 5) or Pwo polymerase and **dN^TP^TP** or **dN^TNO2^TP** as the substrate (Fig. S2 in ESI†). In single incorporations all **dN^Tx^** nucleotides were successfully incorporated into DNA (Fig. 4, lane 4, 5, 8, 9). In multiple incorporations, **dC^Tx^TP**s gave fully extended products (Fig. 5, lane 5, 7, 8), whereas for the PEX using **dA^TNO2^TP** (Fig. 5, lane 6) the product stopped in the same line as the negative control A-, probably due to steric hindrance of the bulky nitrophenyltriazolylphenyl group.

### Electrochemistry of the modified DNA

**Fig. 6:**
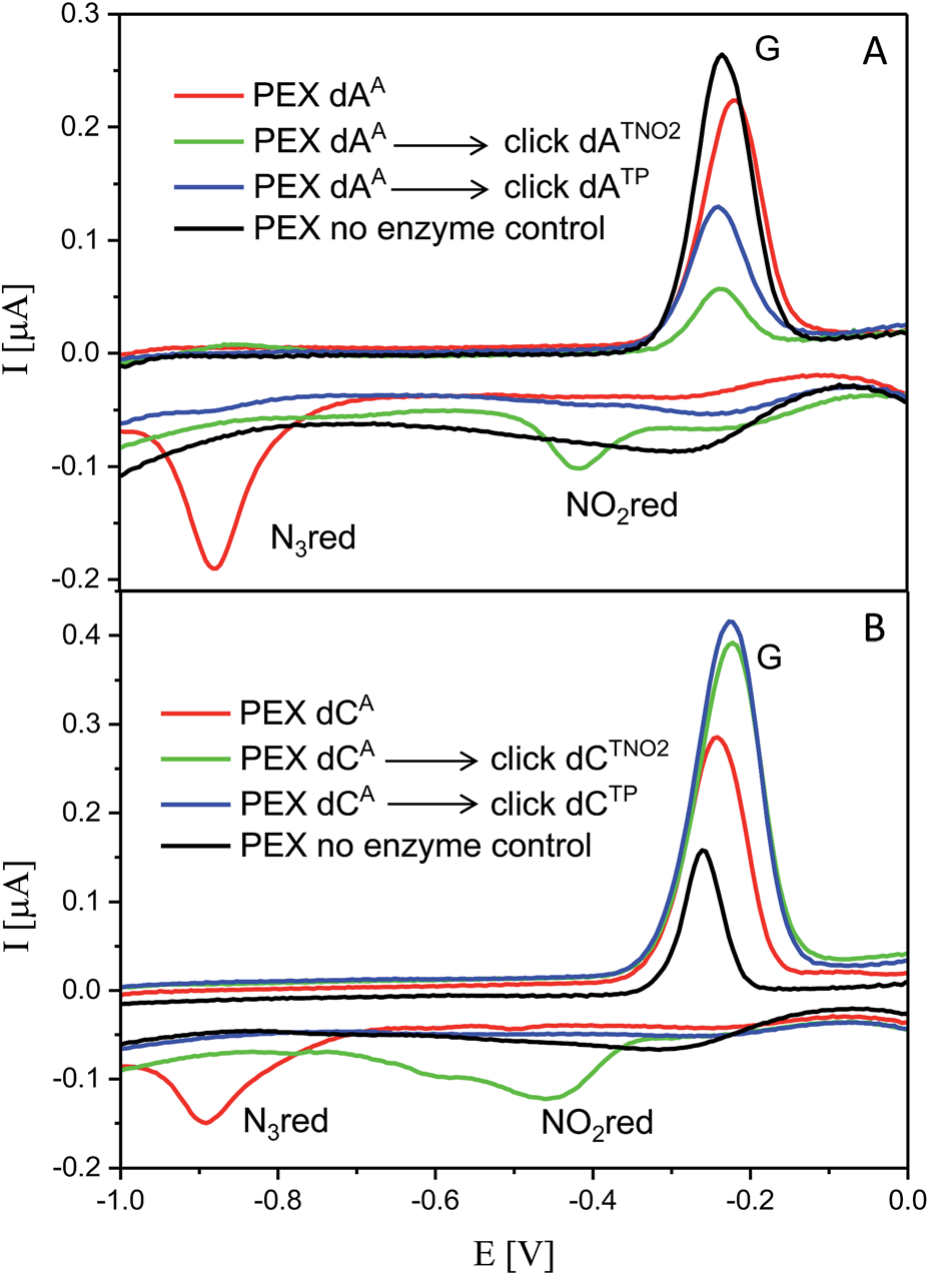
CV responses at a HMDE of PEX products synthesized with a temp^rnd16^ template and dNTP mixes containing a **dN^A^TP** conjugate (as specified in the legend) complemented with three respective unmodified dNTPs and PEX products after click reaction with (nitro)phenyltriazole. Peak G corresponds to oxidation of a reduction product of guanine generated at the electrode.^1*a*^ For full CV scans and other details see Fig. S14–S15† and Experimental section.

The voltammetric properties of modified DNA (the PEX products) containing **A^A^** or **C^A^** were studied by using cyclic voltammetry (CV) at a HMDE. Similarly to the electrochemistry of modified **dN^x^TP**s, cyclic voltammograms of PEX products prepared with the temp^rnd16^ template containing azidophenyl-modified nucleobases (**A^A^** or **C^A^**) show an irreversible cathodic peak at around –0.9 V due to reduction of the azido group (peak Nred3, Fig. 6). A detailed study of the electrode reaction mechanism will be published elsewhere. In a negative control experiment of PEX reactions with no polymerase added to the mixture, no signal corresponding to the azido group was detected, which excludes the presence of unremoved **dN^A^TPs** in the mixture. PEX products containing azido groups **A^A^** or **C^A^** were transformed by click reaction to PEX products containing phenyltriazole groups **A^TP^** or **C^TP^**, with no redox signal from the label being observed (blue curves). On the other hand, the CuAAC click reaction of **N^A^**-modified DNA with nitrophenylacetylene provides DNA products containing nitrophenyltriazole groups **A^TNO2^** and **C^TNO2^**, which produce the irreversible cathodic peak at around –0.4 V due to the reduction of the nitro group (peak NOred2, Fig. 6).

**Fig. 7:**
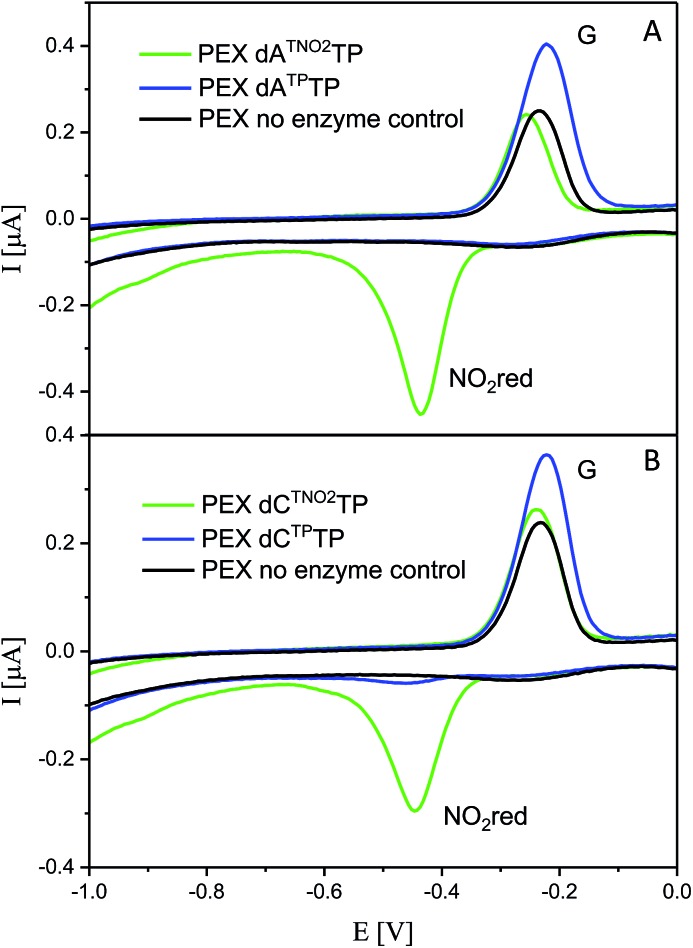
CV responses at a HMDE of PEX products synthesized with a temp^rnd16^ template and dNTP mixes containing a **dN^Tx^TP** conjugate (as specified in the legend) complemented with three respective unmodified dNTPs. For full CV scans and other details see Fig. S16–S17† and Experimental section.

For comparison, we also prepared PEX products containing the **N^TP^** and **N^TNO2^** modifications by polymerase-catalyzed incorporation of the corresponding triazole-modified **dN^Tx^TP**s. Voltammetric responses of the PEX products were again measured at a HMDE and Fig. 7 confirms the irreversible cathodic peak NOred2 at around –0.4 V corresponds to PEX products containing the nitrophenyltriazole label whereas PEX products containing the phenyltriazole label did not produce any redox signals from the label.

### Application of the click transformations of the redox labels in detection of DNA–protein interactions

**Scheme 2:**
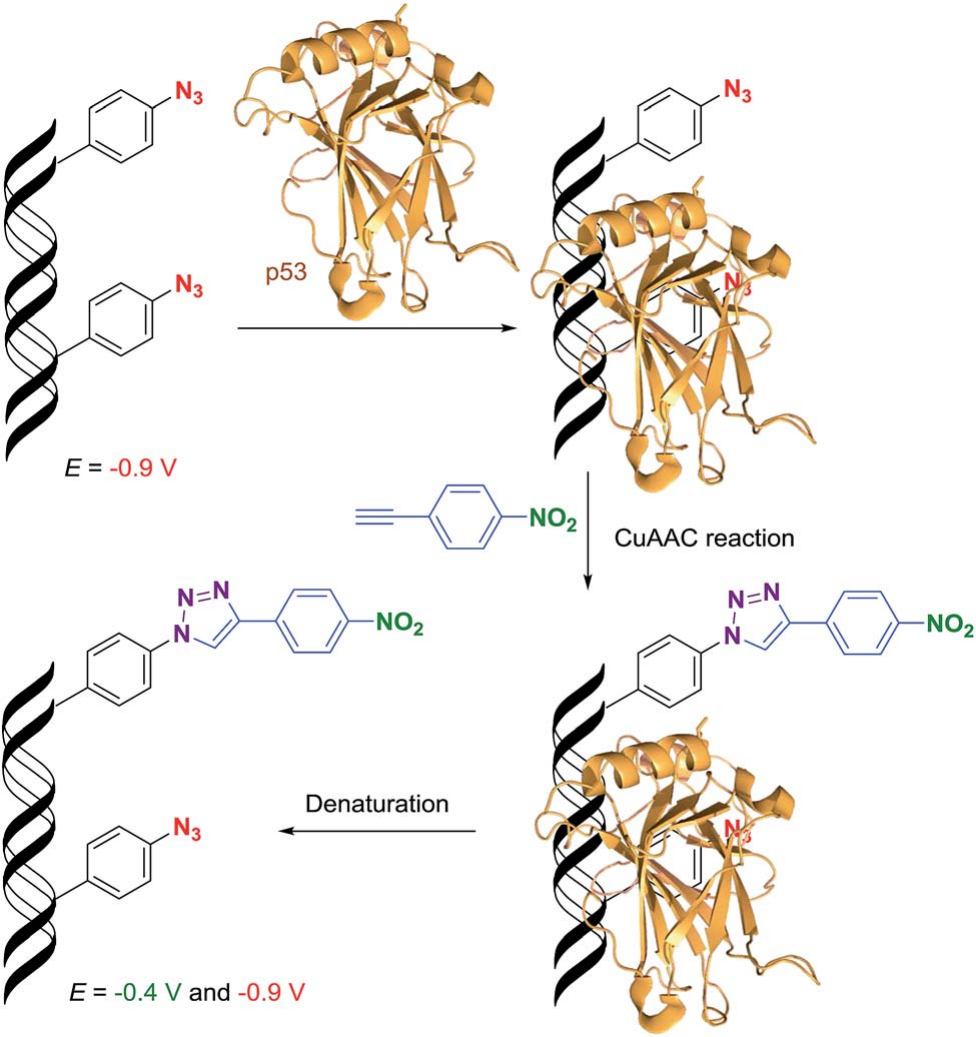
The principle of electrochemical detection of protein–DNA interactions.

DNA–protein interactions are of paramount importance in DNA recombination, transcription, methylation and repair. The current techniques available for footprinting of these interactions are mostly based on specific DNA cleavage.^6,7^ Based on our encouraging results in the transformation of an azido redox label to nitrophenyltriazole, we envisaged that this approach could be used as a new method for the detection of protein–DNA interactions (Scheme 2). We assumed that if we incorporate several azidophenyl-modifications into a DNA probe, incubate the probe with a protein and then perform the CuAAC click reaction with nitrophenylacetylene, only the freely accessible azido-groups (not shielded by the protein) should be transformed to nitrophenyltriazoles and the ratio of azido/nitro redox signals should indicate whether the protein was bound to the DNA and how large was the sequence of contact.

**Fig. 8:**
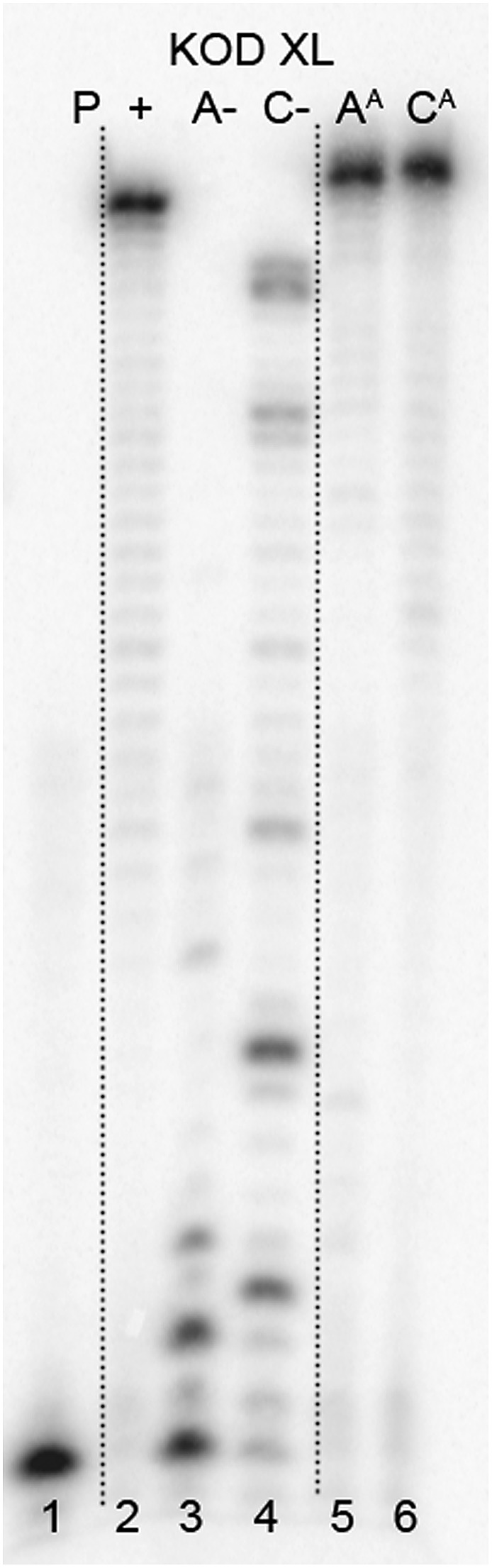
PAGE analysis of PEX reactions with **dN^A^TP** using template temp^p53-1a2G^ and KOD XL DNA polymerase, giving 50 nt DNA products.

To test this idea, we chose a GST-tagged DNA binding (core) domain of tumor-suppressor protein p53 (ref. 17 and 18) (p53CD_GST) as a biologically relevant example of a sequence-specific^19^ binder to DNA. We have previously shown that p53 retained binding to a specific DNA sequence containing vinylsulfonamide modifications in the major groove, which efficiently cross-linked with a cysteine of p53 through Michael addition.^20^ We prepared two different sequences of 50-bp DNA by PEX (using KOD XL polymerase and template temp^p53_1a2G^ or temp^p53_2CON_4^) in which 6 azido-groups are inside and 6 azido-groups are outside of the sequence specifically recognized by p53. Both azido-modified **dN^A^TP**s gave full length ON-products which were characterized by PAGE (Fig. 8, lane 5 and 6 and Fig. S7 in the ESI†).

**Fig. 9:**
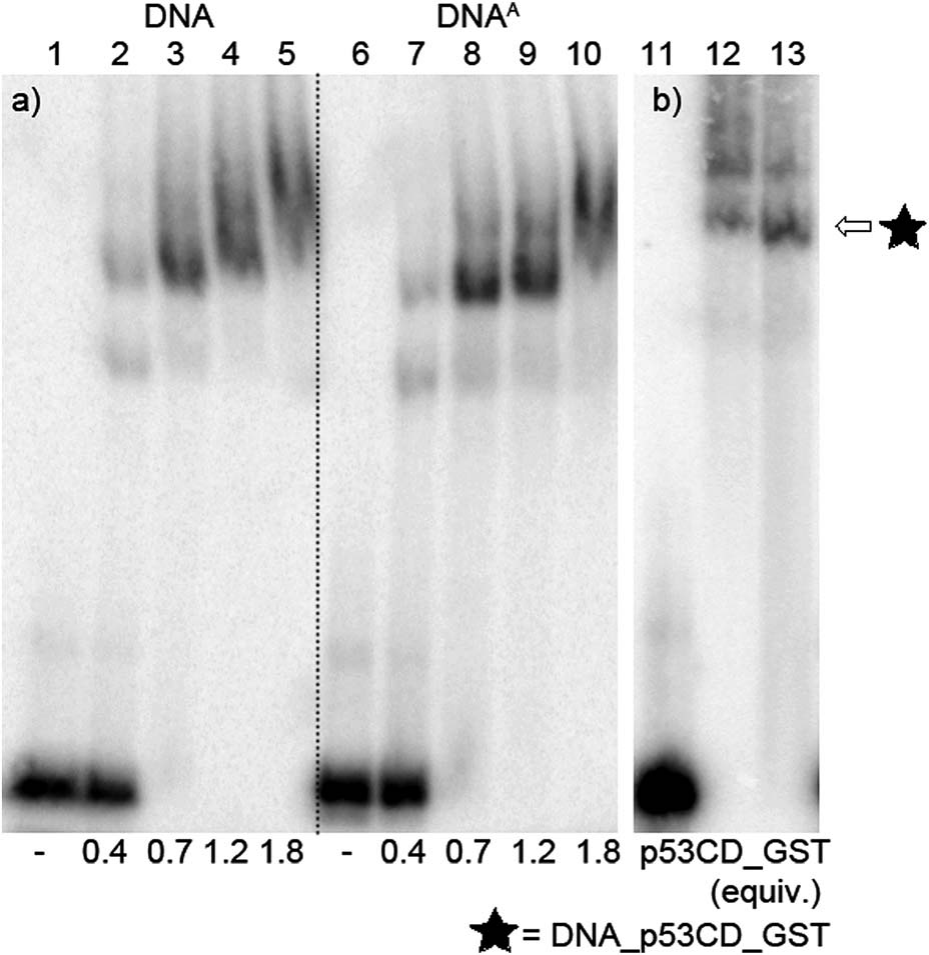
(a) Native PAGE analysis of the 50-mer DNA^1a2G^_p53CD_GST complex. Lane 1: natural DNA; 2 : 0.4 equiv.; 3 : 0.7 equiv.; 4 : 1.2 equiv.; 5 : 1.8 equiv. of protein p53CD_GST to DNA; lane 6: DNA^A^; 7 : 0.4 equiv.; 8 : 0.7 equiv.; 9 : 1.2 equiv.; 10 : 1.8 equiv. of protein p53CD_GST to DNA. (b) Native PAGE analysis of the stability of the DNA_p53CD_GST complex after click reaction of the DNA. Template^p53_1a2G^ : lane 11: DNA^A^; lane 12: protein–DNA complex; lane 13: protein–DNA complex, 0.5 mM 4-nitrophenylacetylene, 5 μM CuBr; 25 μM TBTA ligand, 65 μM Na ascorbate, 20 °C, 1 h.

After successful synthesis of the azido-modified DNA, it was incubated with different ratios of p53CD_GST protein to test its binding activity. The recognition of the binding sequence by p53CD_GST was monitored by 6% native PAGE (Fig. 9a, lane 1–10, and Fig. S8 in the ESI†). For further experiments we decided to use 1.2 equiv. of protein p53CD_GST (under these conditions the modified DNA was quantitatively bound by the protein, forming predominantly a 1 : 1 p53–DNA complex, Fig. 9). Then it was necessary to test the thermal stability of the DNA–protein complex (DNA_p53CD_GST complex), by incubating the created DNA_p53CD_GST complexes at the stated temperatures for 1 h. The DNA_p53CD_GST complex was found to be stable at 20 °C for 1 hour (Fig. S10 and S11,† lane 3). At higher temperatures, the binding of p53 to DNA is inefficient (Fig. S10 and S11,† lane 5).

Transition metals, such as nickel, copper, cobalt and zinc, in high concentrations may also cause the inhibition or disruption of DNA–protein binding.^21^ Therefore the stability of the DNA_p53CD_GST complex under different Cu^I^ concentrations was studied. DNA_p53CD_GST complexes were mixed with different concentrations of CuBr solution in the presence or absence of a TBTA ligand at 20 °C for 1 h. Relatively low concentrations of CuBr (10 μM) prevent the inhibitory effect due to copper from occurring on binding of p53 to DNA (Fig. S12 and S13,† lane 6 and 7). At higher concentrations of CuBr (20 μM), the binding of p53 to DNA is completely inhibited (Fig. S13 and S14,† lane 8 and 9). For the next experiments we decided to use 5 μM CuBr. In the last control experiment, we tested the stability of the DNA_p53CD_GST complex during the CuAAC click reaction. The DNA_p53CD_GST complex was mixed with 0.5 mM 4-nitrophenylacetylene, 5 μM CuBr, 25 μM TBTA ligand, 65 μM sodium ascorbate at 20 °C for 1 h. Fig. 9b, lane 13 (and Fig. S9,† lane 3 in ESI) shows that the DNA–protein complex was stable during the reaction under these conditions.

**Fig. 10:**
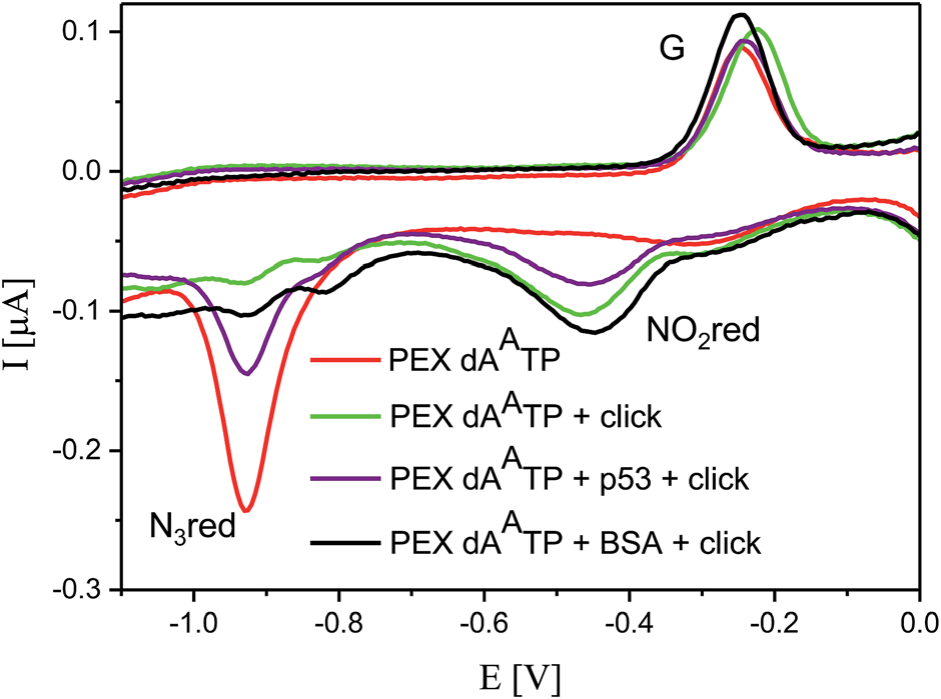
CV responses at a HMDE of PEX products synthesized with temp^p53_1a2G^ template and a **dA^A^TP** conjugate complemented with three respective unmodified dNTPs (red curve); PEX products after click reaction with nitrophenylacetylene (green curve); DNA–p53 complex after click reaction followed by denaturation (violet curve); and the control with BSA (black curve). For full CV scans and other details see Fig. S18† and Experimental section.

Then we proceeded to the experiments involving electrochemical detection of DNA–protein interactions. The 50-bp dsDNA containing 12 azidophenyl groups was prepared by PEX with temp^p53-1a2G^ template, and the CV showed the characteristic peak for N_3_ reduction at –0.9 V (Fig. 10, red curve). This **A^A^**-linked DNA was then reacted with nitrophenylacetylene (**3**) under the previously optimized conditions (suitable for survival of DNA–protein complexes), in the presence of CuBr, TBTA and sodium ascorbate and in the absence of p53CD_GST, to show that full conversion of all the azido-groups to nitrophenyltriazoles occurred, which was confirmed by the disappearance of the signal at –0.9 V and appearance of a new signal at –0.4 V due to reduction of the NO_2_ group (Fig. 10, green curve). In a further experiment, the **A^A^**-linked DNA was incubated with 1.2 equiv. of p53CD_GST (for 45 min on ice) to form a complex and then treated with nitrophenylacetylene (**3**) under the above mentioned conditions, followed by denaturation. The voltammetric analysis of the product (Fig. 10, violet curve) revealed a *ca.* 50% decrease in intensity of the peak Nred3 for reduction of azido-group and an increase of the peak NOred2 corresponding to the reduction of the nitro group (to *ca.* 50% intensity compared to the reaction in the absence of p53). This confirms that only those azido-groups which are not shielded by protein binding can undergo the click transformation to nitrophenyltriazole, whereas the N_3_ groups covered by the protein remain intact. As a control, we performed the CuAAC reaction of **A^A^**-linked DNA in the presence of bovine serum albumin (BSA), which does not bind DNA, and obtained the same results as for the experiment conducted in the absence of any protein (Fig. 10, black curve, all azido groups were converted to nitrophenyltriazoles). Almost identical results were obtained with **A^A^**-modified DNA synthesized using the temp^p53-2CON4^ template (Fig. S19 and S20†).

## Conclusions

We have designed and prepared nucleosides and dNTPs bearing azidophenyl groups and developed a polymerase mediated incorporation of azido-modified nucleotides into DNA by primer extension using these **dN^A^TP**s as substrates. Both nucleotides and DNA bearing the azidophenyl modifications exert a strong reduction peak around –0.9 V under voltammetry. The azido-group readily undergoes a CuAAC click reaction with phenyl- or nitrophenylacetylene under conditions compatible to working with dsDNA and DNA–protein complexes. The phenyltriazole-modified dNTPs do not produce any reduction signals in the voltammetric scans, and so the transformation of the azido-modification to a triazole results in silencing of the voltammetric signal. On the other hand, the nitrophenyltriazoles (resulting from CuAAC transformation of the azides with nitrophenylacetylene) give a strong reduction signal at –0.4 V. Thus the azidophenyl modified nucleotides are useful redox labels for DNA, which can be easily either silenced or transformed to a different redox label with a distinctly different redox potential. This transformation was utilized in the detection and mapping of DNA–protein interactions. When an **A^A^**-containing DNA is incubated with a protein, binding to a part of its sequence, and then undergoes the CuAAC reaction with nitrophenylacetylene, only the free azido-groups not covered by the protein would react whereas the azides in close contact with the protein remain intact. The electrochemical readout is used for the analysis of the outcome and one can not only distinguish whether or not the protein formed a stable complex with the DNA but also directly deduce the length of the sequence in contact with the particular protein (from the ratio of transformed *versus* not transformed labels resulting from analysis of changes in intensity of the corresponding redox peaks in voltammetry). Apparently, by the proper design of a series of differently labelled probes, one could even determine the binding sequences for DNA–protein footprinting. Moreover, the method has potential for being conducted in parallel and applied to high-throughput screening of ligands that interfere with protein–DNA binding. Since the azido-group can be photolyzed to reactive nitrenes,^22^ the azido-modified DNA could also be applied to cross-linking with proteins. Studies along these lines and toward practical applications of this approach will continue in our laboratories.

## Experimental section

NMR spectra were measured at 500 MHz for ^1^H and 125.7 MHz for ^13^C, or at 600 MHz for ^1^H and 150.9 MHz for ^13^C when using D_2_O (reference to dioxane as internal standard, *δ*_H_ = 3.75 ppm, *δ*_C_ = 67.19 ppm) or [D_6_]DMSO (reference to the residual solvent signal) as the solvent. Chemical shifts are given in ppm (*δ* scale) and coupling constants (*J*) in Hz. Complete assignment of all NMR signals was achieved using a combination of H,H-COSY, H,C-HSQC, and H,C-HMBC experiments. Mass spectra were measured with a LCQ classic (Thermo-Finnigan) spectrometer using ESI or a Q-Tof Micro spectrometer (Waters, ESI source, internal calibration with lockspray). Semipreparative separation of nucleoside triphosphates was performed using HPLC on a column packed with 10 μm C18 reversed phase (Phenomenex, Luna C18 (2)). IR spectra were measured using the ATR technique or by using KBr discs. High-resolution mass spectra were measured using an ESI ionization technique. Mass spectra of the functionalized DNA were measured using Maldi-TOF, Reflex IV (Bruker) with a nitrogen laser. Melting points were determined on a Kofler block. Known starting compounds were prepared by literature procedures (compound potassium 4-azidophenyltrifluoroborate^14^).

### Method A: Suzuki–Miyaura cross-coupling reaction

#### **dC^A^**, **dA^A^**

To a glass vial containing a stirrer bar was added halogenated nucleosides **dN^I^** (0.1 g, 0.2 mmol), potassium 4-azidophenyltrifluoroborate (95 mg, 0.4 mmol, 1.5 equiv.), Cs_2_CO_3_ (0.27 g, 0.8 mmol, 3 equiv.) and PdCl_2_(dppf) (21 mg, 0.02 mmol, 10 mol%). The vial was sealed with a septum and methanol (5 mL) was added *via* syringe. The reaction was heated in an oil bath at 80 °C for 2 h until complete consumption of the starting material occurred (the reaction was monitored by TLC), and then the reaction mixture was cooled to rt. The solvent was evaporated *in vacuo*. The products were purified by silica gel column chromatography using chloroform–methanol (0–10%) as the eluent.

### Method B: triphosphorylation – synthesis of the modified nucleoside triphosphates

#### **dC^A^TP**, **dA^A^TP**

Dry trimethyl phosphate (0.11 mL) was added to an argon-purged flask containing a nucleoside analogue **dN^A^** (0.06 mmol, 1 equiv.) which was cooled to 0 °C on ice, followed by the addition of POCl_3_ (7 μL, 0.07 mmol, 1.2 equiv.). After 4 h, a solution of (NHBu_3_)_2_H_2_P_2_O_7_ (180 mg, 0.3 mmol, 5 equiv.) and Bu_3_N (0.06 mL, 0.3 mmol, 4.2 equiv.) in dry DMF (0.5 mL) was added to the reaction mixture and the mixture was stirred for another 1.5 h and then quenched using 2 M TEAB buffer (1 mL). The product was isolated from the crude reaction mixture using HPLC on a C18 column with the use of a linear gradient of 0.1 M TEAB (triethylammonium bicarbonate) in H_2_O to 0.1 M TEAB in H_2_O–MeOH (1 : 1) as the eluent. Several co-distillations with water and conversion to the sodium salt form (Dowex 50WX8 in Na^+^ cycle) followed by freeze-drying from water gave the solid product.

### Method C: general procedure for the CuAAC reactions^15^

#### **dC^TP^**, **dA^TP^**, **dC^TNO2^**, **dA^TNO2^**

Azido-modified nucleoside **dN^A^** (0.1 mmol), sodium ascorbate (12 mg, 0.06 mmol, 0.4 equiv.) and CuSO_4_·5H_2_O (3 mg, 0.01 mmol, 0.08 equiv.) were suspended in 5 mL of H_2_O–*t*BuOH (3 : 1). The appropriate alkyne (2 equiv.) was subsequently added, and the mixture was stirred overnight at room temperature. The 1,4-disubstituted 1,2,3-triazole derivatives (generally) precipitated from this reaction medium and were isolated by filtration with water.

### Method D: triphosphorylation – synthesis of modified nucleoside triphosphates

#### **dC^TP^TP**, **dA^TP^TP**, **dC^TNO2^TP**, **dA^TNO2^TP**

Dry trimethyl phosphate (0.11 mL) was added to an argon-purged flask containing a nucleoside analogue **dN^Tx^** (0.04 mmol, 1 equiv.) which was cooled to 0 °C on ice, followed by the addition of POCl_3_ (4 μL, 0.04 mmol, 1.2 equiv.). After 16 h, a solution of (NHBu_3_)_2_H_2_P_2_O_7_ (100 mg, 0.2 mmol, 5 equiv.) and Bu_3_N (0.04 mL, 0.15 mmol, 4.2 equiv.) in dry DMF (0.5 mL) was added to the reaction mixture and the mixture was stirred for another 1.5 h and then quenched using 2 M TEAB buffer (1 mL). The product was isolated from the crude reaction mixture using HPLC on a C18 column with the use of a linear gradient of 0.1 M TEAB (triethylammonium bicarbonate) in H_2_O to 0.1 M TEAB in H_2_O–MeOH (1 : 1) as the eluent. Several co-distillations with water and conversion to the sodium salt form (Dowex 50WX8 in Na^+^ cycle) followed by freeze-drying from water gave the solid product.

#### 5-(4-Azidophenyl)-2′-deoxycytidine (**dC^A^**)

Compound **dC^A^** was prepared from **dC^I^** according to the general procedure (Method A). The product was isolated as a brown solid (61 mg, 63%); m.p. 145 °C. ^1^H NMR (499.8 MHz, DMSO-d_6_): 2.07 (ddd, 1H, *J*_gem_ = 13.3, *J*_2′b,1′_ = 7.0, *J*_2′b,3′_ = 6.1, H-2′b); 2.15 (ddd, 1H, *J*_gem_ = 13.3, *J*_2′a,1′_ = 6.1, *J*_2′a,3′_ = 3.6, H-2′a); 3.50, 3.56 (2 × ddd, 2 × 1H, *J*_gem_ = 11.8, *J*_5′,OH_ = 5.0, *J*_5′,4′_ = 3.6, H-5′); 3.77 (q, 1H, *J*_4′,3′_ = *J*_4′,5′_ = 3.6, H-4′); 4.21 (m, 1H, *J*_3′,2′_ = 6.1, 3.6, *J*_3′,OH_ = 4.3, *J*_3′,4′_ = 3.6, H-3′); 4.95 (t, 1H, *J*_OH,5′_ = 5.0, OH-5′); 5.19 (d, 1H, *J*_OH,3′_ = 4.3, OH-3′); 6.19 (dd, 1H, *J*_1′,2′_ = 6.7, 6.2, H-1′); 6.39 (bs, 1H, NH_a_H_b_); 7.17 (m, 2H, H-*m*-phenylene); 7.39 (m, 2H, H-*o*-phenylene); 7.39 (bs, 1H, NH_a_H_b_); 7.86 (s, 1H, H-6); ^13^C NMR (125.7 MHz, DMSO-d_6_): 40.79 (CH_2_-2′); 61.18 (CH_2_-5′); 70.31 (CH-3′); 85.26 (CH-1′); 87.43 (CH-4′); 107.04 (C-5); 119.81 (CH-*m*-phenylene); 130.75 (CH-*o*-phenylene); 131.02 (C-*i*-phenylene); 138.74 (C-*p*-phenylene); 140.32 (CH-6); 154.58 (C-2); 163.53 (C-4); *ν*(KBr) cm^–1^: 3416, 3062, 2121, 2097, 1644, 1608, 1509, 1415, 1294, 1096, 1052, 787; MS (ESI+): *m*/*z* (%): 345.2 (75) [M + H], 367.2 (25) [M + Na]. HRMS (ESI+): calcd for C_15_H_17_N_6_O_4_: 345.13058; found 345.13057.

#### 7-(4-Azidophenyl)-7-deaza-2′-deoxyadenosine (**dA^A^**)

Compound **dA^A^** was prepared from **dA^I^** according to the general procedure (Method A). The product was isolated as a yellow solid (56 mg, 58%); m.p. 96 °C. ^1^H NMR (499.8 MHz, DMSO-d_6_): 2.19 (ddd, 1H, *J*_gem_ = 13.1, *J*_2′b,1′_ = 5.9, *J*_2′b,3′_ = 2.7, H-2′b); 2.56 (ddd, 1H, *J*_gem_ = 13.1, *J*_2′a,1′_ = 8.3, *J*_2′a,3′_ = 5.9, H-2′a); 3.51 (ddd, 1H, *J*_gem_ = 11.8, *J*_5′b,OH_ = 6.0, *J*_5′b,4′_ = 4.3, H-5′b); 3.57 (ddd, 1H, *J*_gem_ = 11.8, *J*_5′a,OH_ = 5.2, *J*_5′a,4′_ = 4.7, H-5′a); 3.83 (ddd, 1H, *J*_4′,5′_ = 4.7, 4.3, *J*_4′,3′_ = 2.4, H-4′); 4.36 (m, 1H, *J*_3′,2′_ = 5.9, 2.7, *J*_3′,OH_ = 4.0, *J*_3′,4′_ = 2.4, H-3′); 5.06 (dd, 1H, *J*_OH,5′_ = 6.0, 5.2, OH-5′); 5.28 (d, 1H, *J*_OH,3′_ = 4.0, OH-3′); 6.17 (bs, 2H, NH_2_); 6.58 (dd, 1H, *J*_1′,2′_ = 8.3, 5.9, H-1′); 7.23 (m, 2H, H-*m*-phenylene); 7.50 (m, 2H, H-*o*-phenylene); 7.54 (s, 1H, H-6); 8.14 (s, 1H, H-2); ^13^C NMR (125.7 MHz, DMSO-d_6_): 39.86 (CH_2_-2′); 62.16 (CH_2_-5′); 71.24 (CH-3′); 83.12 (CH-1′); 87.56 (CH-4′); 100.47 (C-4a); 115.77 (C-5); 119.87 (CH-*m*-phenylene); 120.93 (CH-6); 130.17 (CH-*o*-phenylene); 131.55 (C-*i*-phenylene); 138.09 (C-*p*-phenylene); 150.70 (C-7a); 151.93 (CH-2); 157.50 (C-4); *ν*(KBr) cm^–1^: 3418, 3394, 2126, 2092, 1583, 1501, 1621, 1128, 1094, 1053, 841; MS (ESI+): *m*/*z* (%): 368.1 (100) [M + H], 390.1 (10) [M + Na]. HRMS (ESI+): calcd for C_17_H_18_N_7_O_3_: 368.14656; found 368.14648.

#### 5-(4-Azidophenyl)-2′-deoxycytidine 5′-*O*-triphosphate (**dC^A^TP**)

Compound **dC^A^TP** was prepared from **dC^A^** according to the general procedure (Method B). The product was isolated as a yellow solid (7 mg, 21%). ^1^H NMR (600.1 MHz, D_2_O, ref(dioxane) = 3.75 ppm): 2.36 (ddd, 1H, *J*_gem_ = 14.2, *J*_2′b,1′_ = 7.3, *J*_2′b,3′_ = 6.4, H-2′b); 2.43 (ddd, 1H, *J*_gem_ = 14.2, *J*_2′a,1′_ = 6.3, *J*_2′a,3′_ = 3.6, H-2′a); 4.13 (m, 1H, H-5′b); 4.19 (m, 2H, H-4′,5′a); 4.63 (dt, 1H, *J*_3′,2′_ = 6.4, 3.6, *J*_3′,4′_ = 3.6, H-3′); 6.35 (dd, 1H, *J*_1′,2′_ = 7.3, 6.3, H-1′); 7.21 (m, 2H, H-*m*-phenylene); 7.45 (m, 2H, H-*o*-phenylene); 7.77 (s, 1H, H-6); ^13^C NMR (150.9 MHz, D_2_O, ref(dioxane) = 69.3 ppm): 41.66 (CH_2_-2′); 67.86 (d, *J*_C,P_ = 5.8, CH_2_-5′); 73.15 (CH-3′); 88.26 (d, *J*_C,P_ = 8.8, CH-4′); 88.61 (CH-1′); 112.85 (C-5); 122.47 (CH-*m*-phenylene); 131.41 (C-*i*-phenylene); 133.67 (CH-*o*-phenylene); 142.47 (CH-6); 142.99 (C-*p*-phenylene); 159.87 (C-2); 167.48 (C-4); ^31^P{^1^H} NMR (202.3 MHz, D_2_O): –21.47 (dd, *J* = 20.1, 16.3, P_β_); –10.68 (d, *J* = 20.1, P_α_); –5.50 (d, *J* = 16.3, P_γ_); MS (ESI–): *m*/*z* (%): 503.3 (100) [M – H_2_PO_3_], 525.2 (75) [M – H – H_2_PO_3_ + Na], 583.3 (10) [M – H]. HRMS (ESI–): calcd for C_15_H_18_N_6_O_13_P_3_: 583.01502; found 583.01516.

#### 7-(4-Azidophenyl)-7-deaza-2′-deoxyadenosine 5′-*O*-triphosphate (**dA^A^TP**)

Compound **dA^A^TP** was prepared from **dA^A^** according to the general procedure (Method B). The product was isolated as a yellow solid (13 mg, 34%). ^1^H NMR (600.1 MHz, D_2_O, ref(dioxane) = 3.75 ppm): 2.48 (ddd, 1H, *J*_gem_ = 14.0, *J*_2′b,1′_ = 6.3, *J*_2′b,3′_ = 3.3, H-2′b); 2.75 (ddd, 1H, *J*_gem_ = 14.0, *J*_2′a,1′_ = 7.9, *J*_2′a,3′_ = 6.4, H-2′a); 4.12 (ddd, 1H, *J*_gem_ = 11.3, *J*_H,P_ = 5.5, *J*_5′b,4′_ = 4.2, H-5′b); 4.19 (ddd, 1H, *J*_gem_ = 11.3, *J*_H,P_ = 6.5, *J*_5′a,4′_ = 4.2, H-5′a); 4.24 (td, 1H, *J*_4′,5′_ = 4.2, *J*_4′,3′_ = 3.3, H-4′); 4.79 (dt, 1H, *J*_3′,2′_ = 6.4, 3.3, *J*_3′,4′_ = 3.3, H-3′); 6.70 (dd, 1H, *J*_1′,2′_ = 7.9, 6.3, H-1′); 7.21 (m, 2H, H-*m*-phenylene); 7.54 (s, 1H, H-6); 7.55 (m, 2H, H-*o*-phenylene); 8.18 (s, 1H, H-2); ^13^C NMR (150.9 MHz, D_2_O, ref(dioxane) = 69.3 ppm): 40.99 (CH_2_-2′); 68.16 (d, *J*_C,P_ = 5.5, CH_2_-5′); 73.81 (CH-3′); 85.46 (CH-1′); 87.90 (d, *J*_C,P_ = 9.0, CH-4′); 103.81 (C-4a); 120.34 (C-5); 122.26 (CH-*m*-phenylene); 122.92 (CH-6); 132.98 (C-*i*-phenylene); 133.00 (CH-*o*-phenylene); 141.81 (C-*p*-phenylene); 152.69 (C-7a); 154.23 (CH-2); 160.11 (C-4); ^31^P{^1^H} NMR (202.3 MHz, D_2_O): –21.44 (bdd, *J* = 19.6, 18.3, P_β_); –10.39 (d, *J* = 19.6, P_α_); –5.60 (bd, *J* = 18.3, P_γ_); MS (ESI–): *m*/*z* (%): 526.3 (100) [M – H_2_PO_3_], 548.3 (100) [M – H – H_2_PO_3_ + Na], 606.3 (5) [M – H], 628.3 (15) [M – 2H + Na]. HRMS (ESI–): calcd for C_17_H_19_N_7_O_12_P_3_: 606.03100; found 606.03103.

#### 5-[4-(4-Phenyl-1,2,3-triazol-1-yl)phenyl]-2′-deoxycytidine (**dC^TP^**)

Compound **dC^TP^** was prepared from **dC^A^** according to the general procedure (Method C). The product was isolated as a green solid (26 mg, 40%); m.p. > 300 °C. ^1^H NMR (499.8 MHz, DMSO-d_6_, *t* = 100 °C): 2.15 (ddd, 1H, *J*_gem_ = 13.3, *J*_2′b,3′_ = 6.7, *J*_2′b,1′_ = 6.4, H-2′b); 2.26 (ddd, 1H, *J*_gem_ = 13.3, *J*_2′a,1′_ = 6.4, *J*_2′a,3′_ = 4.0, H-2′a); 3.58, 3.64 (2 × bddd, 2 × 1H, *J*_gem_ = 12.0, *J*_5′,OH_ = 4.6, *J*_5′,4′_ = 3.8, H-5′); 3.84 (q, 1H, *J*_4′,3′_ = *J*_4′,5′_ = 3.8, H-4′); 4.28 (m, 1H, H-3′); 4.59 (bs, 1H, OH-5′); 4.85 (bs, 1H, OH-3′); 6.23 (*t*, 1H, *J*_1′,2′_ = 6.4, H-1′); 6.55 (bs, 2H, NH_2_); 7.39 (m, 1H, H-*p*-Ph); 7.50 (m, 2H, H-*m*-Ph); 7.59 (m, 2H, H-*o*-phenylene); 7.92 (s, 1H, H-6); 7.96 (m, 2H, H-*o*-Ph); 8.00 (m, 2H, H-*m*-phenylene); 9.12 (s, 1H, H-5-triazole); ^13^C NMR (125.7 MHz, DMSO-d_6_, *t* = 100 °C): 40.52 (CH_2_-2′); 61.01 (CH_2_-5′); 70.00 (CH-3′); 85.34 (CH-1′); 87.25 (CH-4′); 106.60 (C-5); 119.06 (CH-5-triazole); 120.30 (CH-*m*-phenylene); 125.24 (CH-*o*-Ph); 127.86 (CH-*p*-Ph); 128.56 (CH-*m*-Ph); 130.06 (CH-*o*-phenylene); 130.13 (C-*i*-Ph); 134.33 (C-*i*-phenylene); 135.73 (C-*p*-phenylene); 140.29 (CH-6); 147.22 (C-4-triazole); 154.01 (C-2); 163.20 (C-4); *ν*(KBr) cm^–1^: 3464, 3363, 1647, 1482, 1457, 1411, 1353, 1254, 1187, 1096, 1042, 1026, 956; MS (ESI+): *m*/*z* (%): 447.3 (10) [M + H], 469.3 (100) [M + Na]. HRMS (ESI+): calcd for C_23_H_22_N_6_O_4_Na: 469.15947; found 469.15920.

#### 7-[4-(4-Phenyl-1,2,3-triazol-1-yl)phenyl]-7-deaza-2′-deoxyadenosine (**dA^TP^**)

Compound **dA^TP^** was prepared from **dA^A^** according to the general procedure (Method C). The product was isolated as a yellow solid (34 mg, 72%); m.p. > 300 °C. ^1^H NMR (499.8 MHz, DMSO-d_6_, *t* = 100 °C): 2.30 (ddd, 1H, *J*_gem_ = 13.2, *J*_2′b,1′_ = 6.1, *J*_2′b,3′_ = 3.1, H-2′b); 2.60 (ddd, 1H, *J*_gem_ = 13.2, *J*_2′a,1′_ = 7.7, *J*_2′a,3′_ = 6.1, H-2′a); 3.60, 3.66 (2 × bdt, 2 × 1H, *J*_gem_ = 11.7, *J*_5′,OH_ = *J*_5′,4′_ = 4.5, H-5′); 3.90 (td, 1H, *J*_4′,5′_ = 4.5, *J*_4′,3′_ = 3.0, H-4′); 4.43 (bm, 1H, H-3′); 4.64 (bs, 1H, OH-5′); 4.92 (bs, 1H, OH-3′); 5.91 (bs, 2H, NH_2_); 6.52 (dd, 1H, *J*_1′,2′_ = 7.7, 6.1, H-1′); 7.39 (m, 1H, H-*p*-Ph); 7.51 (m, 2H, H-*m*-Ph); 7.58 (s, 1H, H-6); 7.72 (m, 2H, H-*o*-phenylene); 7.97 (m, 2H, H-*o*-Ph); 8.04 (m, 2H, H-*m*-phenylene); 8.20 (bs, 1H, H-2); 9.13 (bs, 1H, H-5-triazole); ^13^C NMR (125.7 MHz, DMSO-d_6_, *t* = 100 °C): 39.70 (CH_2_-2′); 61.86 (CH_2_-5′); 70.82 (CH-3′); 83.11 (CH-1′); 87.27 (CH-4′); 100.50 (C-4a); 114.99 (C-5); 119.13 (CH-5-triazole); 120.31 (CH-*m*-phenylene); 121.01 (CH-6); 125.23 (CH-*o*-Ph); 127.83 (CH-*p*-Ph); 128.56 (CH-*m*-Ph); 129.43 (CH-*o*-phenylene); 130.19 (C-*i*-Ph); 134.78 (C-*i*-phenylene); 135.21 (C-*p*-phenylene); 147.18 (C-4-triazole); 150.58 (C-7a); 151.50 (CH-2); 157.12 (C-4); *ν*(KBr) cm^–1^: 3437, 1657, 1626, 1536, 1483, 1461, 1095, 1048, 1027, 960, 798; MS (ESI+): *m*/*z* (%): 470.3 (90) [M + H], 492.3 (100) [M + Na]. HRMS (ESI+): calcd for C_25_H_24_N_7_O_3_: 470.19351; found 470.19342.

#### 5-[4-(4-(4-Nitrophenyl)-1,2,3-triazol-1-yl)phenyl]-2′-deoxycytidine (**dC^TNO2^**)

Compound **dC^TNO2^** was prepared from **dC^A^** according to the general procedure (Method C). The product was isolated as a red solid (30 mg, 62%); m.p. 230 °C. ^1^H NMR (600.1 MHz, DMSO-d_6_): 2.12 (bdt, 1H, *J*_gem_ = 13.3, *J*_2′b,3′_ = *J*_2′b,1′_ = 6.3, H-2′b); 2.19 (bddd, 1H, *J*_gem_ = 13.3, *J*_2′a,1′_ = 6.3, *J*_2′a,3′_ = 3.5, H-2′a); 3.53, 3.60 (2 × bdt, 2 × 1H, *J*_gem_ = 11.9, *J*_5′,OH_ = *J*_5′,4′_ = 4.5, H-5′); 3.80 (btd, 1H, *J*_4′,5′_ = 4.5, *J*_4′,3′_ = 3.2, H-4′); 4.25 (m, 1H, H-3′); 5.00 (bt, 1H, *J*_OH,5'_ = 4.5, OH-5′); 4.25 (bd, 1H, *J*_OH,5'_ = 3.6, OH-3′); 6.22 (*t*, 1H, *J*_1′,2′_ = 6.3, H-1′); 6.68 (bs, 2H, NH_2_); 7.61 (m, 2H, H-*o*-phenylene); 8.00 (s, 1H, H-6); 8.02 (m, 2H, H-*m*-phenylene); 8.23 (m, 2H, H-*o*-C_6_H_4_NO_2_); 8.40 (m, 2H, H-*m*-C_6_H_4_NO_2_); 9.62 (s, 1H, H-5-triazole); ^13^C NMR (150.9 MHz, DMSO-d_6_): 40.86 (CH_2_-2′); 61.13 (CH_2_-5′); 70.23 (CH-3′); 85.49 (CH-1′); 87.25 (CH-4′); 106.50 (C-5); 120.65 (CH-*m*-phenylene); 121.80 (CH-5-triazole); 124.73 (CH-*m*-C_6_H_4_NO_2_); 126.37 (CH-*o*-C_6_H_4_NO_2_); 130.72 (CH-*o*-phenylene); 134.89 (C-*i*-phenylene); 135.74 (C-*p*-phenylene); 136.78 (C-*i*-C_6_H_4_NO_2_); 140.82 (CH-6); 145.67 (C-4-triazole); 147.10 (C-*p*-C_6_H_4_NO_2_); 154.44 (C-2); 163.40 (C-4); (KBr) cm^–1^: 3454, 3320, 3206, 1657, 1643, 1606, 1519, 1481, 1411, 1341, 1289, 1180, 1108, 1033, 855, 786, 636, 526; MS (ESI+): *m*/*z* (%): 514.3 (100) [M + H]. HRMS (ESI+): calcd for C_23_H_21_N_7_O_6_Na: 514.14455; found 514.14447.

#### 7-[4-(4-(4-Nitrophenyl)-1,2,3-triazol-1-yl)phenyl]-7-deaza-2′-deoxyadenosine (**dA^TNO2^**)

Compound **dA^TNO2^** was prepared from **dA^A^** according to the general procedure (Method C). The product was isolated as a red solid (53 mg, 94%); m.p. 200 °C. ^1^H NMR (600.1 MHz, DMSO-d_6_): 2.24 (ddd, 1H, *J*_gem_ = 13.3, *J*_2′b,1′_ = 6.0, *J*_2′b,3′_ = 2.7, H-2′b); 2.59 (bddd, 1H, *J*_gem_ = 13.2, *J*_2′a,1′_ = 8.1, *J*_2′a,3′_ = 5.9, H-2′a); 3.53, 3.60 (2 × bdt, 2 × 1H, *J*_gem_ = 11.6, *J*_5′,OH_ = *J*_5′,4′_ = 4.5, H-5′); 3.86 (td, 1H, *J*_4′,5′_ = 4.5, *J*_4′,3′_ = 2.6, H-4′); 4.39 (m, 1H, H-3′); 5.04 (bt, 1H, *J*_OH,5′_ = 4.5, OH-5′); 5.30 (bd, 1H, *J*_OH,5′_ = 4.1, OH-3′); 6.48 (bs, 2H, NH_2_); 6.63 (dd, 1H, *J*_1′,2′_ = 8.1, 6.00, H-1′); 7.71 (s, 1H, H-6); 7.73 (m, 2H, H-*o*-phenylene); 8.07 (m, 2H, H-*m*-phenylene); 8.24 (m, 2H, H-*o*-C_6_H_4_NO_2_); 8.40 (m, 2H, H-*m*-C_6_H_4_NO_2_); 9.63 (s, 1H, H-5-triazole); ^13^C NMR (150.9 MHz, DMSO-d_6_): 39.56 (CH_2_-2′); 62.10 (CH_2_-5′); 71.18 (CH-3′); 83.17 (CH-1′); 87.62 (CH-4′); 101.14 (C-4a); 115.80 (C-5); 120.71 (CH-*m*-phenylene); 121.80 (CH-5-triazole); 124.17 (CH-6); 124.72 (CH-*m*-C_6_H_4_NO_2_); 126.32 (CH-*o*-C_6_H_4_NO_2_); 129.93 (CH-*o*-phenylene); 135.17 (C-*i*,*p*-phenylene); 136.80 (C-*i*-C_6_H_4_NO_2_); 145.61 (C-4-triazole); 147.06 (C-*p*-C_6_H_4_NO_2_); 150.52 (C-7a); 151.97 (CH-2); 157.34 (C-4); *ν*(KBr)/cm^–1^: 3440, 1657, 1625, 1607, 1589, 1536, 1517, 1481, 1466, 1408, 1342, 1289, 1107, 1067, 1038, 854, 796; MS (ESI+): *m*/*z* (%): 515.3 (100) [M + H]. HRMS (ESI+): calcd for C_25_H_23_N_8_O_5_: 515.17859; found 515.17839.

#### 5-[4-(4-Phenyl-1,2,3-triazol-1-yl)phenyl]-2′-deoxycytidine 5′-*O*-triphosphate (**dC^TP^TP**)

Compound **dC^TP^TP** was prepared from **dC^TP^** according to the general procedure (Method D). The product was isolated as a white solid (14 mg, 52%).^1^H NMR (600.1 MHz, D_2_O, ref(dioxane) = 3.75 ppm): 2.34 (ddd, 1H, *J*_gem_ = 14.1, *J*_2′b,1′_ = 7.1, *J*_2′b,3′_ = 6.5, H-2′b); 2.46 (ddd, 1H, *J*_gem_ = 14.1, *J*_2′a,1′_ = 6.3, *J*_2′a,3′_ = 3.6, H-2′a); 4.18–4.26 (bm, 3H, H-4′,5′); 4.63 (dt, 1H, *J*_3′,2′_ = 6.5, 3.6, *J*_3′,4′_ = 3.6, H-3′); 6.24 (dd, 1H, *J*_1′,2′_ = 7.1, 6.3, H-1′); 7.42 (m, 1H, H-*p*-Ph); 7.49 (m, 2H, H-*m*-Ph); 7.55 (m, 2H, H-*o*-phenylene); 7.77 (s, 1H, H-6); 7.79 (m, 2H, H-*m*-phenylene); 7.80 (m, 2H, H-*o*-Ph); 8.75 (s, 1H, H-5-triazole); ^13^C NMR (150.9 MHz, D_2_O, ref(dioxane) = 69.3 ppm): 42.09 (CH_2_-2′); 67.93 (d, *J*_C,P_ = 4.7, CH_2_-5′); 73.21 (CH-3′); 88.37 (d, *J*_C,P_ = 8.5, CH-4′); 89.03 (CH-1′); 111.70 (C-5); 122.96 (CH-5-triazole); 123.95 (CH-*m*-phenylene); 128.38 (CH-*o*-Ph); 131.60 (CH-*p*-Ph); 131.78 (C-*i*-Ph); 131.93 (CH-*m*-Ph); 133.18 (CH-*o*-phenylene); 135.91 (C-*i*-phenylene); 138.68 (C-*p*-phenylene); 142.82 (CH-6); 150.76 (C-4-triazole); 159.42 (C-2); 166.71 (C-4); ^31^P{^1^H} NMR (202.3 MHz, D_2_O): –21.56 (bm, P_β_); –10.69 (bm, P_α_); –6.88 (bm, P_γ_); MS (ESI–): *m*/*z* (%): 525.3 (60) [M – H_3_P_2_O_6_], 605.3 (100) [M – H_2_PO_3_], 627.2 (90) [M – H – H_2_PO_3_ + Na], 685.3 (5) [M – H]. HRMS (ESI–): calcd for C_23_H_24_N_6_O_13_P_3_: 685.06197; found 685.06211.

#### 7-[4-(4-Phenyl-1,2,3-triazol-1-yl)phenyl]-7-deaza-2′-deoxyadenosine 5′-*O*-triphosphate (**dA^TP^TP**)

Compound **dA^TP^TP** was prepared from **dA^TP^** according to the general procedure (Method D). The product was isolated as a white solid (4 mg, 13%). ^1^H NMR (600.1 MHz, D_2_O, ref(dioxane) = 3.75 ppm): 2.45 (ddd, 1H, *J*_gem_ = 13.8, *J*_2′b,1′_ = 6.1, *J*_2′b,3′_ = 3.0, H-2′b); 2.72 (bddd, 1H, *J*_gem_ = 13.8, *J*_2′a,1′_ = 7.8, *J*_2′a,3′_ = 6.4, H-2′a); 4.11, 4.17 (2 × bm, 2 × 1H, H-5′); 4.23 (bm, 1H, H-4′); 4.77 (bm, 1H, H-3′); 6.38 (bdd, 1H, *J*_1′,2′_ = 7.8, 6.1, H-1′); 7.32 (m, 2H, H-*m*-Ph); 7.35 (m, 1H, H-*p*-Ph); 7.47 (s, 1H, H-6); 7.48 (m, 2H, H-*o*-phenylene); 7.52 (m, 2H, H-*o*-Ph); 7.58 (m, 2H, H-*m*-phenylene); 8.02 (s, 1H, H-2); 8.51 (s, 1H, H-5-triazole); ^13^C NMR (150.9 MHz, D_2_O, ref(dioxane) = 69.3 ppm): 40.77 (CH_2_-2′); 68.21 (d, *J*_C,P_ = 4.4, CH_2_-5′); 73.74 (CH-3′); 85.34 (CH-1′); 87.76 (d, *J*_C,P_ = 7.5, CH-4′); 103.15 (C-4a); 119.37 (C-5); 122.25 (CH-5-triazole); 123.32 (CH-6); 123.45 (CH-*m*-phenylene); 127.73 (CH-*o*-Ph); 131.21 (C-*i*-Ph); 131.43 (CH-*p*-Ph); 131.58 (CH-*m*-Ph); 132.00 (CH-*o*-phenylene); 137.05 (C-*i*-phenylene); 137.46 (C-*p*-phenylene); 150.47 (C-4-triazole); 152.63 (C-7a); 153.90 (CH-2); 159.59 (C-4); ^31^P{^1^H} NMR (202.3 MHz, D_2_O): –21.23 (bs, P_β_); –10.32 (bs, P_*α*_); –5.44 (bs, P_γ_); MS (ESI–): *m*/*z* (%): 548.3 (100) [M – H_3_P_2_O_6_], 628.3 (55) [M – H_2_PO_3_], 650.3 (50) [M – H – H_2_PO_3_ + Na], 708.3 (10) [M – H]. HRMS (ESI–): calcd for C_25_H_25_N_7_O_12_P_3_: 708.07795; found 708.07822.

#### 5-[4-(4-(4-Nitrophenyl)-1,2,3-triazol-1-yl)phenyl]-2′-deoxycytidine 5′-*O*-triphosphate (**dC^TNO2^TP**)

Compound **dC^TNO2^TP** was prepared from **dC^TNO2^** according to the general procedure (Method D). The product was isolated as a brown solid (2.5 mg, 18%). ^1^H NMR (600.1 MHz, D_2_O, ref(dioxane) = 3.75 ppm): 2.36 (ddd, 1H, *J*_gem_ = 14.1, *J*_2′b,1′_ = 7.0, *J*_2′b,3′_ = 6.4, H-2′b); 2.48 (ddd, 1H, *J*_gem_ = 14.1, *J*_2′a,1′_ = 6.3, *J*_2′a,3′_ = 3.9, H-2′a); 4.20–4.29 (bm, 3H, H-4′,5′); 4.66 (m, 1H, H-3′); 6.27 (dd, 1H, *J*_1′,2′_ = 7.0, 6.3, H-1′); 7.55 (m, 2H, H-*o*-phenylene); 7.840 (m, 2H, H-*m*-phenylene); 7.843 (s, 1H, H-6); 7.99 (m, 2H, H-*o*-C_6_H_4_NO_2_); 8.28 (m, 2H, H-*m*-C_6_H_4_NO_2_); 8.98 (s, 1H, H-5-triazole); ^13^C NMR (150.9 MHz, D_2_O, ref(dioxane) = 69.3 ppm): 42.19 (CH_2_-2′); 67.81 (d, *J*_C,P_ = 4.6, CH_2_-5′); 72.98 (CH-3′); 88.42 (d, *J*_C,P_ = 8.8, CH-4′); 88.94 (CH-1′); 111.79 (C-5); 124.06 (CH-*m*-phenylene); 124.49 (CH-5-triazole); 127.21 (CH-*m*-C_6_H_4_NO_2_); 129.05 (CH-*o*-C_6_H_4_NO_2_); 133.38 (CH-*o*-phenylene); 136.19 (C-*i*-phenylene); 138.52, 138.55 (C-*i*-C_6_H_4_NO_2_, C-*p*-phenylene); 142.94 (CH-6); 148.76 (C-4-triazole); 149.81 (C-*i*-C_6_H_4_NO_2_); 159.54 (C-2); 166.82 (C-4); ^31^P{^1^H} NMR (202.3 MHz, D_2_O): –21.23 (bm, P_β_); –10.67 (bd, *J* = 16.8, P_α_); –5.47 (bd, *J* = 19.1, P_γ_); MS (ESI–): *m*/*z* (%): 570.3 (80) [M – H_3_P_2_O_6_], 650.2 (95) [M – H_2_PO_3_], 672.2 (100) [M – H – H_2_PO_3_ + Na], 731.2 (10) [M – H]. HRMS (ESI–): calcd for C_23_H_23_N_7_O_15_P_3_: 730.04705; found 730.04741.

#### 7-[4-(4-(4-Nitrophenyl)-1,2,3-triazol-1-yl)phenyl]-7-deaza-2′-deoxyadenosine 5′-*O*-triphosphate (**dA^TNO2^TP**)

Compound **dA^TNO2^TP** was prepared from **dA^TNO2^** according to the general procedure (Method D). The product was isolated as a brown solid (5.5 mg, 18%). ^1^H NMR (600.1 MHz, D_2_O, ref(dioxane) = 3.75 ppm): 2.51 (ddd, 1H, *J*_gem_ = 13.8, *J*_2′b,1′_ = 6.3, *J*_2′b,3′_ = 3.5, H-2'b); 2.68 (bddd, 1H, *J*_gem_ = 13.8, *J*_2′a,1′_ = 7.7, *J*_2′a,3′_ = 6.4, H-2′a); 4.17, 4.22 (2 × bm, 2 × 1H, H-5′); 4.25 (bm, 1H, H-4′); 4.79 (m, 1H, H-3′); 6.34 (bdd, 1H, *J*_1′,2′_ = 7.7, 6.3, H-1′); 7.22 (m, 2H, H-*o*-phenylene); 7.37 (m, 2H, H-*m*-phenylene); 7.40 (s, 1H, H-6); 7.50 (m, 2H, H-*o*-C_6_H_4_NO_2_); 7.79 (m, 2H, H-*m*-C_6_H_4_NO_2_); 7.91 (s, 1H, H-2); 8.51 (s, 1H, H-5-triazole); ^13^C NMR (150.9 MHz, D_2_O, ref(dioxane) = 69.3 ppm: 41.26 (CH_2_-2′); 68.29 (d, *J*_C,P_ = 5.7, CH_2_-5′); 73.85 (CH-3′); 85.34 (CH-1′); 87.82 (d, *J*_C,P_ = 8.8, CH-4′); 102.57 (C-4a); 119.07 (C-5); 122.64 (CH-*m*-phenylene); 123.26 (CH-5-triazole); 123.47 (CH-6); 126.24 (CH-*m*-C_6_H_4_NO_2_); 128.20 (CH-*o*-C_6_H_4_NO_2_); 131.54 (CH-*o*-phenylene); 136.61 (C-*i*-phenylene); 136.66 (C-*p*-phenylene); 137.50 (C-*i*-C_6_H_4_NO_2_); 148.34 (C-4-triazole); 148.85 (C-*p*-C_6_H_4_NO_2_); 152.27 (C-7a); 153.51 (CH-2); 159.14 (C-4); ^31^P{^1^H} NMR (202.4 MHz, D_2_O): –21.39 (bm, P_β_); –10.87 (d, *J* = 18.2, P_α_); –6.25 (bd, *J* = 15.5, P_γ_); MS (ESI–): *m*/*z* (%) 593.3 (50) [M – H_3_P_2_O_6_], 673.3 (100) [M – H_2_PO_3_], 695.3 (90) [M – H – H_2_PO_3_ + Na], 753.3 (5) [M – H]. HRMS (ESI–): calcd for C_25_H_24_N_8_O_14_P_3_: 753.06303; found 753.06312.

#### Primer extension experiment

The reaction mixture (20 μL) contained DNA polymerase [KOD XL, Pwo, Vent (*exo*-)], primer (0.15 μM), template (0.23 μM) and natural and modified dNTPs (0.2 mM) in a reaction buffer. The primer was labeled by use of [γ^32^P]-ATP according to standard techniques. The reaction mixtures were incubated for 15–40 min at 60 °C and analysed by PAGE.

#### Kinetics of PEX (Fig. S3 and S4†)

PEX reaction mixtures that included Pwo DNA polymerase, and temp^C^ and temp^Aterm^ as templates were incubated for specific time intervals (0.1–10 min), and then the reaction was stopped by addition of a PAGE loading buffer and immediate heating.

#### Click reaction of the PEX product DNA^23^ (Fig. 2)

dsDNA obtained by the PEX experiments was purified using Qiagen Nucleotide Removal Kit purification columns. A solution of the Cu(i) catalyst (10 mM) was freshly prepared just before the reaction by mixing CuBr (1 μL, 50 mM in DMSO–*t*BuOH 3 : 1), TBTA ligand (4 μL, 100 mM in DMSO–*t*BuOH 3 : 1) and DMSO–*t*BuOH 3 : 1 (3 μL). To the DNA solution (50 μL, 50 ng μL^–1^), a solution of acetylene (phenylacetylene or 1-ethynyl-4-nitrobenzene) (30 μL, 10 mM in DMSO), sodium ascorbate (2 μL, 5 mM in water), pre-complexed Cu(i) and 10 μL DMSO–*t*BuOH 3 : 1 were added. The mixture was incubated for 2 h at 37 °C and with 500 rpm stirring. After the reaction, the crude mixture was purified once again and then was desalted using dialysis membranes (Millipore).

#### Binding study using p53CD_GST protein: native analysis of reaction mixtures with different p53CD_GST/DNA ratios

The reaction mixture (100 μL) contained primer (prim^15^, 10 μL, 3 μM), template (template^p53_1a2G^/temp^p53_2CON_4^, 12 μL, 3 μM), KOD XL DNA polymerase (1 μL, 2.5 U μl^–1^) and dNTPs (either all natural or 3 natural and 1 modified, 5 μL, 4 mM) in KOD XL reaction buffer (10 μL) supplied by the manufacturer. Primers were labelled on their 5′-end by use of [γ^32^P]-ATP according to standard techniques. The reaction mixture was incubated for 45 min at 60 °C in a thermal cycler and purified using a QIAquick Nucleotide Removal Kit (Qiagen). The PEX-product was eluted from the column using H_2_O (pH 7.7, 50 μL). The reaction mixtures for p53CD_GST protein binding (10 μL) were prepared from the purified PEX-product (5 μL, 10 ng μl^–1^), 50 mM KCl, 5 mM tris pH 7.6 and p53CD_GST stock solution (750 ng μl^–1^, 25 mM Hepes pH 7.6, 200 mM KCl, 10% glycerol, 0.1 mM PPh_3_; 0.4, 0.7, 1.2, 1.7 equiv.). A control sample was prepared analogously without p53CD_GST. All samples were incubated for 45 min on ice, then glycerol was added (60%, 2 μL) and a part of the reaction mixture (3 μL) was separated by use of a 6% native PAGE (acrylamide/bisacrylamide 37.5 : 1; 4 °C, 400 V/2.5 hours). Visualization was performed using phosphoimaging (Fig. 9a, Fig. S8†).

#### Thermal stability of protein–DNA complexes (Fig. S10 and S11†)

The reaction mixtures for p53CD_GST protein binding (40 μL) were prepared from the purified PEX-product (20 μL, 10 ng μl^–1^), 50 mM KCl, 5 mM tris pH 7.6 and p53CD_GST stock solution (750 ng μl^–1^, 25 mM Hepes pH 7.6, 200 mM KCl, 10% glycerol, 0.1 mM PPh_3_; 1.2 equiv.). A control sample was prepared analogously without p53CD_GST. Samples were incubated for 45 min on ice and then were divided into four vials and exposed to four different temperatures (0 °C, 20 °C, 37 °C, 50 °C) for 1 hour, then glycerol was added (60%, 2 μL) and a part of the reaction mixture (3 μL) was separated by use of a 6% native PAGE (acrylamide/bisacrylamide 37.5 : 1; 4 °C, 400 V/2.5 hours). Visualization was performed using phosphoimaging.

#### Cu^I^ concentration dependence of the stability of the protein–DNA complexes (Fig. S12 and S13†)

The reaction mixtures for p53CD_GST protein binding (80 μL) were prepared from the purified PEX-product (40 μL, 10 ng μl^–1^), 50 mM KCl, 5 mM tris pH 7.6 and p53CD_GST stock solution (750 ng μl^–1^, 25 mM Hepes pH 7.6, 200 mM KCl, 10% glycerol, 0.1 mM PPh_3_; 1.2 equiv.). A control sample was prepared analogously without p53CD_GST. Samples were incubated for 45 min on ice and then were divided into eight vials and were incubated with various concentration of CuBr (5 μM, 10 μM, 20 μM) with/without the presence of the ligand TBTA at 20 °C for 1 h, then glycerol was added (60%, 2 μL) and a part of the reaction mixture (3 μL) was separated by use of a 6% native PAGE (acrylamide/bisacrylamide 37.5 : 1; 4 °C, 400 V/2.5 hours). Visualization was performed using phosphoimaging.

#### Stability of the protein–DNA complex after click reaction of the DNA (Fig. 9b and S9†)

The reaction mixtures for p53CD_GST protein binding (10 μL) were prepared from the purified PEX-product (5 μL, 10 ng μl^–1^), 50 mM KCl, 5 mM tris pH 7.6 and p53CD_GST stock solution (750 ng μl^–1^, 25 mM Hepes pH 7.6, 200 mM KCl, 10% glycerol, 0.1 mM PPh_3_; 1.2 equiv.). A control sample was prepared analogously without p53CD_GST. The samples were incubated for 45 min on ice and then 4-nitrophenylacetylene (10 mM in MeOH, 0.75 μL), CuBr (100 μM in DMSO–*t*BuOH 3 : 1, 0.75 μL), TBTA (1 mM in DMSO–*t*BuOH 3 : 1, 0.4 μL), sodium ascorbate (5 mM in water, 0.2 μL), KCl (50 mM, 1.5 μL) and tris (5 mM, pH 7.6, 1.5 μL) were added and the reaction mixture was incubated at 20 °C for 1 h. Then glycerol was added (60%, 2 μL) and a part of the reaction mixture (3 μL) was separated by use of a 6% native PAGE (acrylamide/bisacrylamide 37.5 : 1; 4 °C, 400 V/2.5 hours). Visualization was performed using phosphoimaging. For the electrochemical measurements we applied the same conditions described above but in higher scale (increased 5 times), using three parallel samples for exact comparison (DNA mixed with binding protein, control sample – DNA mixed with BSA). BSA was used as control protein.

#### Electrochemical analysis

Nucleosides, dNTPs and other building blocks were analyzed using conventional *in situ* cyclic voltammetry (CV). The PEX products were analyzed using *ex situ* (adsorptive transfer stripping, AdTS) CV or square-wave voltammetry (SWV). The PEX products (purified in their single-stranded form using streptavidin-coated magnetic beads or in their double-stranded forms using a Qiagen Nucleotide Removal Kit) were accumulated at the surface of a working electrode (hanging mercury drop electrode, HMDE) for 60 s, from 5 μL aliquots containing 0.2 M NaCl. The electrode was then rinsed with deionized water and placed into a electrochemical cell. CV settings: scan rate 1 V s^–1^, initial potential 0.0 V, for switching potentials see figure legends. SWV settings: initial potential 0 V, for final potentials see figure legends; frequency 200 Hz, amplitude 50 mV. Background electrolyte: 0.5 M ammonium formate, 0.05 M sodium phosphate, pH 6.9. All measurements were performed at room temperature using an Autolab analyzer (Eco Chemie, The Netherlands) in connection with VA-stand 663 (Metrohm, Herisau, Switzerland). The three-electrode system was used with a Ag/AgCl/3 M KCl electrode as a reference and platinum wire as an auxiliary electrode. Measurements of reduction signals were performed after deaeration of the solution by argon purging.

## References

[cit1] (a) Paleček E., Jelen F. in Electrochemistry of nucleic acids and proteins: Towards electrochemical sensors for genomics and proteomics, ed. E. Paleček, F. Scheller, J. Wang, Elsevier, Amsterdam, 2005, pp. 74–174.

[cit2] Hocek M., Fojta M. (2011). Chem. Soc. Rev..

[cit3] Vrábel M., Horáková P., Pivoňková H., Kalachova L., Černocká H., Cahová H., Pohl R., Šebest P., Havran L., Hocek M., Fojta M. (2009). Chem.–Eur. J..

[cit4] Gorodetsky A. A., Buzzeo M. C., Barton J. K. (2008). Bioconjugate Chem..

[cit5] Horakova P., Macickova-Cahova H., Pivonkova H., Spacek J., Havran L., Hocek M., Fojta M. (2011). Org. Biomol. Chem..

[cit6] Review: Dey B. Thukral S. Krishnan S. Chakrobarty M. Gupta S. Manghani C. Rani V., Mol. Cell. Biochem., 2012, 365 , 279 –299 . 22399265 10.1007/s11010-012-1269-z

[cit7] Galas D. J., Schmitz A. (1978). Nucleic Acids Res..

[cit8] (a) Review: Kolb H. C. Finn M. G. Sharpless K. B., Angew. Chem., Int. Ed., 2001, 40 , 2004 –2021 . 10.1002/1521-3773(20010601)40:11<2004::AID-ANIE2004>3.0.CO;2-5 11433435

[cit9] (a) Review: El-Sagheer A. H. Brown T., Chem. Soc. Rev., 2010, 39 , 1388 –1405 . 20309492 10.1039/b901971p

[cit10] Gramlich P. M. E., Wirges C. T., Manetto A., Carell T. (2008). Angew. Chem., Int. Ed..

[cit11] Neef A. B., Luedtke N. W. (2014). ChemBioChem.

[cit12] Review on chemistry of azides: Bräse S. Gil C. Knepper K. Zimmermann V., Angew. Chem., Int. Ed., 2005, 44 , 5188 –5240 . 10.1002/anie.200400657 16100733

[cit13] Jäger S., Rasched G., Kornreich-Leshem H., Engeser M., Thum O., Famulok M. (2005). J. Am. Chem. Soc..

[cit14] Cho Y. A., Kim D., Ahn H. R., Canturk B., Molander G. A., Ham J. (2009). Org. Lett..

[cit15] For related CuAAC reactions of 2-azidopurines, see: Cosyn L. Palaniappan K. K. Kim S.-K. Duong H. T. Gao Z.-G. Jacobson K. A. van Calenbergh S., J. Med. Chem., 2006, 49 , 7373 –7383 . 17149867 10.1021/jm0608208 PMC4968940

[cit16] Kovacs T., Ötvös L. (1988). Tetrahedron Lett..

[cit17] Matlashewski G., Lamb P., Pim D., Peacock J., Crawford L., Benchimol S. (1984). EMBO J..

[cit18] Brazdova M., Palecek J., Cherny D. I., Billova S., Fojta M., Pecinka P., Vojtesek B., Jovin T. M., Palecek E. (2002). Nucleic Acids Res..

[cit19] El-Deiry W. S., Kern S. E., Pietenpol J. A., Kinzler K. W., Vogelstein B. (1992). Nat. Genet..

[cit20] Dadová J., Orság P., Pohl R., Brázdová M., Fojta M., Hocek M. (2013). Angew. Chem., Int. Ed..

[cit21] Paleček E., Brázdová M., Černocká H., Vlk D., Brázda V., Vojtešek B. (1999). Oncogene.

[cit22] Sumranjit J., Chung S. J. (2013). Molecules.

[cit23] Ménová P., Raindlová V., Hocek M. (2013). Bioconjugate Chem..

