# Supplementary material for: Azidophenyl as a click-transformable redox label of DNA suitable for electrochemical detection of DNA–protein interactions
Source: Chem Sci. 2014 Sep 16;6(1):575–87. doi: 10.1039/c4sc01906g (PMC5618110; doi:10.1039/c4sc01906g)
Supplement: Supplementary file 1 [file SC-006-C4SC01906G-s001.pdf]

Electronic Supplementary Material (ESI) for Chemical Science.
This journal is © The Royal Society of Chemistry 2014

Electronic Supporting Information

for

Azidophenyl as a click-transformable redox label of DNA suitable for electrochemical footprinting of DNA-protein interactions

Jana Balintová,[a] Jan Špaček,[b] Radek Pohl,[a] Marie Brázdová,[b] Luděk Havran,[b,c] Miroslav Fojta*[b,c] and Michal Hocek*[a,d]

[a] Institute of Organic Chemistry and Biochemistry, Academy of Sciences of the Czech Republic, Gilead & IOCB Research Center, Flemingovo nam. 2, CZ-16610 Prague 6, Czech Republic; E-mail: hocek@uochb.cas.cz.
[b] Institute of Biophsics, v.v.i. Academy of Sciences of the Czech Republic; Kralovopolska 135, 61265 Brno, Czech Republic; E-mail: fojta@ibp.cz.
[c] Central European Institute of Technology, Masaryk University Kamenice 753/5, CZ-625 00 Brno, Czech Republic
[d] Department of Organic and Nuclear Chemistry, Faculty of Science, Charles University in Prague, Hlavova 8, CZ-12843 Prague 2, Czech Republic.

Table of contents:

1. Single incorporation of functionalized dNTPs 2
2. Kinetics study 3
3. Multiple incorporations of functionalized dNTPs 4
4. Study of DNA-protein interaction 6
5. Thermal stability of complex DNA-protein 8
6. Cu^{I} concentration dependence of stability of complex DNA-protein 10
7. Electrochemistry 12
8. Copies of NMR spectra 16
9. Copies of Maldi-TOF spectra of DNA 31
10. Copies of Maldi-TOF spectra 33

1 Single incorporation of functionalized dNTPs

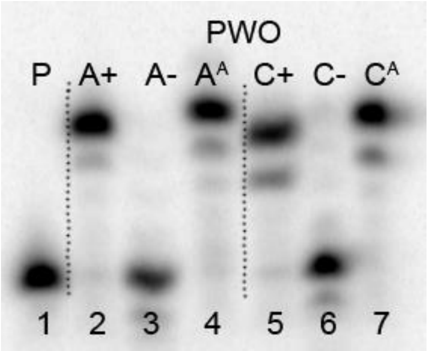

Figure S1. PEX single incorporations of a **dN^A^TP** into 19-nt DNA using temp^{C} or temp^{A} template and PWO DNA polymerase.

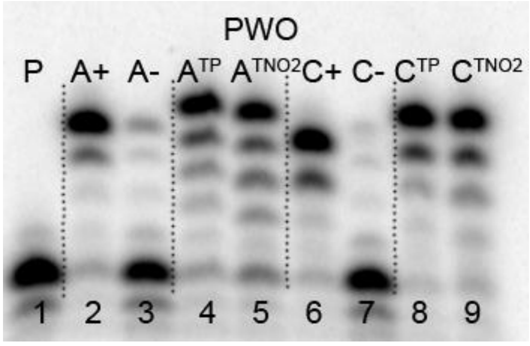

Figure S2. PEX single incorporations of a **dN^{TP}TP** and **dN^{TNO2}TP** into 19-nt DNA using temp^{C} or temp^{A} template and Pwo DNA polymerase.

2 Kinetics study

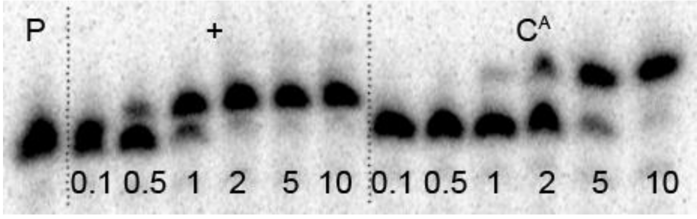

Figure S3. Kinetics of PEX using **dC^{A}TP** in comparison with natural **dCTP** (+). Time intervals are given in minutes.

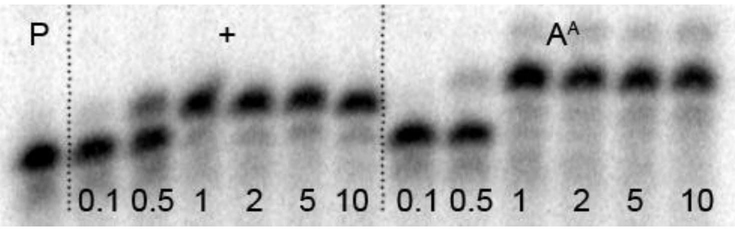

Figure S4. Kinetics of PEX using **dA^{A}TP** in comparison with natural **dATP** (+). Time intervals are given in minutes.

3 Multiple incorporations of functionalized dNTPs

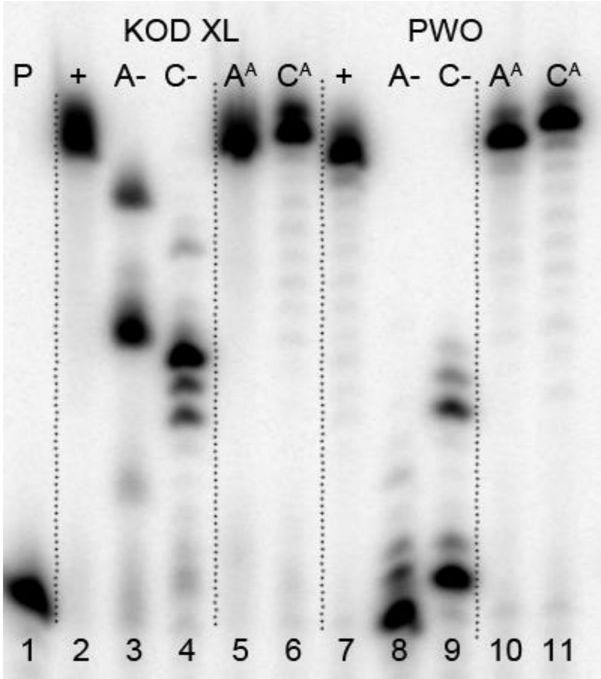

Figure S5. PEX incorporations of a **dN$^{\mathbf{A}}$TP** into 31-nt DNA using temp^{rnd16} template, KOD XL and PWO DNA polymerase.

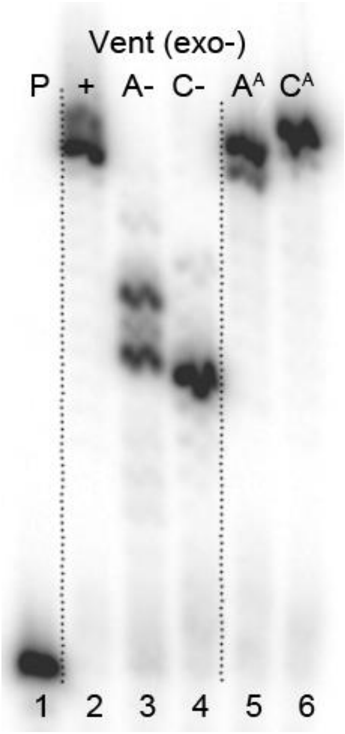

Figure S6. PEX incorporations of a **dN$^{\mathbf{A}}$TP** into 31-nt DNA using temp^{rnd16} template, Vent (*exo-)* DNA polymerase.

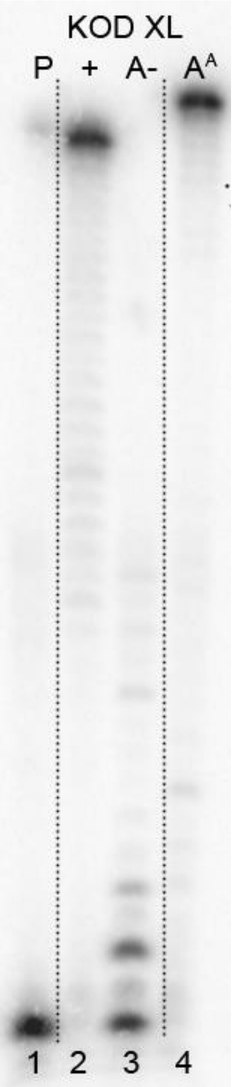

Figure S7. PEX incorporations of a **dN$^{\mathbf{A}}$TP** into 50-nt DNA using template temp^{2CON4} and KOD XL DNA polymerase.

4 Study of DNA-protein interaction

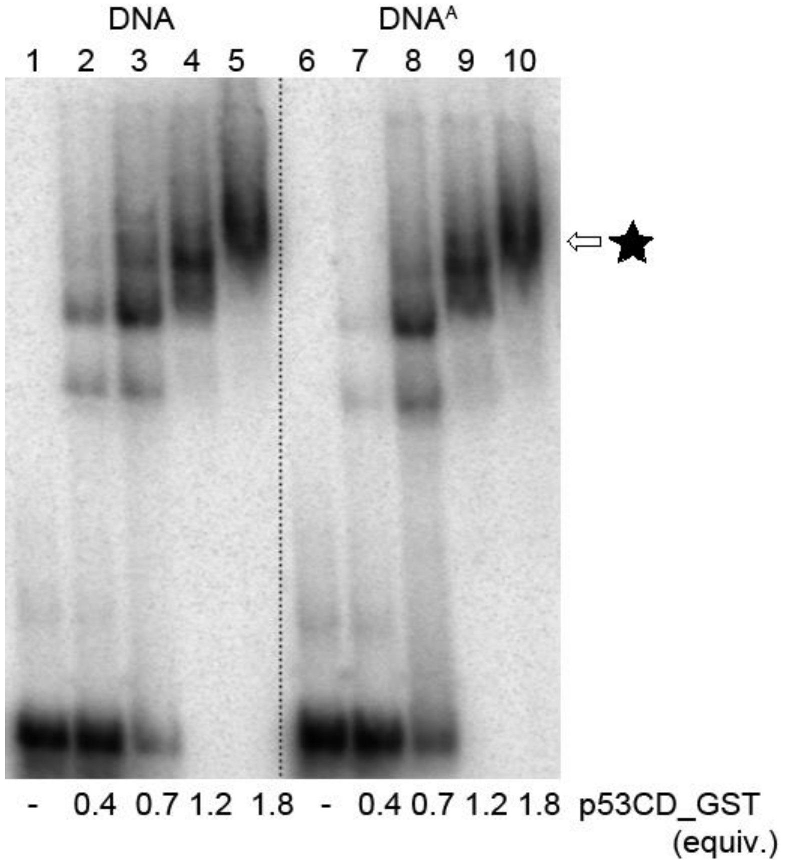

Figure S8. Native PAGE analysis of 50-mer DNA^{2CON4}_p53CD_GST complex. Lane 1: natural DNA; 2: 0.4 equiv.; 3: 0.7 equiv.; 4: 1.2 equiv.; 5: 1.8 equiv. of protein p53CD_GST to DNA; Lane 6: DNA^{A}; 7: 0.4 equiv.; 8: 0.7 equiv.; 9: 1.2 equiv.; 10: 1.8 equiv. of protein p53CD_GST to DNA.

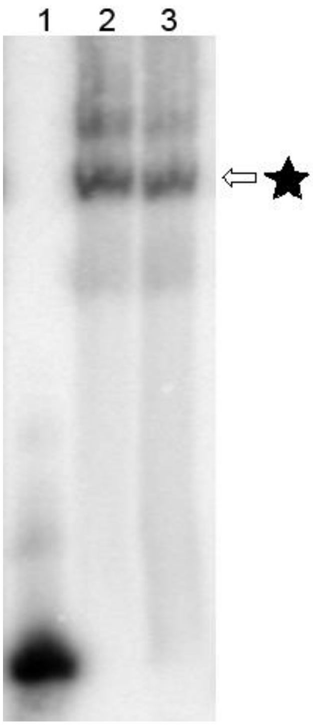

Figure S9. Native PAGE analysis of stability of DNA^{2CON4}_p53CD_GST complex after click reaction. Lane 1: DNA^{A}; lane 2: protein/DNAcomplex; lane 3: protein/DNAcomplex, 0.5 mM 4-nitrophenylacetylene, 5 μM CuBr; 25 μM TBTA ligand, 65 μM Na ascorbate, 20 ºC, 1h.

5 Thermal stability of complex DNA-protein

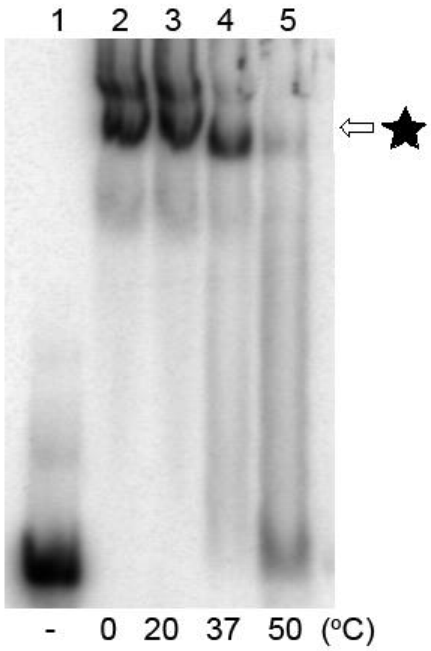

Figure S10. Native PAGE analysis of thermal stability of DNA^{1a2G}_p53CD_GST complex. Lane 1: DNA^{A}; lanes 2-5: 1.2 equiv. of protein p53CD_GST to DNA. **Conditions:** DNA and proteins were mixed together in binding buffer and created protein/DNA complexes were incubated at mentioned temperatures for 1h.

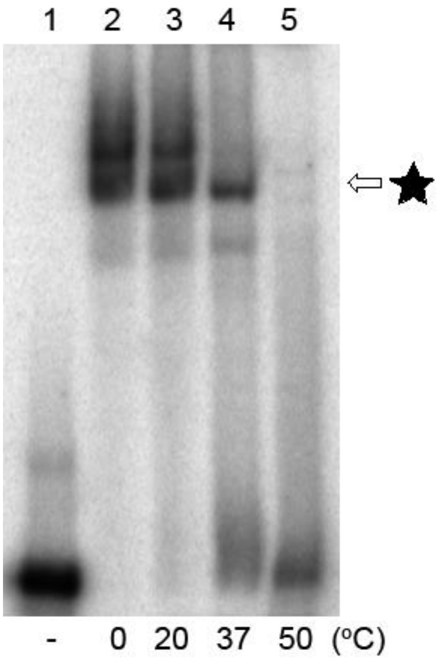

Figure S11. Native PAGE analysis of thermal stability of DNA^{2CON4}_p53CD_GST complex. Lane 1: DNA^{A}; lanes 2-5: 1.2 equiv. of protein p53CD_GST to DNA. **Conditions:** DNA and proteins were mixed together in binding buffer and created protein/DNA complexes were incubated at mentioned temperatures for 1h.

6 Cu^I concentration dependence of stability of complex DNA-protein

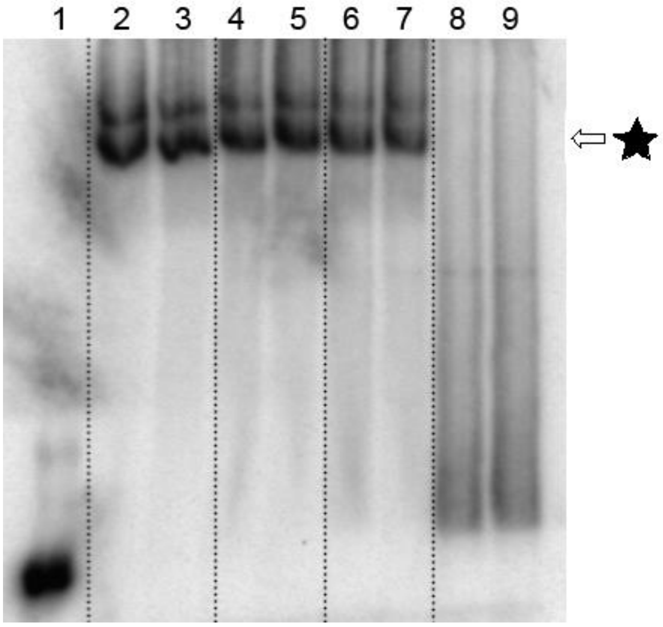

Figure S12. Native PAGE analysis of Cu^I concentration dependence of stability of DNA^{1a2G}_p53CD_GST complex. Lane 1: DNA^A; lanes 2-9: 1.2 equiv. of protein p53CD_GST to DNA; lane 2: protein/DNAcomplex; lane 3: protein/DNAcomplex, 20ºC; lane 4: protein/DNAcomplex, 5 μM CuBr, 20ºC; lane 5: protein/DNAcomplex, 5 μM CuBr, 25 μM TBTA ligand, 20ºC; lane 6: protein/DNAcomplex, 10 μM CuBr, 20ºC; lane 7: protein/DNAcomplex, 10 μM CuBr; 50 μM TBTA ligand, 20ºC; lane 8: protein/DNAcomplex, 20 μM CuBr, 20ºC; lane 9: protein/DNAcomplex, 20 μM CuBr; 100 μM TBTA ligand, 20ºC. **Conditions:** DNA and proteins were mixed together in binding buffer and created protein/DNAcomplexes were incubated with various concentration of CuBr in/without presence of the TBTA ligand at 20ºC for 1h.

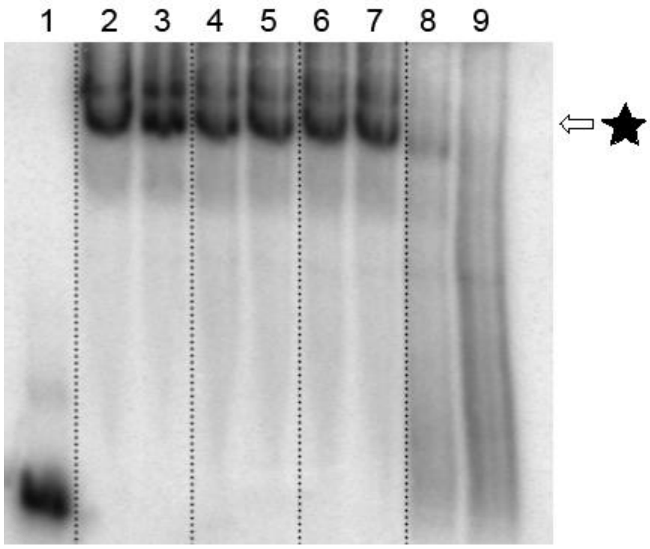

Figure S13. Native PAGE analysis of Cu^{I} concentration dependence of stability of DNA^{2CON4}_p53CD_GST complex. Lane 1: DNA^{A}; lanes 2-9: 1.2 equiv. of protein p53CD_GST to DNA; lane 2: protein/DNAcomplex; lane 3: protein/DNAcomplex, 20ºC; lane 4: protein/DNAcomplex, 5µ M CuBr, 20ºC; lane 5: protein/DNAcomplex, 5 µM CuBr, 25 µM TBTA ligand, 20ºC; lane 6: protein/DNAcomplex, 10 µM CuBr, 20ºC; lane 7: protein/DNAcomplex, 10 µM CuBr; 50 µM TBTA ligand, 20ºC; lane 8: protein/DNAcomplex, 20 µM CuBr, 20ºC; lane 9: protein/DNAcomplex, 20 µM CuBr; 100 µM TBTA ligand, 20ºC. **Conditions:** DNA and proteins were mixed together in binding buffer and created protein/DNAcomplexes were incubated with various concentration of CuBr in/without presence of the TBTA ligand at 20ºC for 1h.

7 Electrochemistry

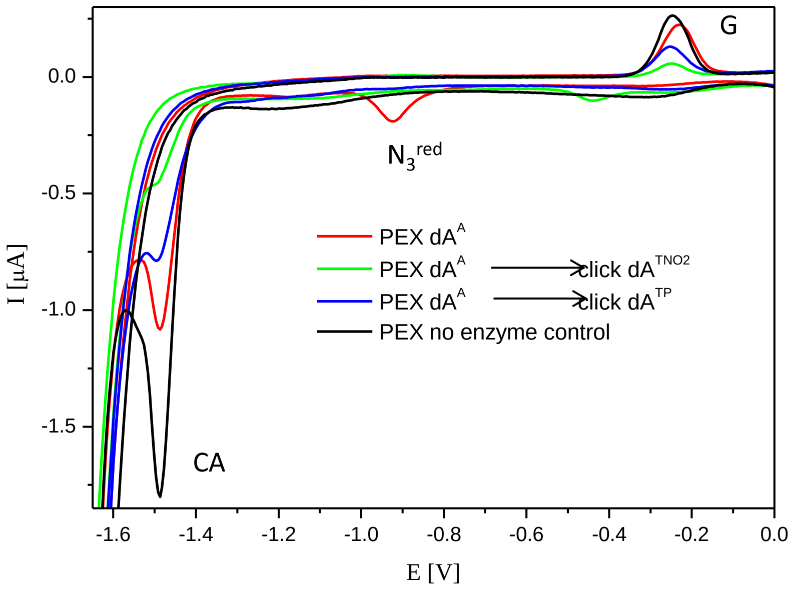

Figure S14. CV responses at HMDE of PEX products synthesized with temp*rnd16* template and dNTP mixes containing **dA$^{\mathbf{A}}$TP** (as specified in legend) complemented with three natural dNTPs and PEX products after click reaction with (nitro)phenyltriazole. CA – peak due to reduction of cytosine and adenine, G – peak due to guanine, N_3^{red} – azide reduction, NO_2^{red} – nitrogroup reduction.

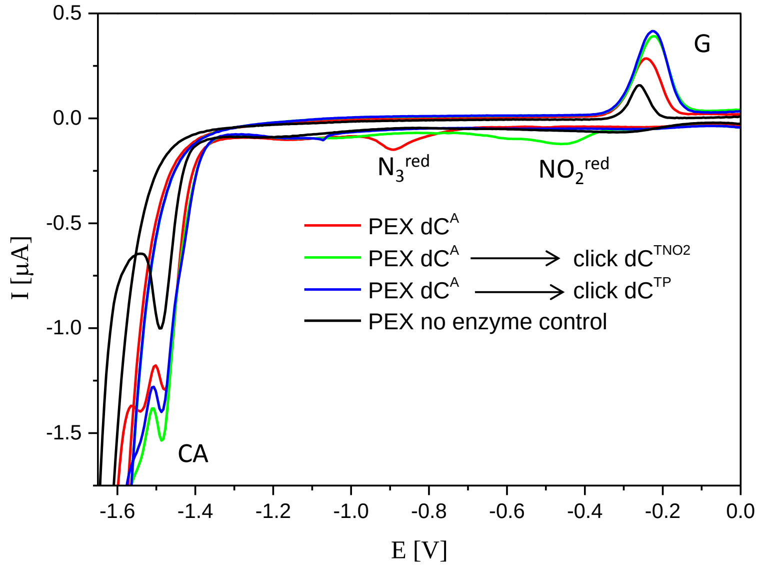

Figure S15. CV responses at HMDE of PEX products synthesized with temp*rnd16* template and dNTP mixes containing **dC$^{\mathbf{A}}$TP** (as specified in legend) complemented with three natural dNTPs and PEX products after click reaction with (nitro)phenyltriazole.

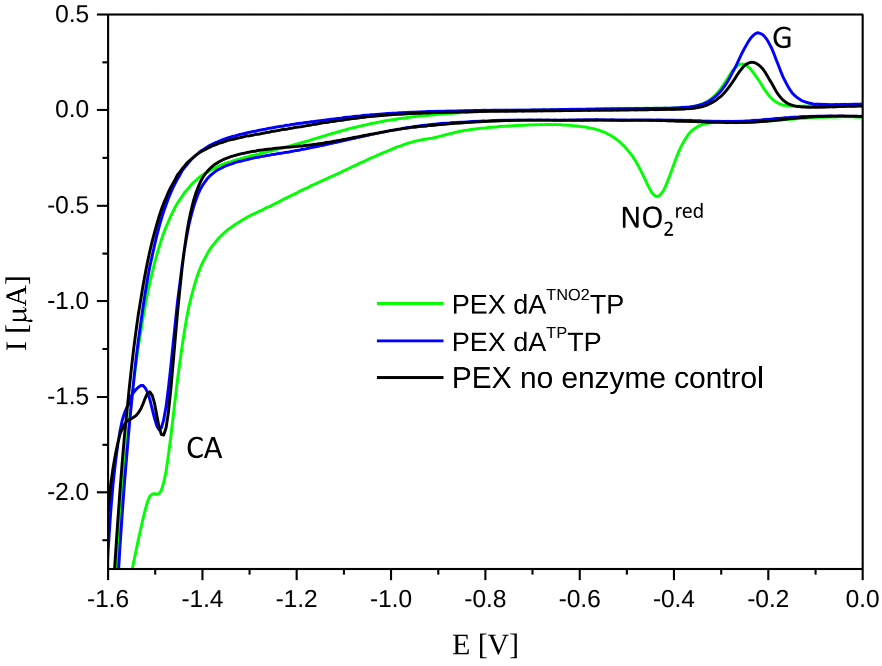

Figure S16. CV responses at HMDE of PEX products synthesized with temp*rnd16* template and dNTP mixes containing **dA^{TX}TP** (as specified in legend) complemented with three natural dNTPs.

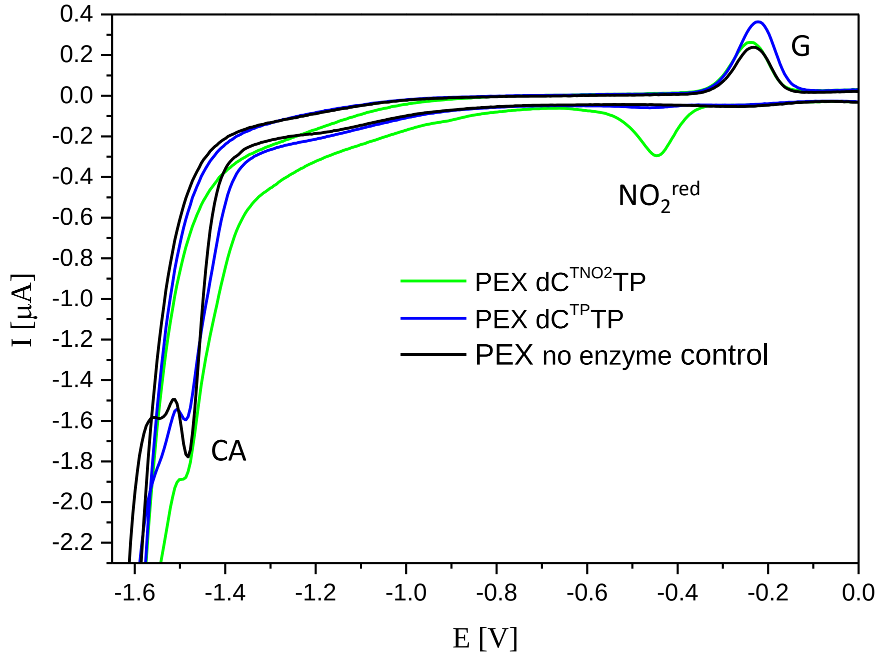

Figure S17. CV responses at HMDE of PEX products synthesized with temp*rnd16* template and dNTP mixes containing **dC^{TX}TP** (as specified in legend) complemented with three natural dNTPs.

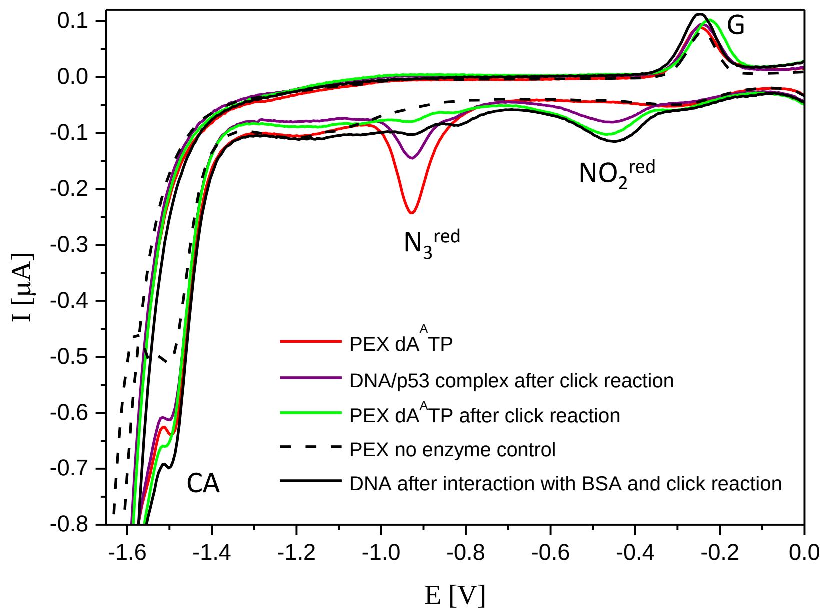

Figure S18. CV responses at HMDE of PEX products synthesized with temp^{1a2G} template and **dA$^{\mathbf{A}}$TP** complemented with three natural dNTPs (red curve) and PEX products after click reaction with nitrophenylacetylene (green curve), DNA-p53 complex after click reaction followed by denaturation (violet curve), control with BSA (black curve).

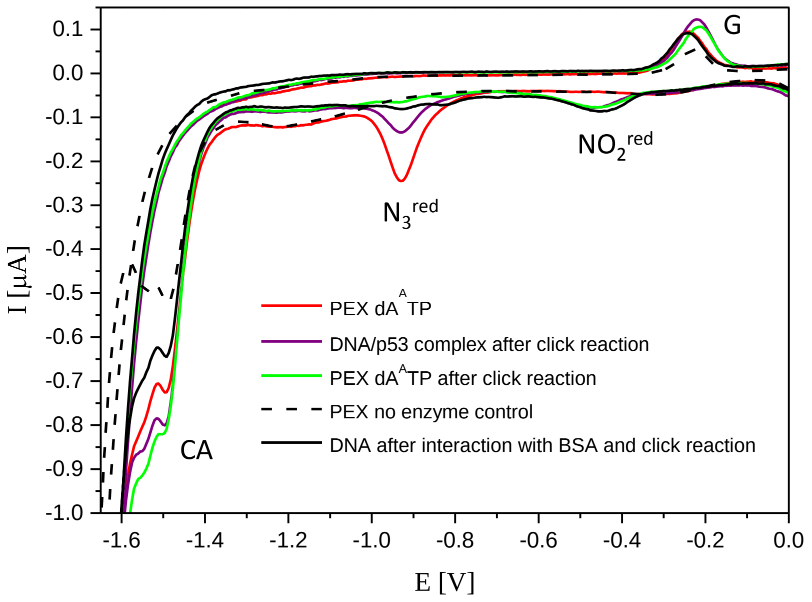

Figure S19. CV responses at HMDE of PEX products synthesized with temp^{2CON4} template and **dA$^{\mathbf{A}}$TP** complemented with three natural dNTPs (red curve) and PEX products after click reaction with nitrophenylacetylene (green curve), DNA-protein complex after click reaction followed by denaturation (violet curve), control with BSA (black curve).

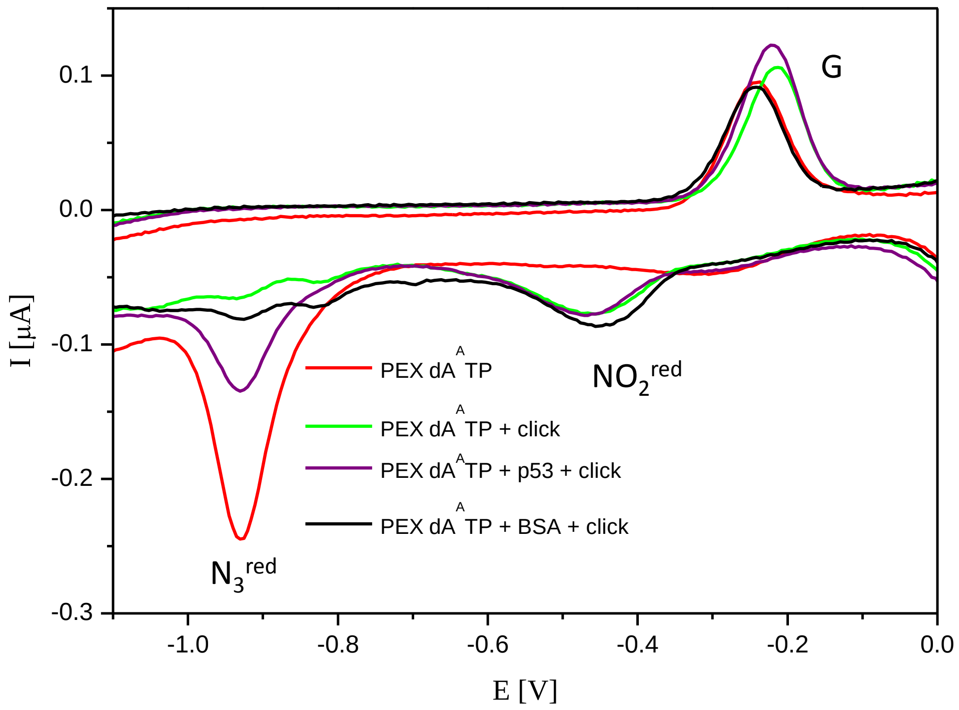

Figure S20. Detail of CV responses at HMDE of PEX products synthesized with temp*2CON4* template and **dA^A^TP** complemented with three natural dNTPs (red curve) and PEX products after click reaction with nitrophenylacetylene (green curve), DNA-protein complex after click reaction followed by denaturation (violet curve), the control with BSA (black curve). For full CV scans see Fig. S19.

8 Selected copies of NMR spectra

^{1}H NMR and ^{13}C spectra of **dC^{A}.**

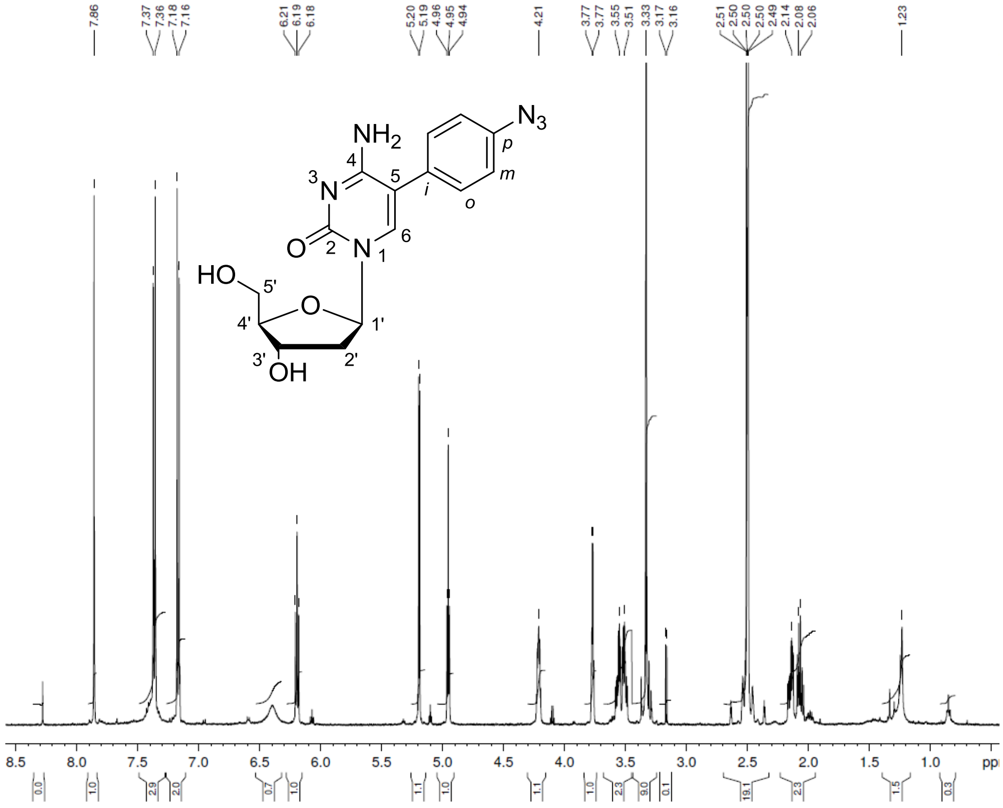

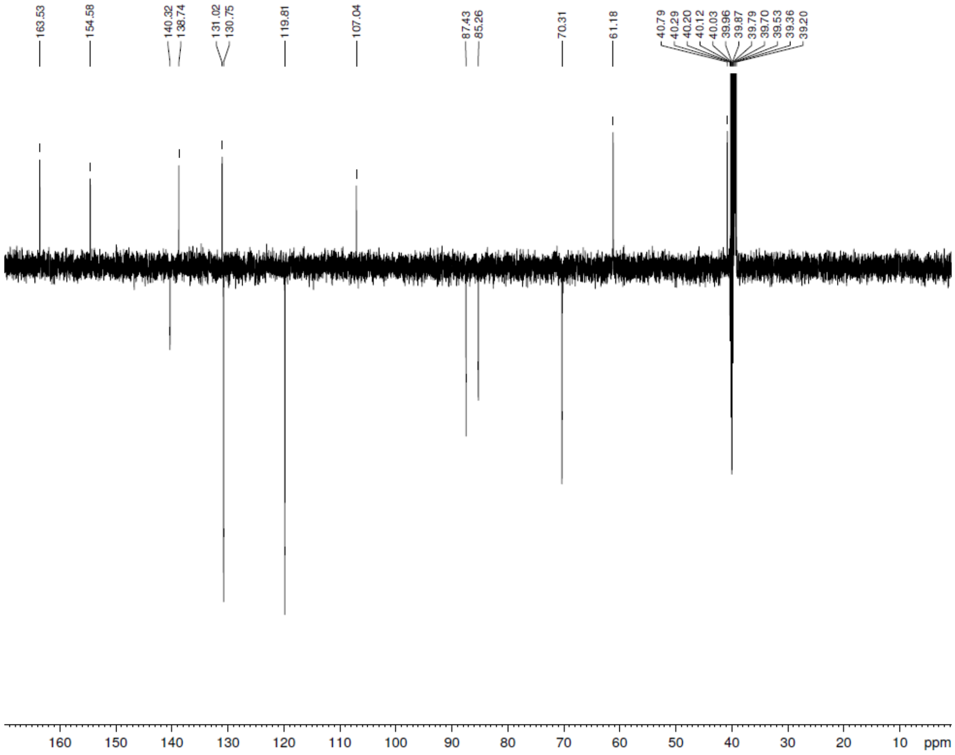

^{1}H NMR and ^{13}C spectra of **dA^A^**.

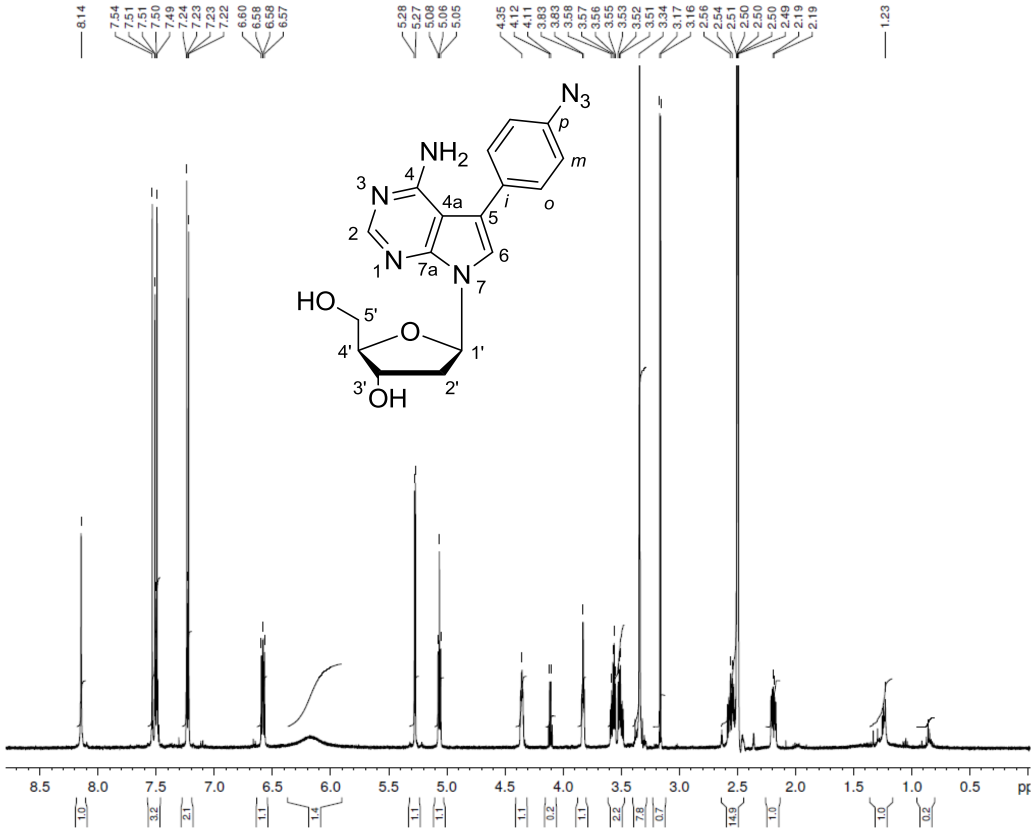

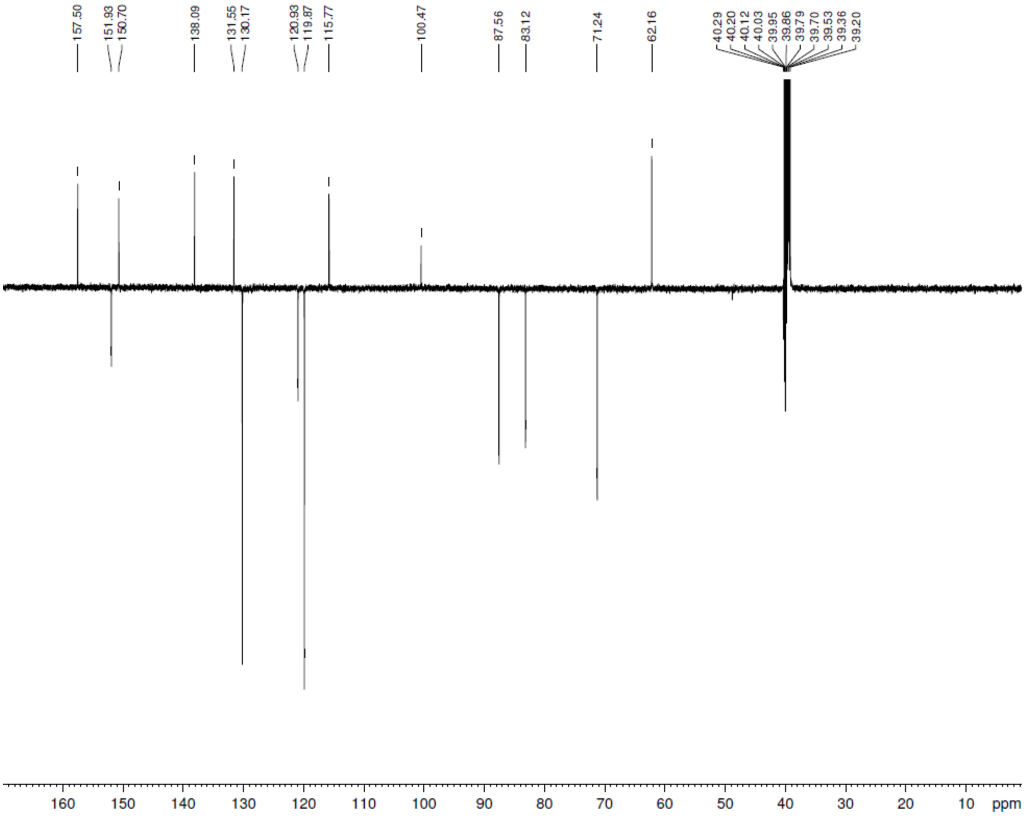

^{1}H NMR and ^{13}C spectra of **dC^{TP}.**

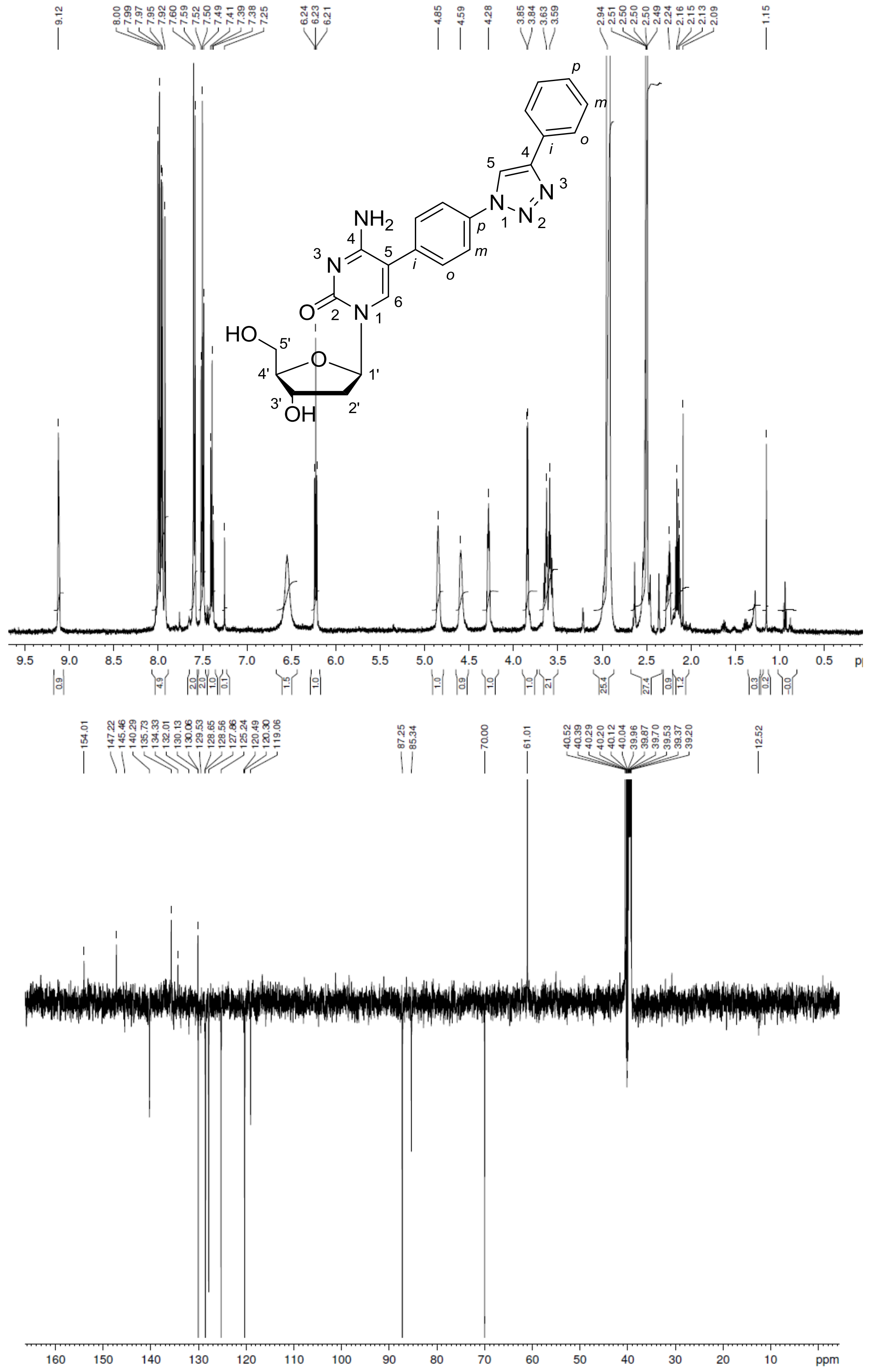

^{1}H NMR and ^{13}C spectra of **dA^{TP}.**

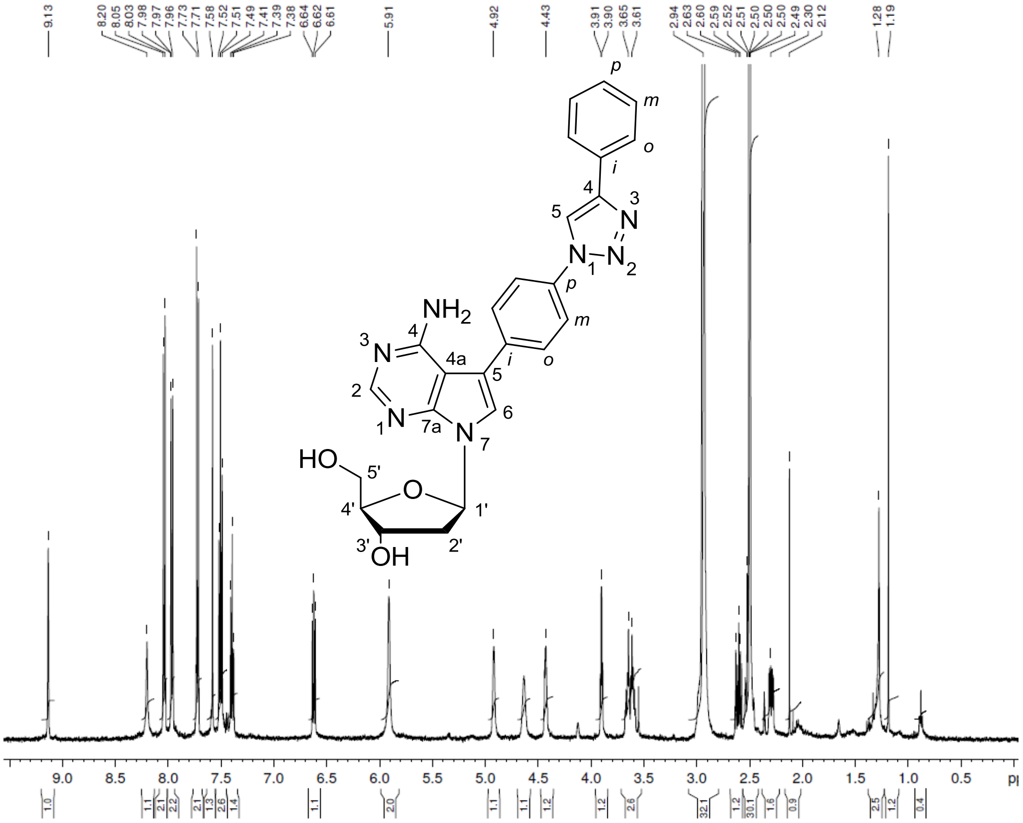

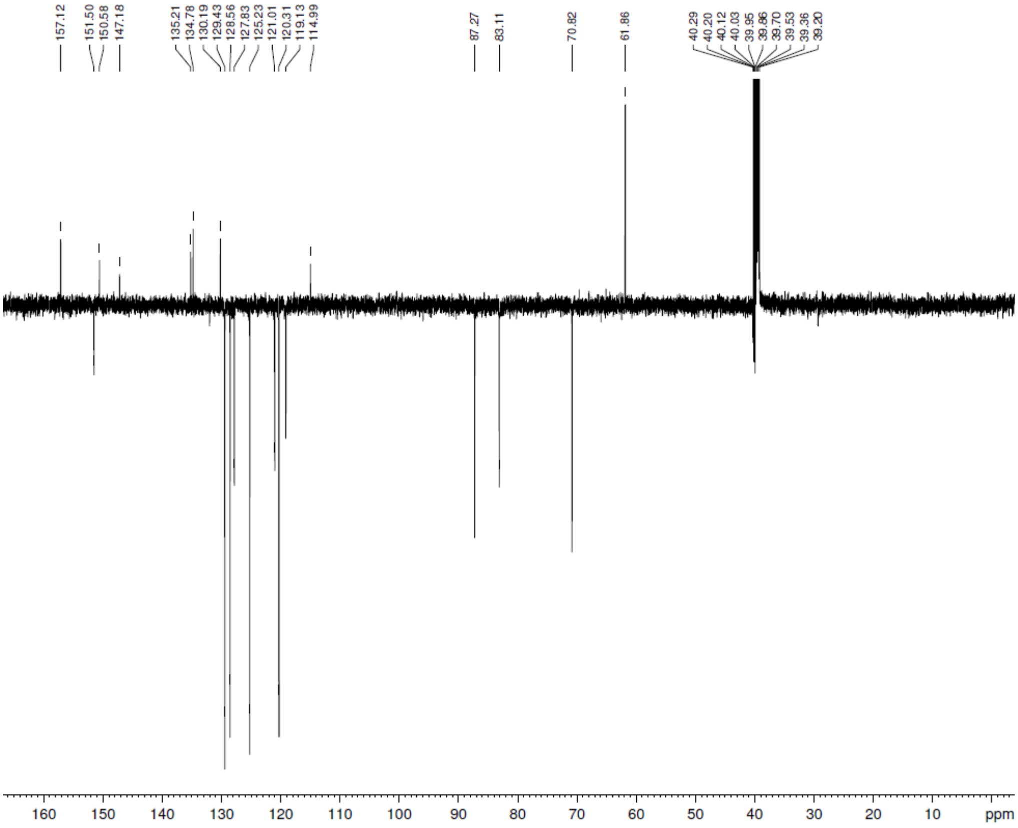

^{1}H NMR and ^{13}C spectra of **dC$^{\mathbf{TNO2}}$.**

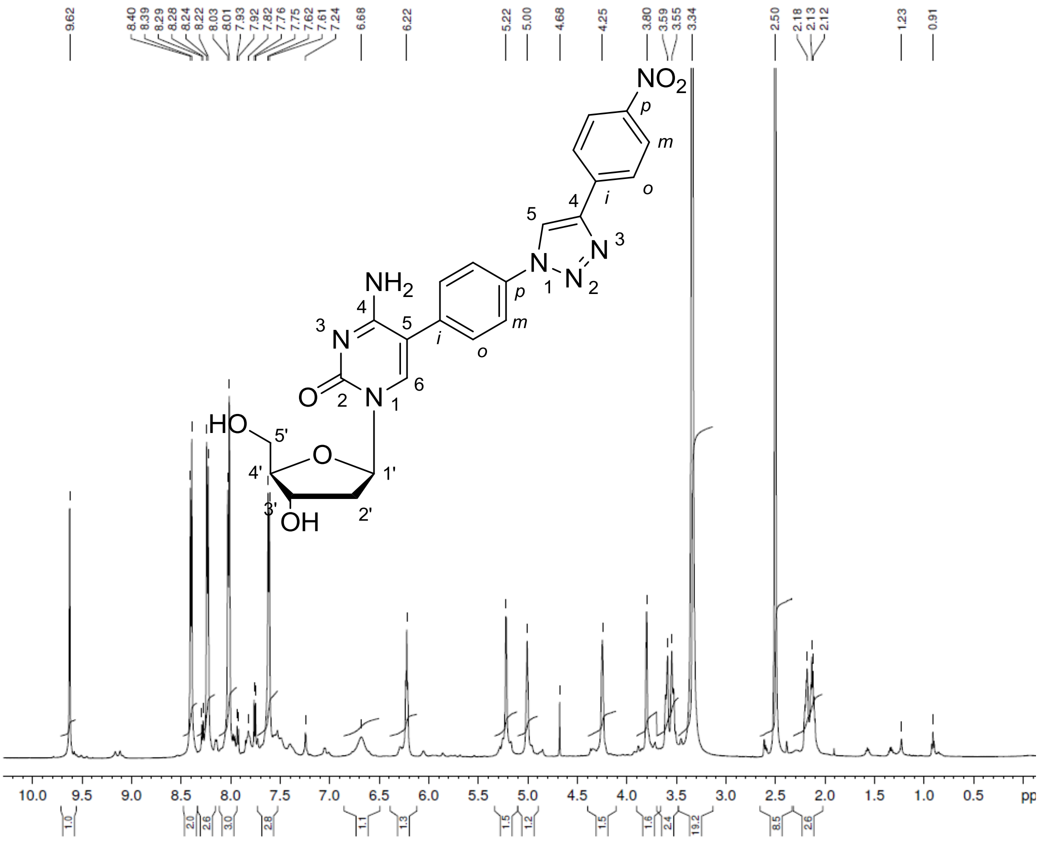

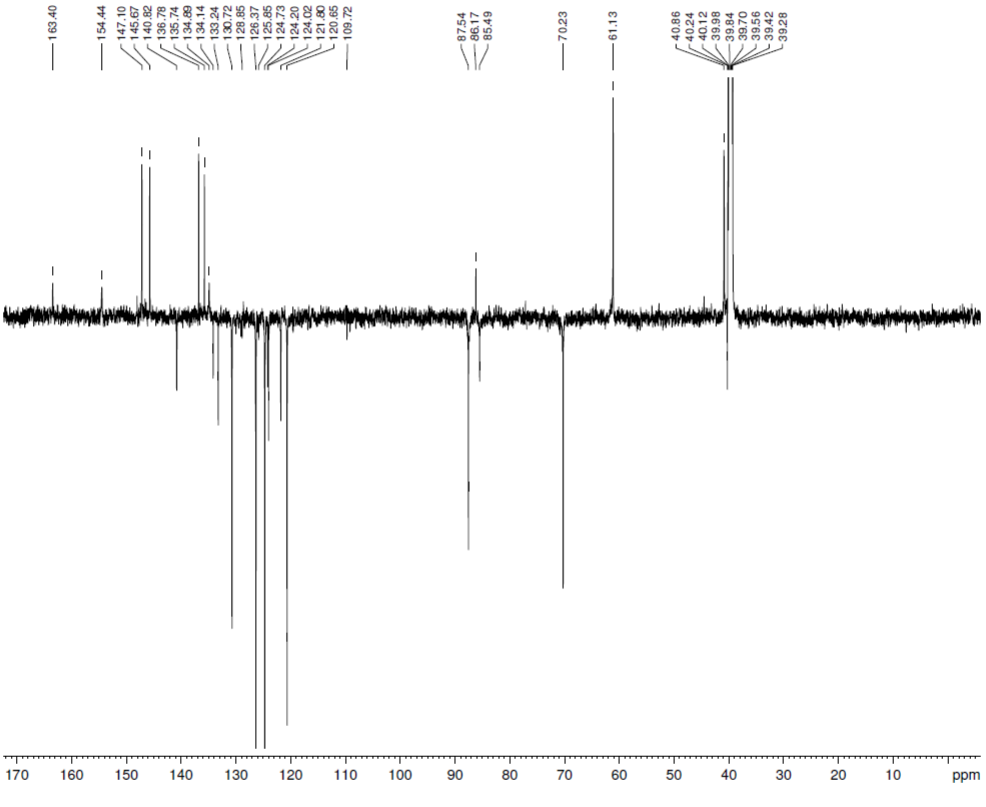

^{1}H NMR and ^{13}C spectra of **dA^{TNO2}.**

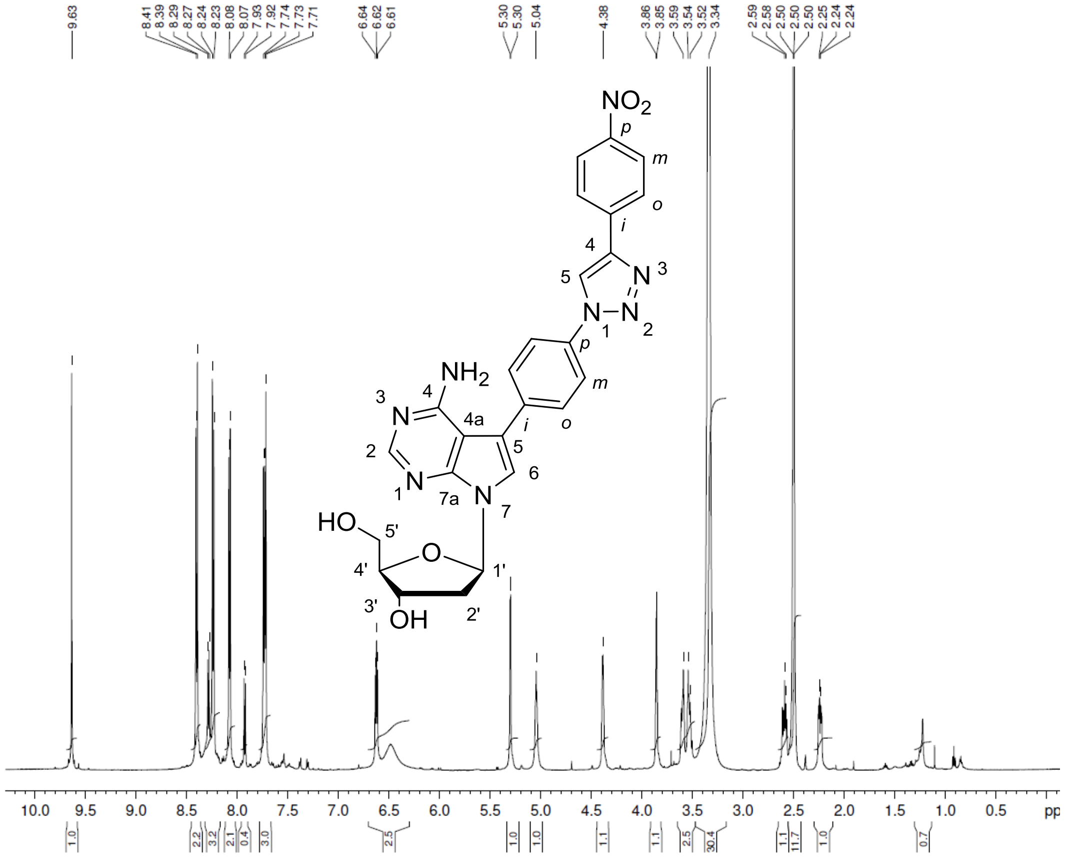

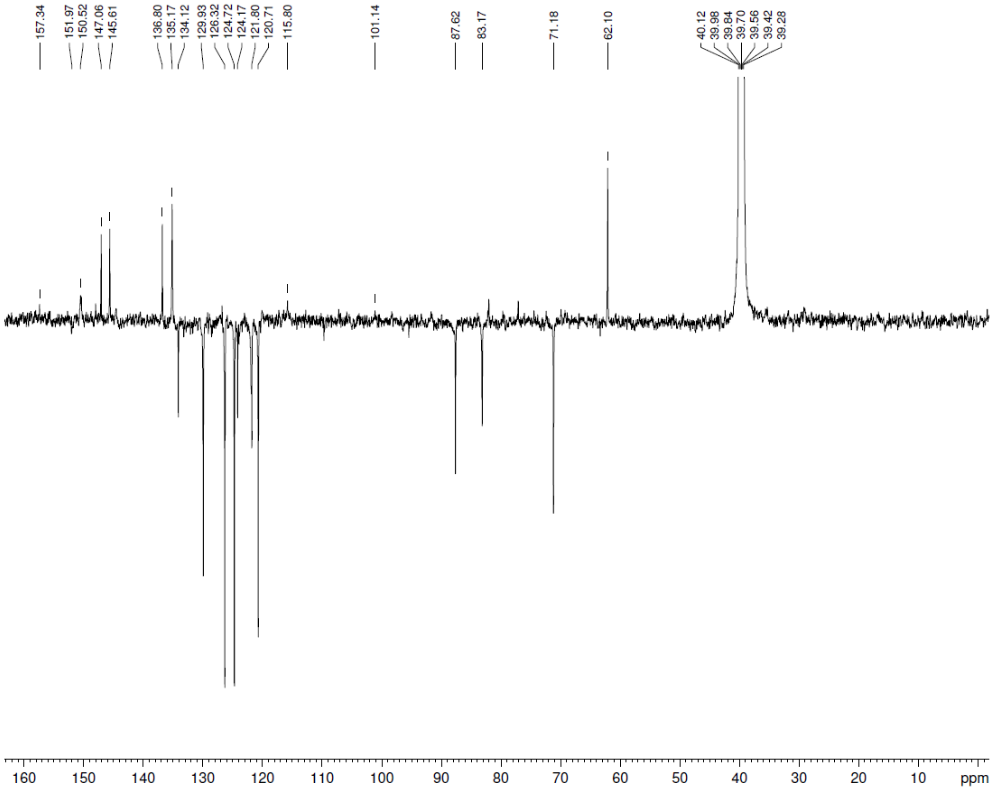

^{1}H NMR, ^{13}C and ^{31}P spectra of **dC$^{\mathbf{A}}$TP.**

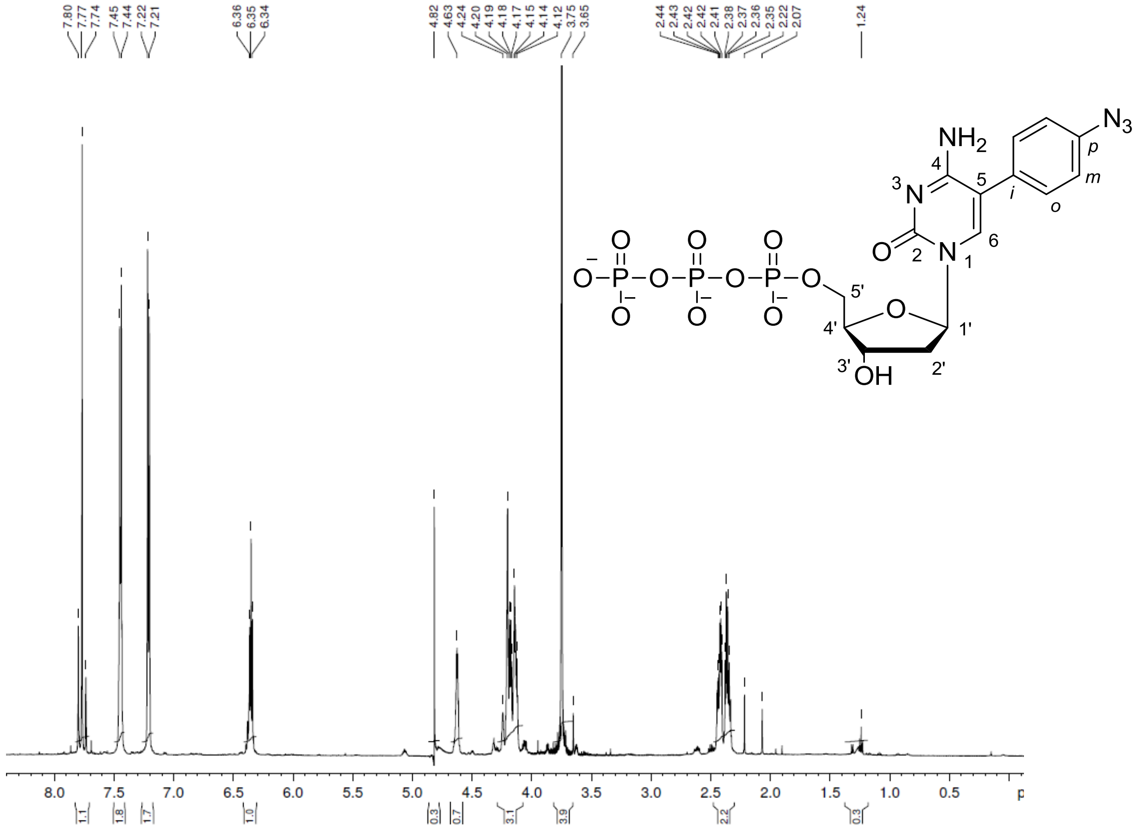

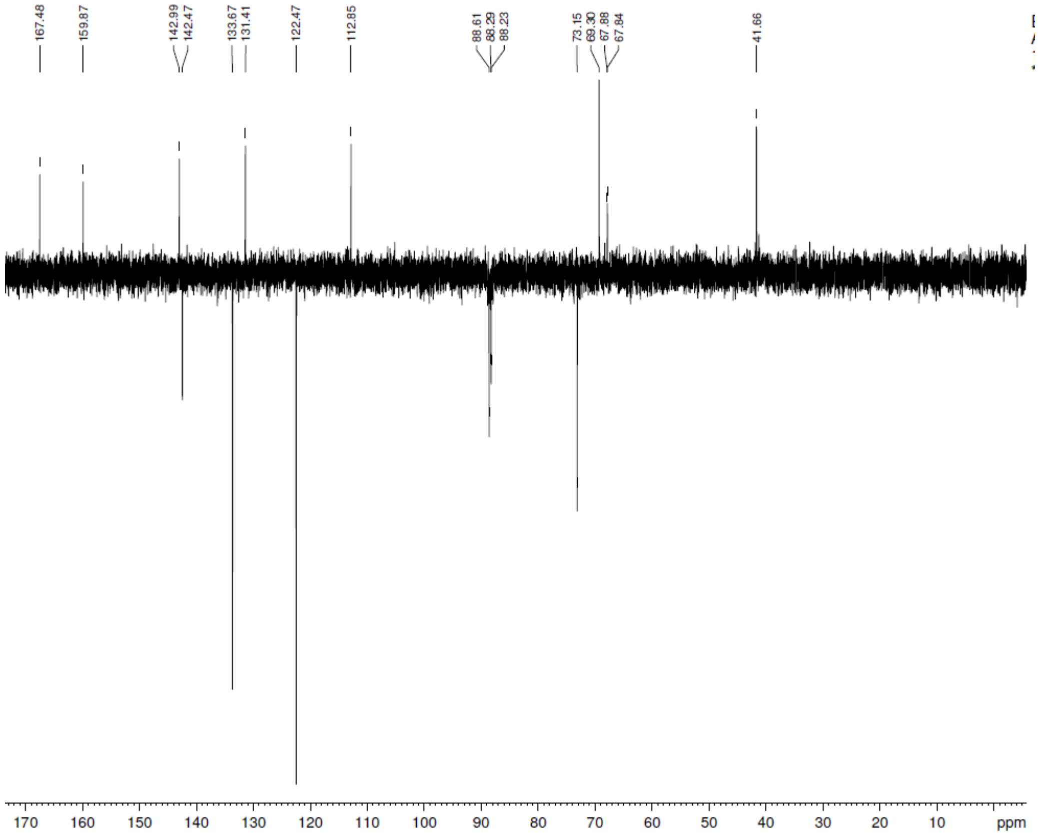

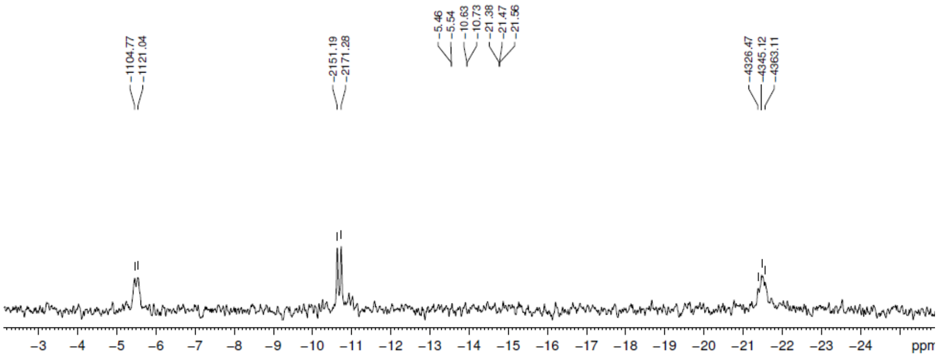

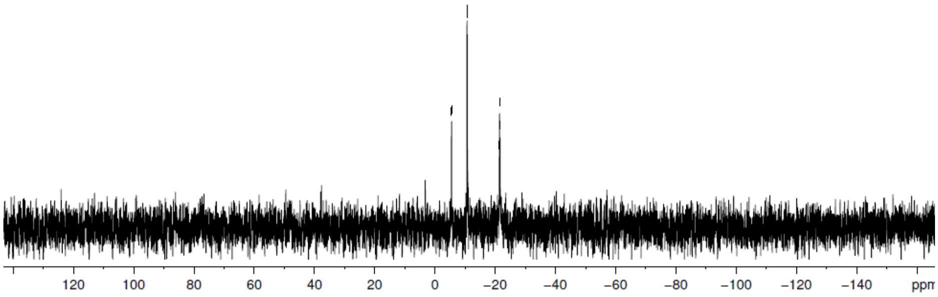

^{1}H NMR, ^{13}C and ^{31}P spectra of **dA$^{\mathbf{A}}$TP.**

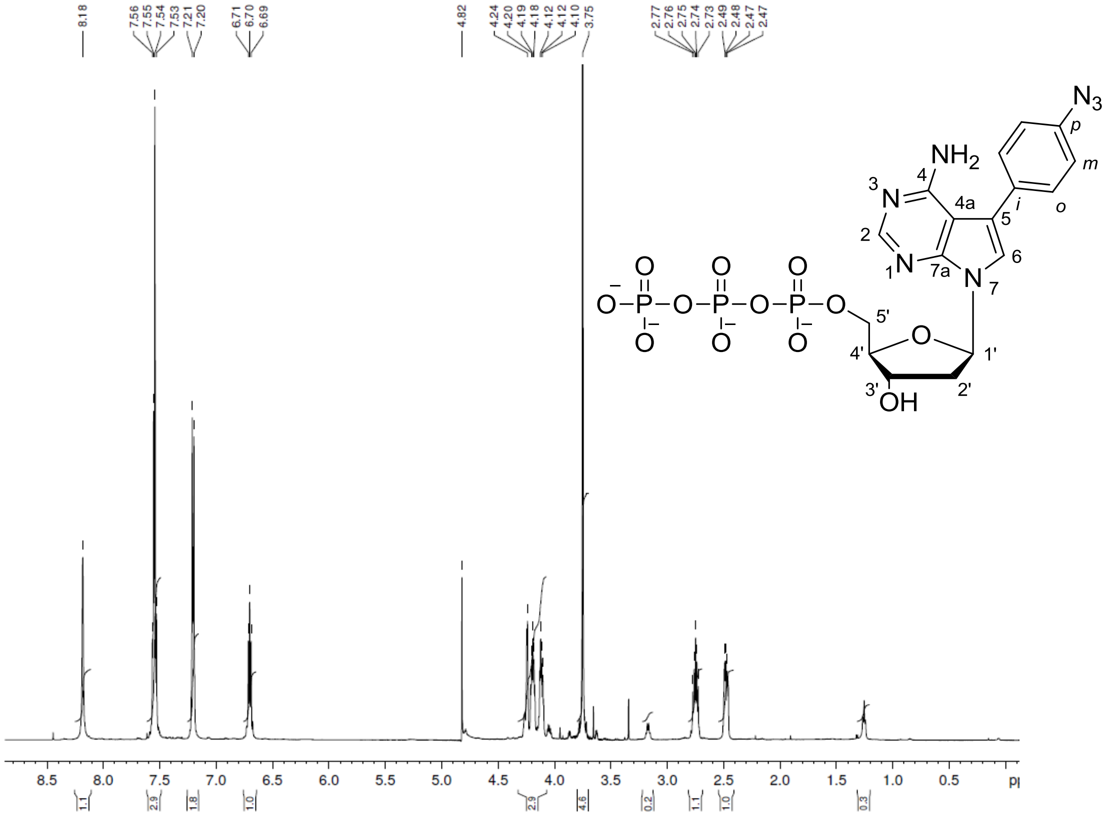

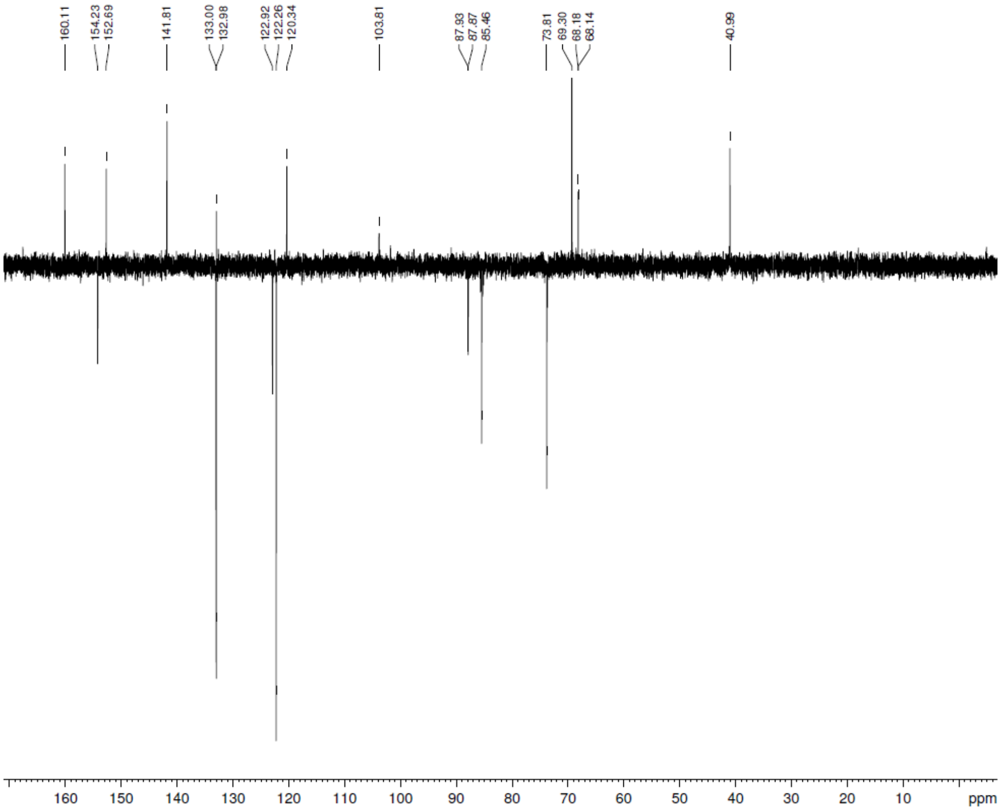
160.11
154.23
152.69
141.81
133.00
132.98
122.92
122.26
120.34
103.81
87.93
87.87
85.46
73.81
69.30
68.18
68.14
40.99
ppm

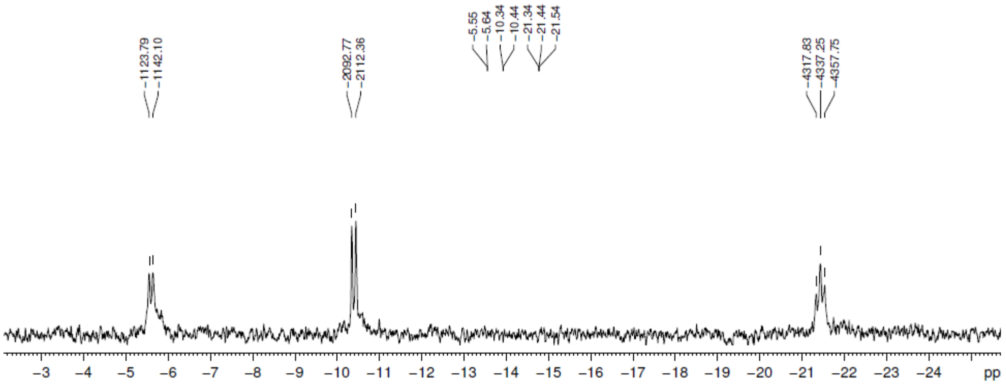
-1123.79
-1142.10
-2092.77
-2112.36
-5.55
-5.64
-10.34
-10.44
-21.34
-21.44
-21.54
-4317.83
-4337.25
-4357.75
pp

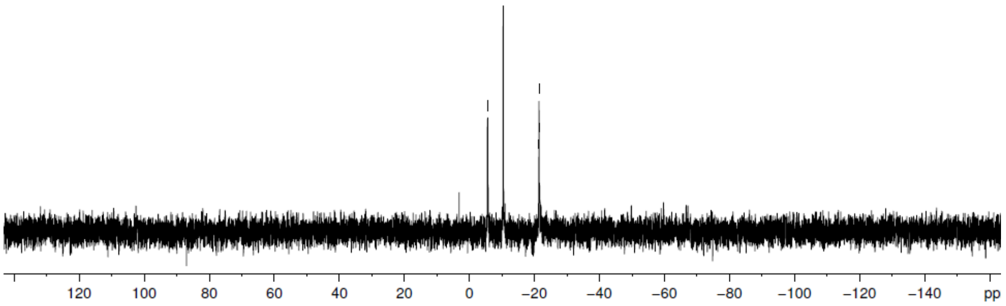
pp

^{1}H NMR, ^{13}C and ^{31}P spectra of **dC^{TP}TP.**

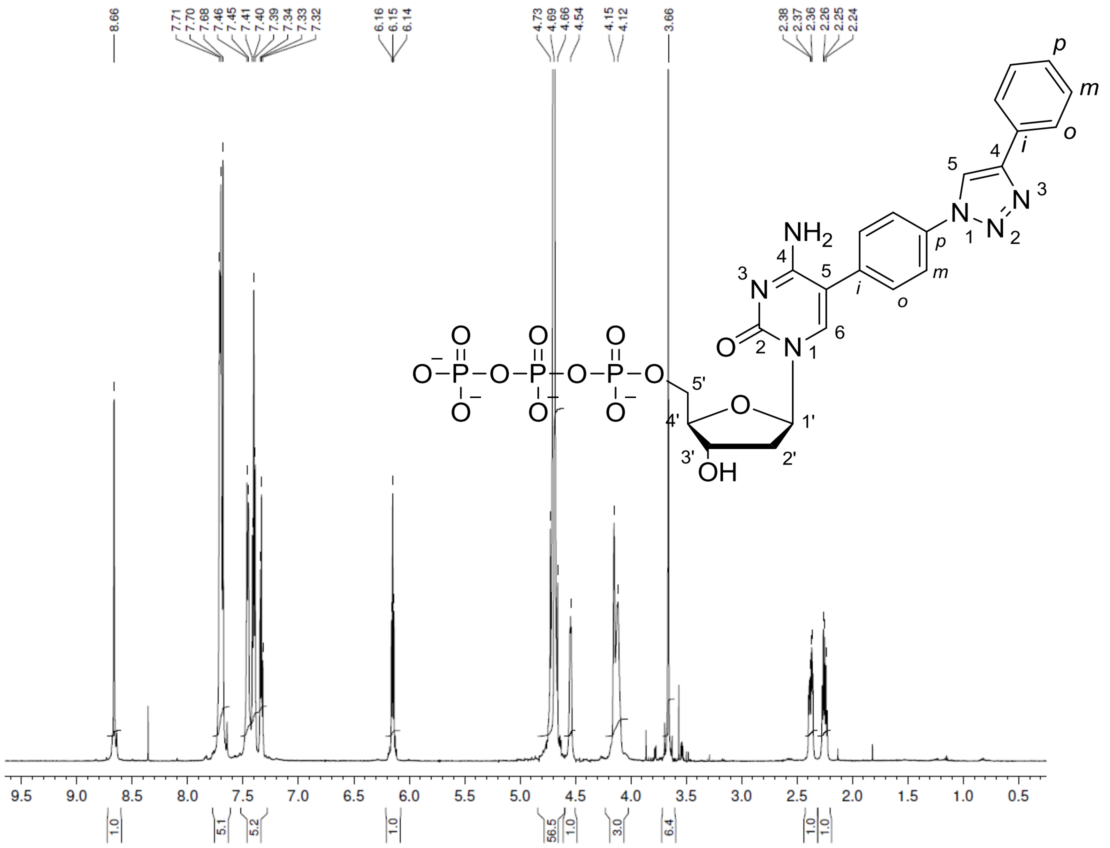

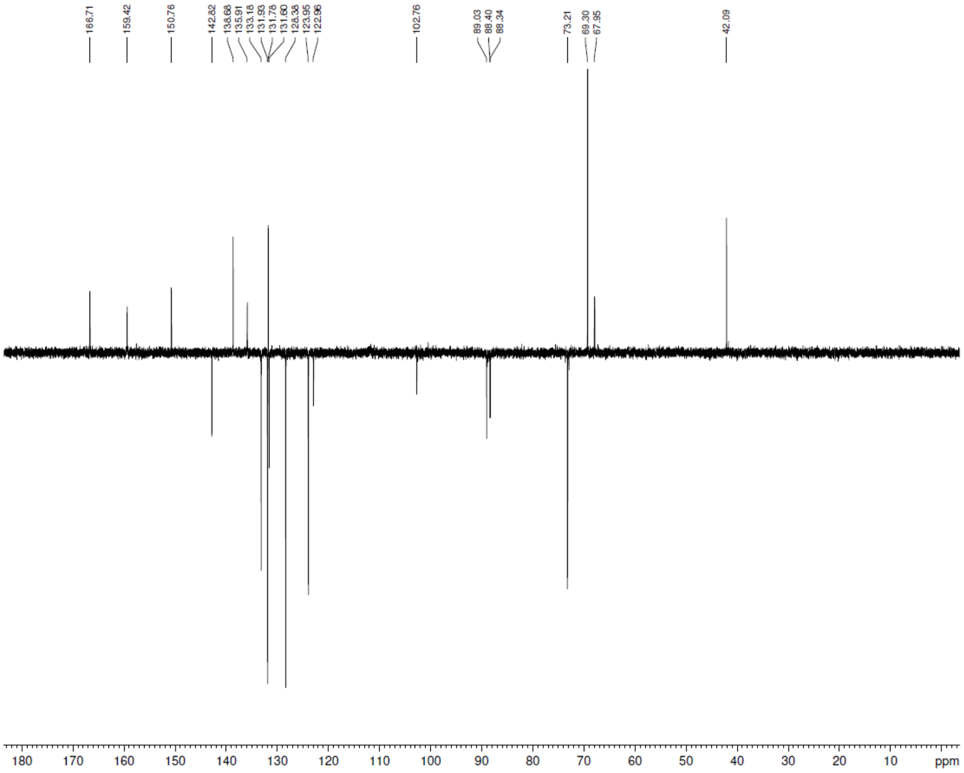

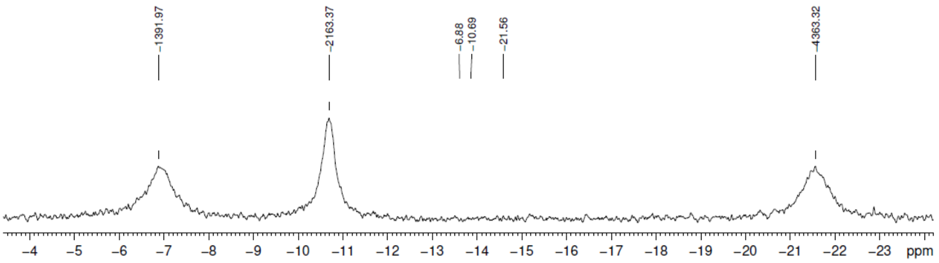

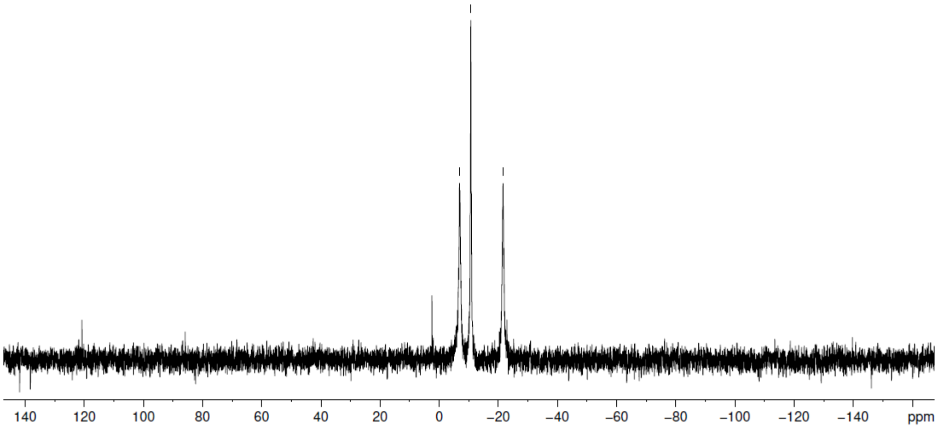

^{1}H NMR, ^{13}C and ^{31}P spectra of **dA^{TP}TP.**

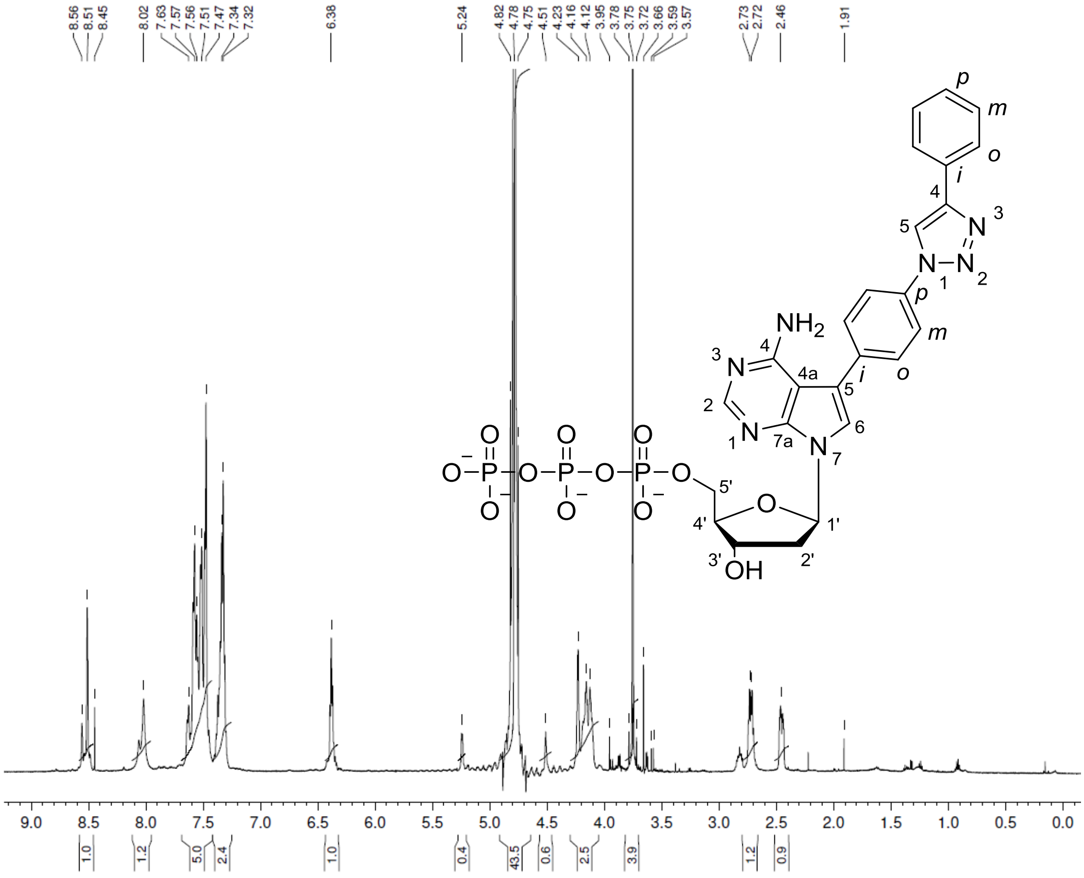

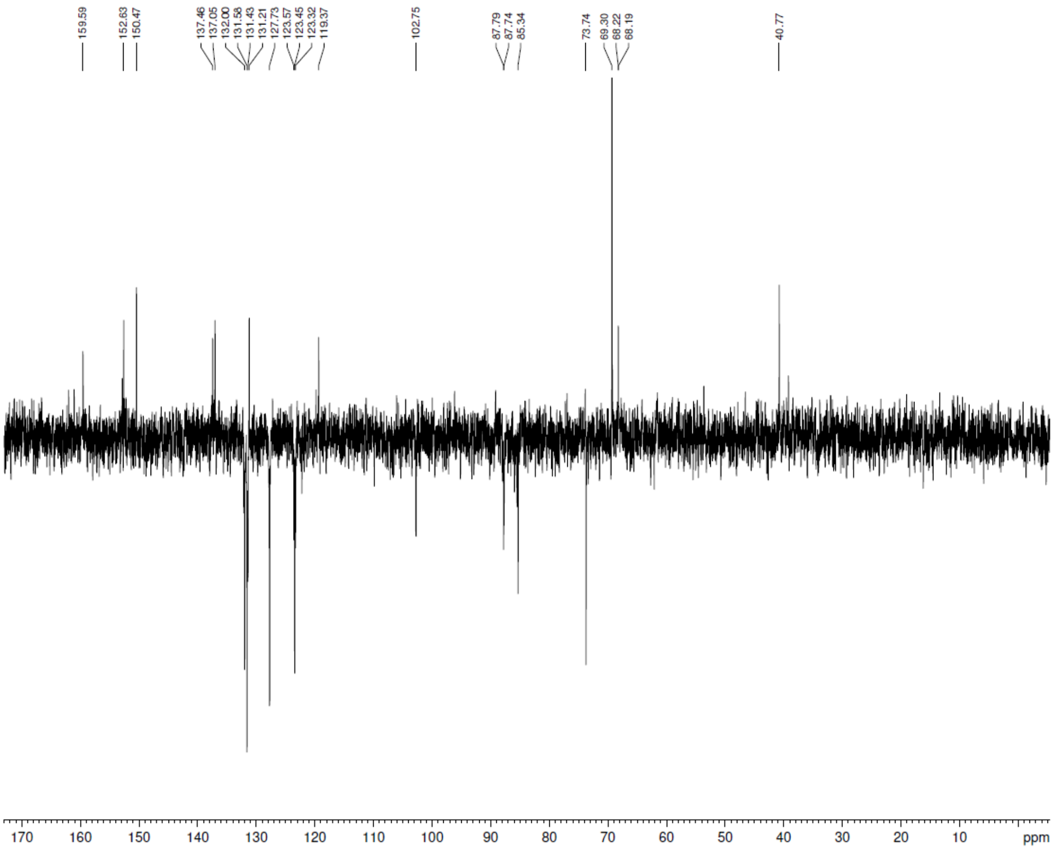
159.59
152.63
150.47
137.46
137.05
132.00
131.58
131.43
131.21
127.73
123.57
123.45
123.32
119.37
102.75
87.79
87.74
85.34
73.74
69.30
68.22
68.19
40.77
170 160 150 140 130 120 110 100 90 80 70 60 50 40 30 20 10 ppm

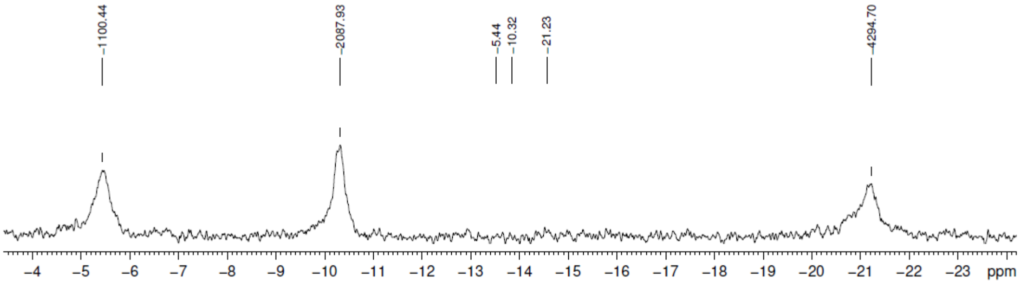
-1100.44
-2087.93
-5.44
-10.32
-21.23
-4294.70
−4 −5 −6 −7 −8 −9 −10 −11 −12 −13 −14 −15 −16 −17 −18 −19 −20 −21 −22 −23 ppm

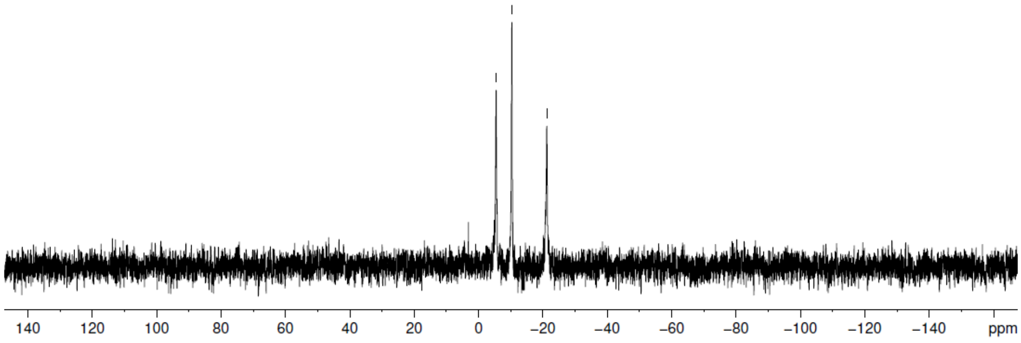
140 120 100 80 60 40 20 0 −20 −40 −60 −80 −100 −120 −140 ppm

^{1}H NMR, ^{13}C and ^{31}P spectra of **dC^{TNO2}TP.**

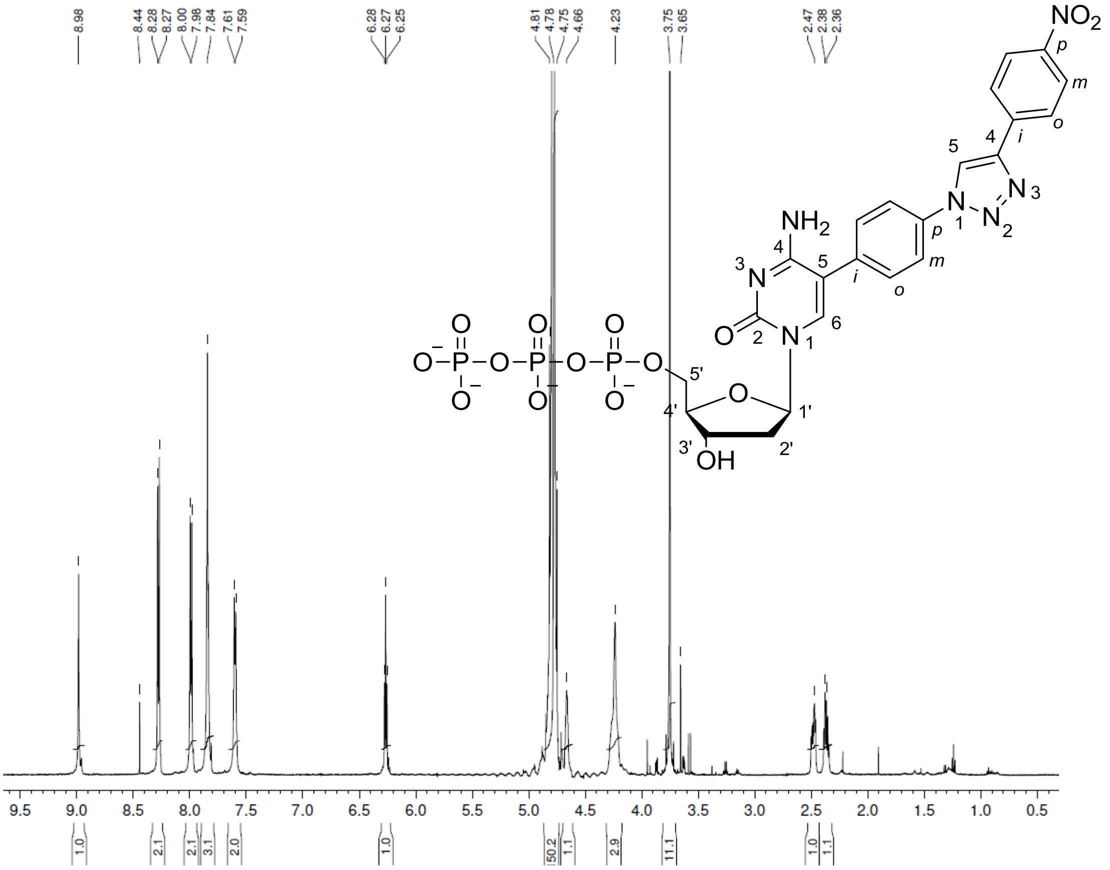

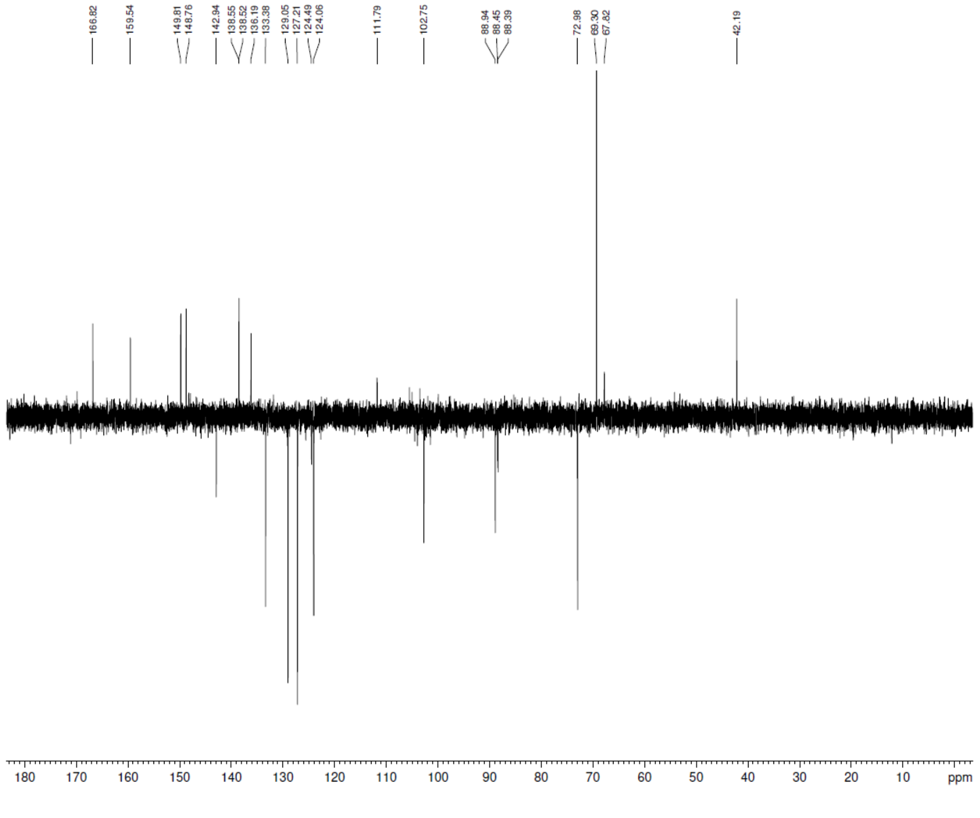

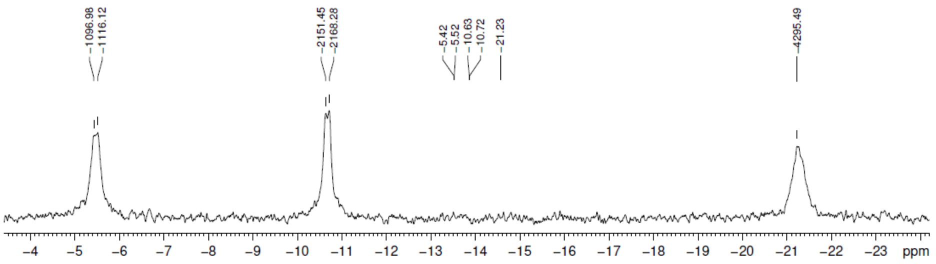

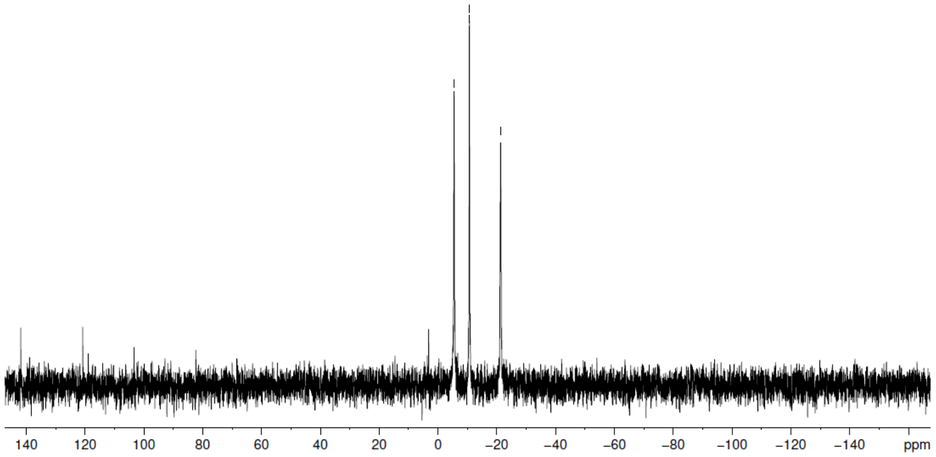

^{1}H NMR, ^{13}C and ^{31}P spectra of **dA^{TNO2}TP.**

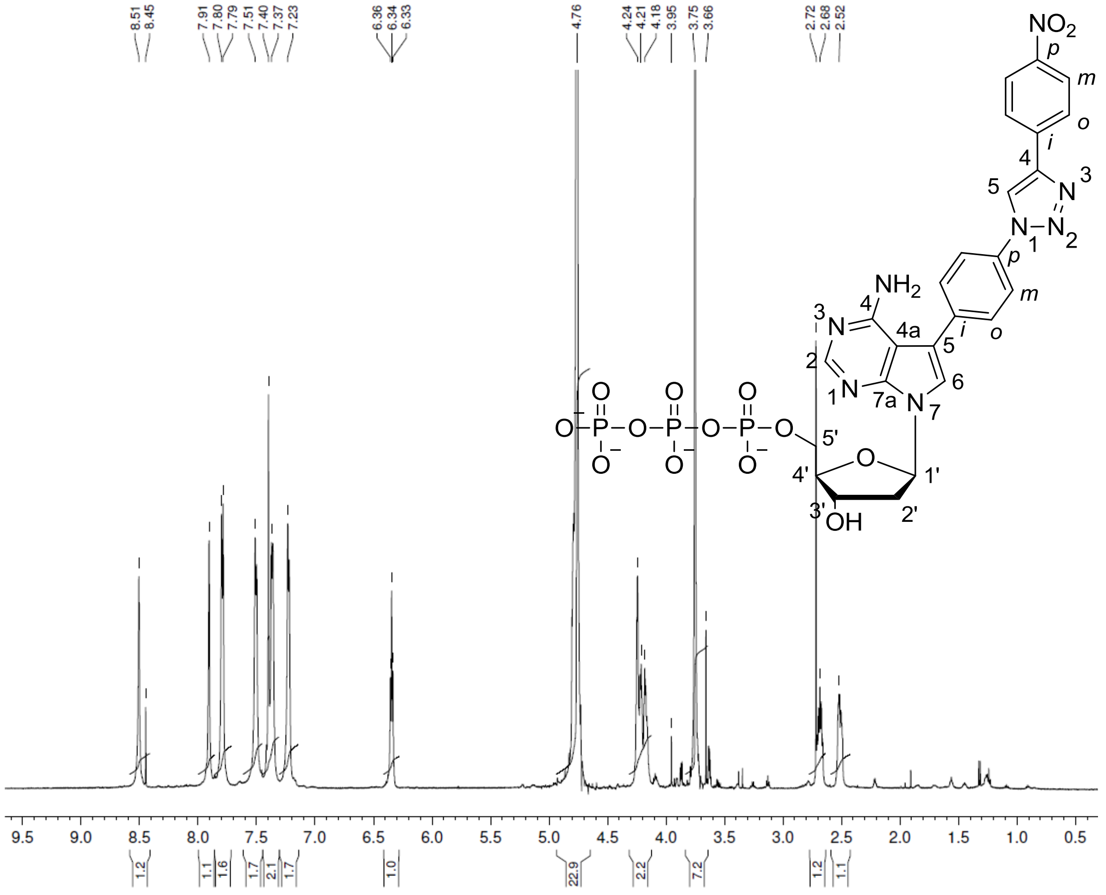

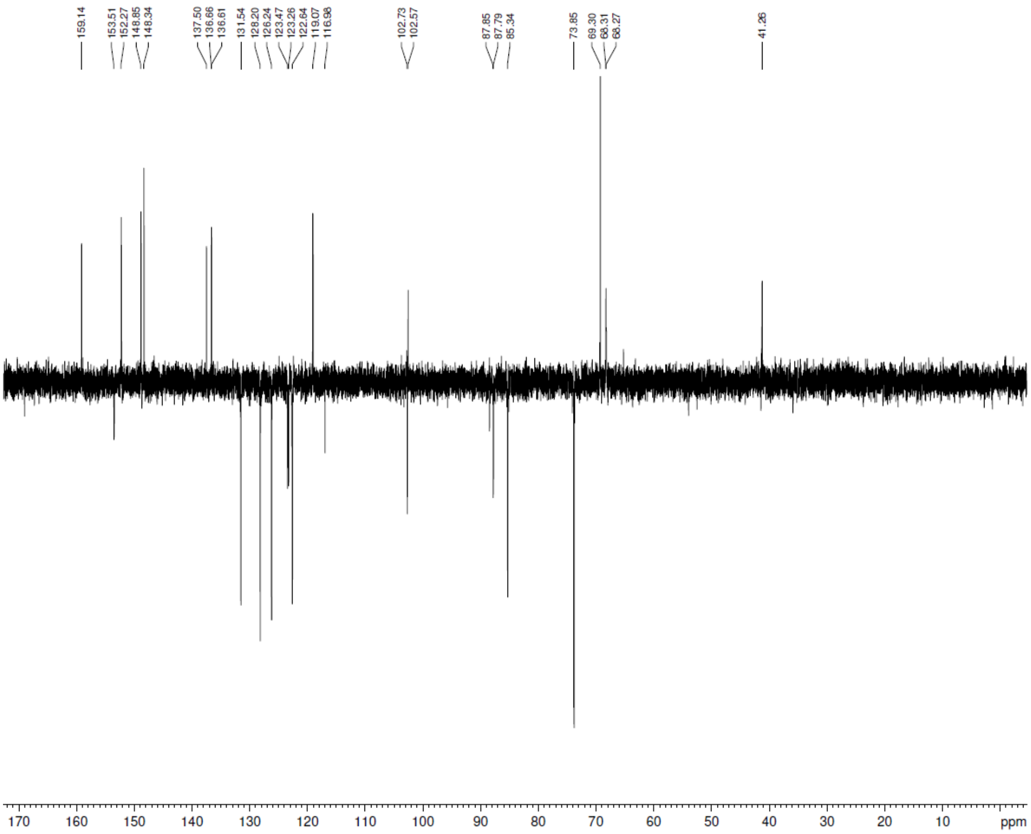
159.14
153.51
152.27
148.85
148.34
137.50
136.66
136.61
131.54
128.20
126.24
123.47
123.26
122.64
119.07
116.98
102.73
102.57
87.85
87.79
85.34
73.85
69.30
68.31
68.27
41.26
170 160 150 140 130 120 110 100 90 80 70 60 50 40 30 20 10 ppm

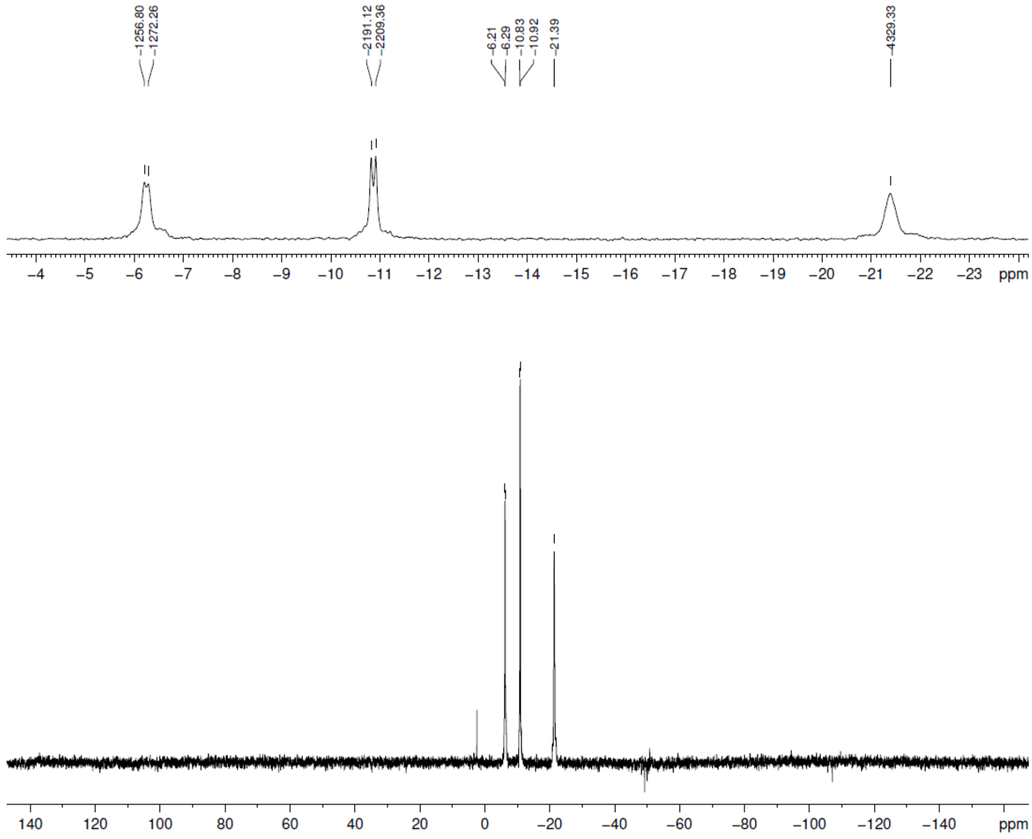
-1256.80
-1272.26
-2191.12
-2209.36
-6.21
-6.29
-10.83
-10.92
-21.39
-4329.33
−4 −5 −6 −7 −8 −9 −10 −11 −12 −13 −14 −15 −16 −17 −18 −19 −20 −21 −22 −23 ppm
140 120 100 80 60 40 20 0 −20 −40 −60 −80 −100 −120 −140 ppm

9 Copies of Maldi-TOF spectra of DNA after click reaction

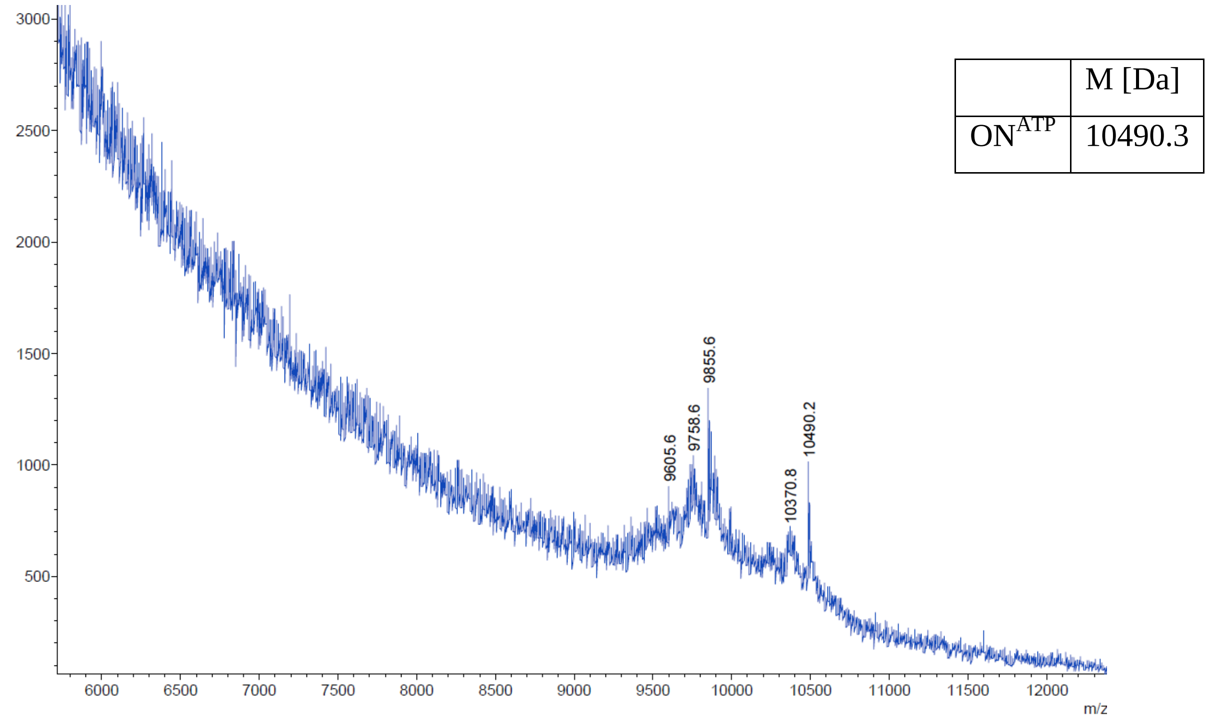

	M [Da]
ON^{ATP}	10490.3

Figure S21. MALDI-TOF MS spectrum of temp^{rnd16} with A^{TP} modification (31 nt).

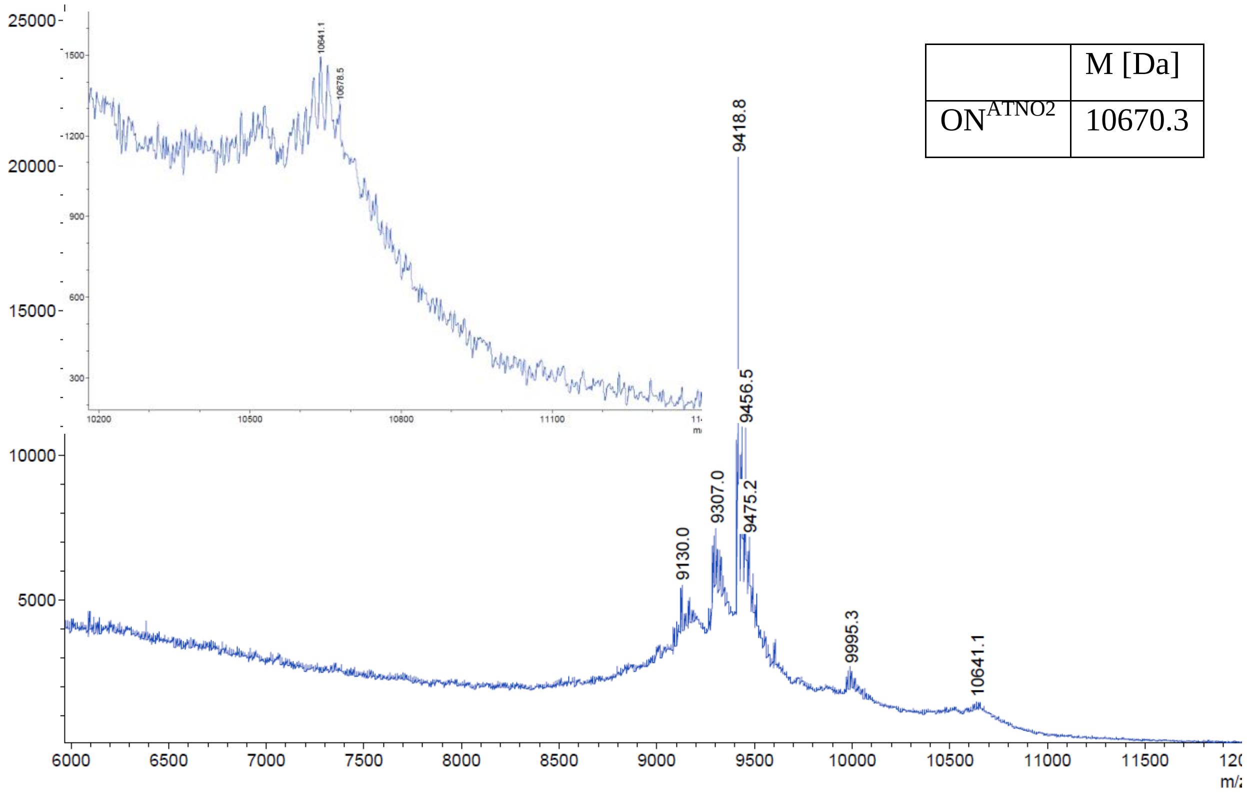

	M [Da]
ON^{ATNO2}	10670.3

Figure S22. MALDI-TOF MS spectrum of temp^{rnd16} with A^{TNO2} modification (31 nt)

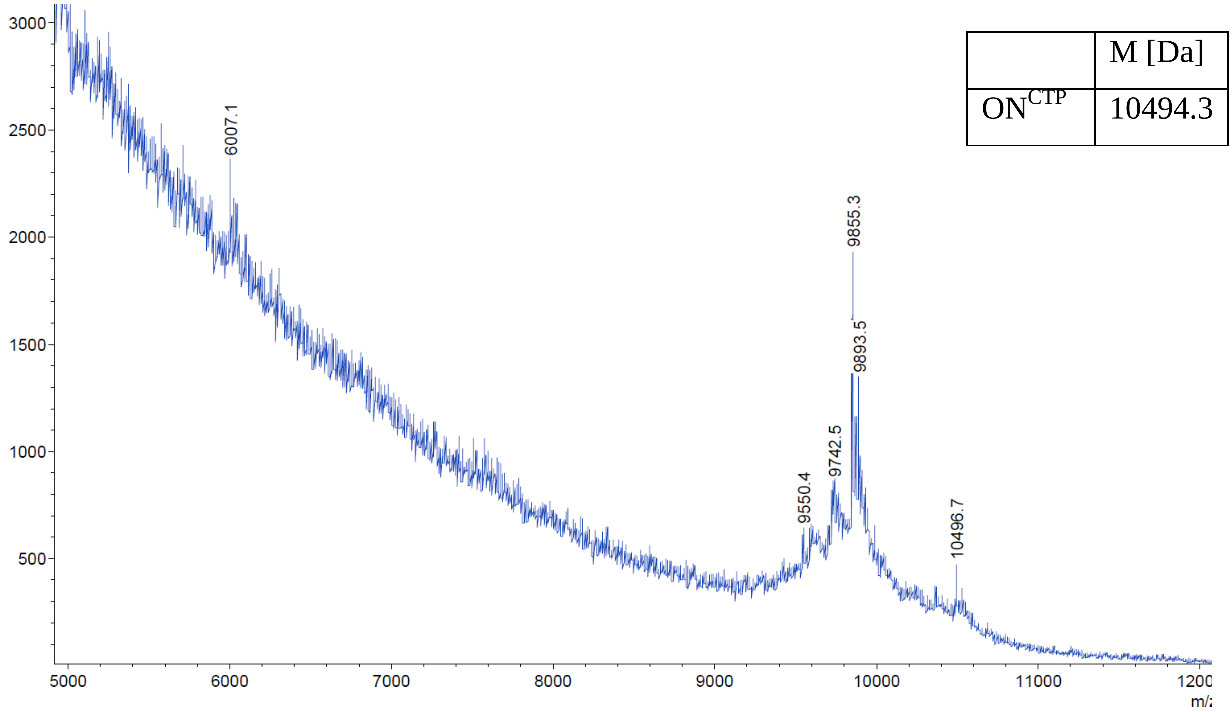

	M [Da]
ON^{CTP}	10494.3

Figure S23. MALDI-TOF MS spectrum of temp^{rnd16} with C^{TP} modification (31 nt).

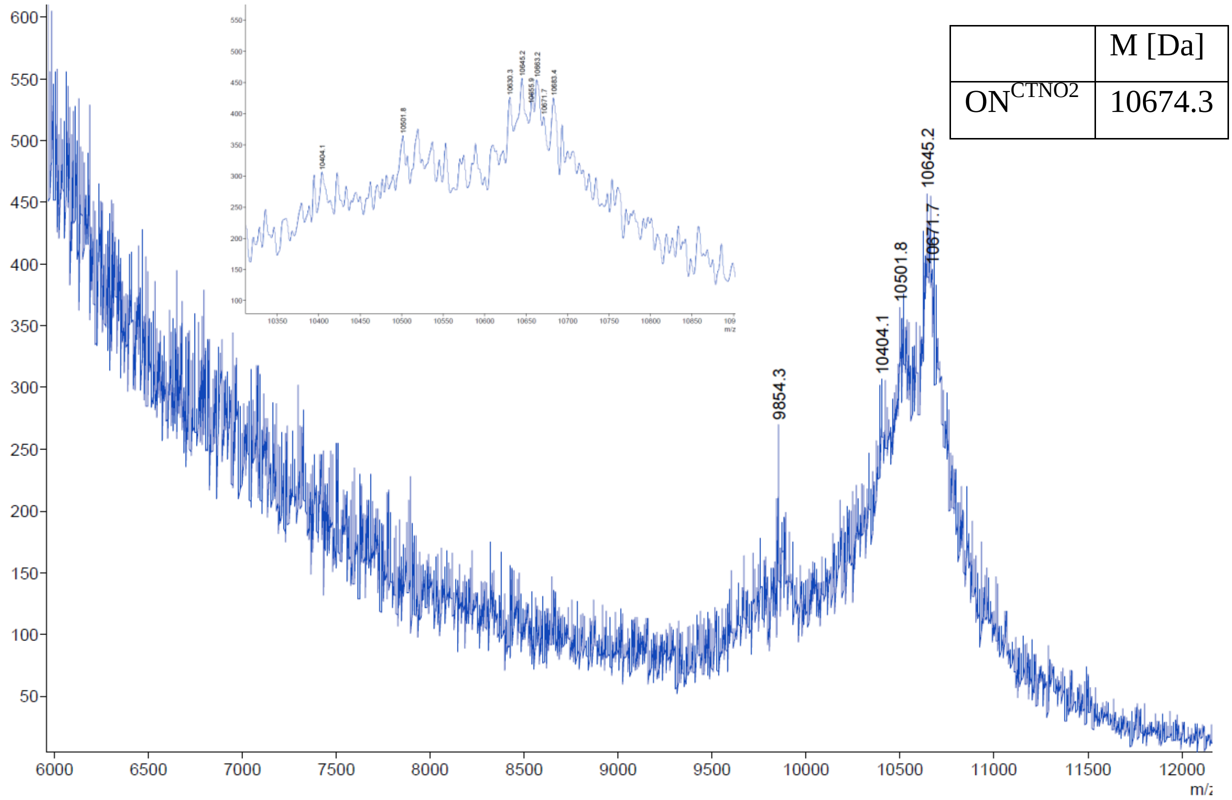

	M [Da]
ON^{CTNO2}	10674.3

Figure S24. MALDI-TOF MS spectrum of temp^{rnd16} with C^{TNO2} modification (31 nt).

10 Copies of Maldi-TOF spectra

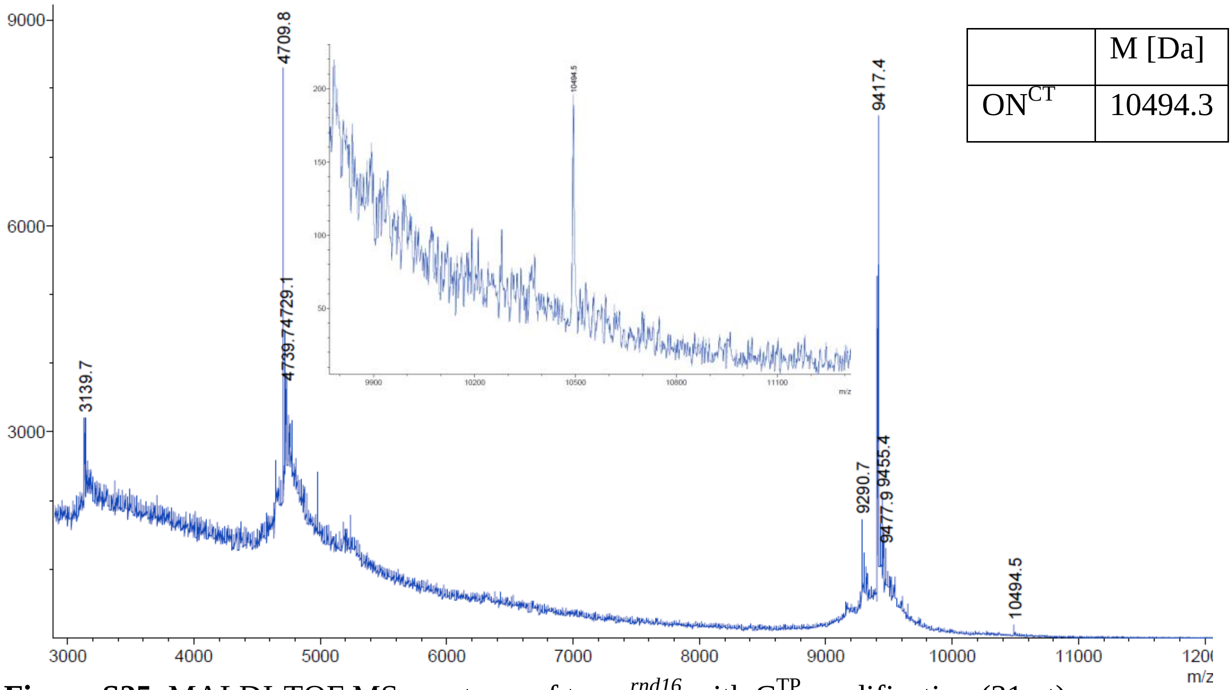

	M [Da]
ON^{CT}	10494.3

Figure S25. MALDI-TOF MS spectrum of temp^{rnd16} with C^{TP} modification (31 nt)

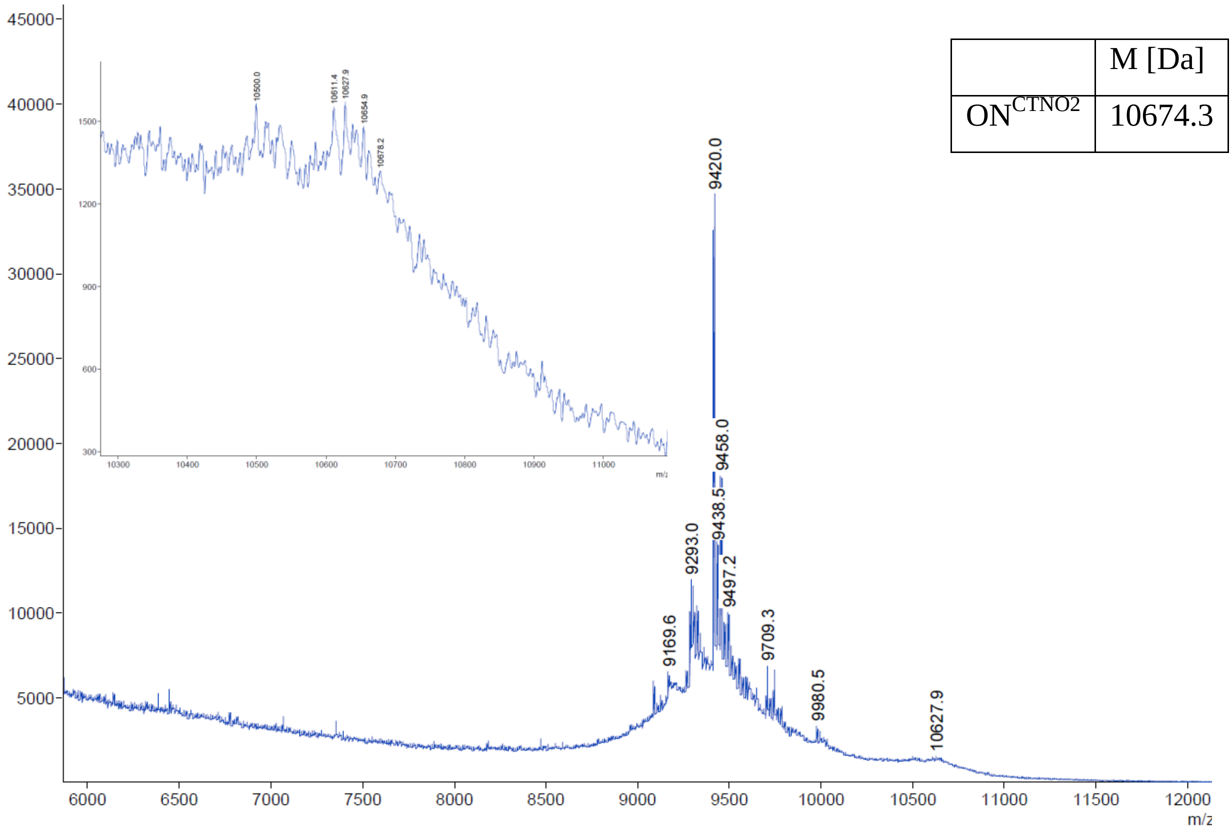

	M [Da]
ON^{CTNO2}	10674.3

Figure S26. MALDI-TOF MS spectrum of temp^{rnd16} with C^{TNO2} modification (31 nt).

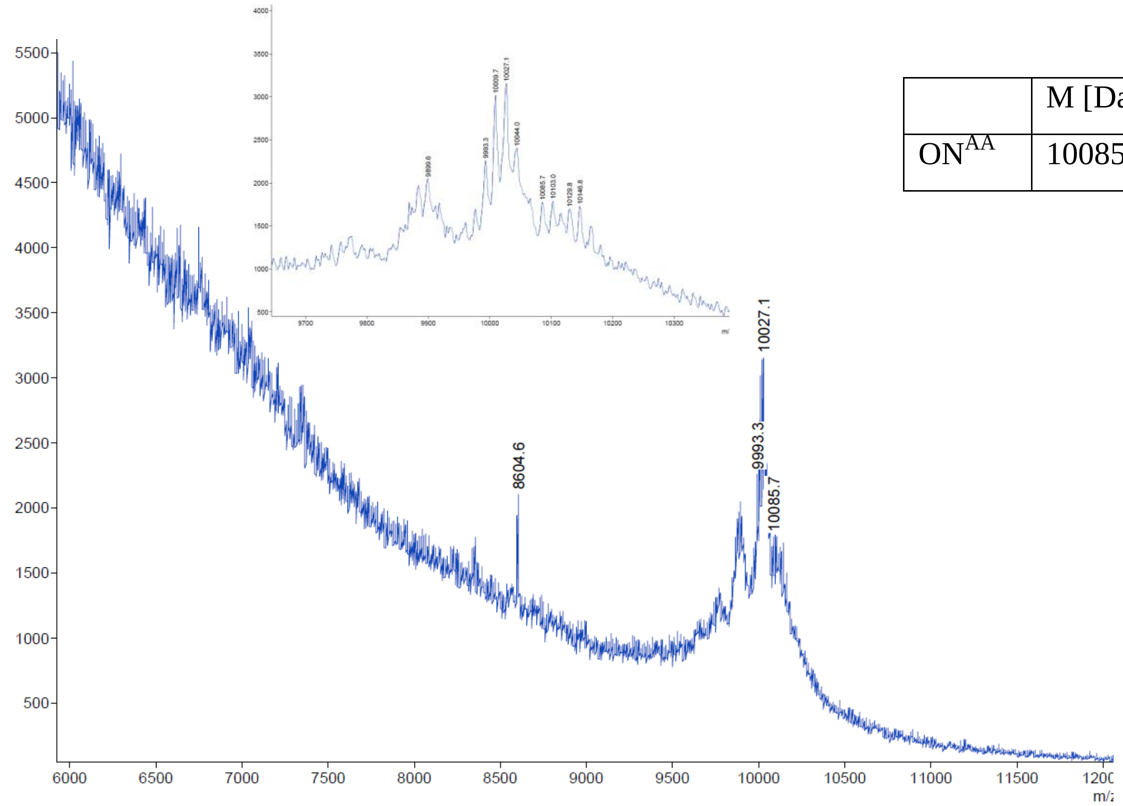

	M [Da]
ON^{AA}	10085.8

Figure S27. MALDI-TOF MS spectrum of temp^{rnd16} with A^{A} modification (31 nt).

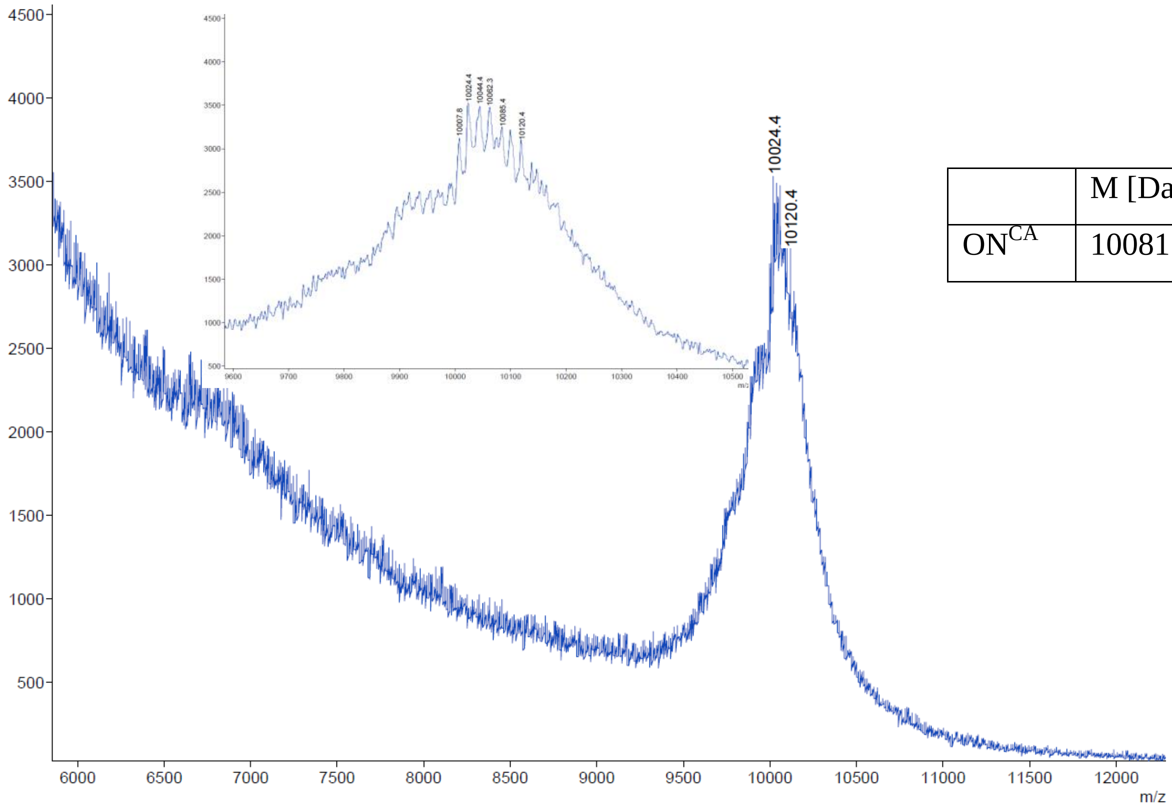

	M [Da]
ON^{CA}	10081.8

Figure S28. MALDI-TOF MS spectrum of temp^{rnd16} with C^{A} modification (31 nt).
